# *OsDUF1223* negatively regulates seedling drought tolerance and modulates nitrate-dependent root growth in rice

**DOI:** 10.1371/journal.pgen.1012295

**Published:** 2026-09-03

**Authors:** Xiulin Zhao, Ziyi Wang, Mingfei Chen, Lingxiang Lu, Hui Lin, Xiaofei Zan, Xiaomei Jia, Xiaoying Ye, Rongjun Chen, Jianqing Zhu, Jun Zhu, Lihua Li

**Affiliations:** 1 State Key Laboratory of Crop Gene Exploration and Utilization in Southwest China, Rice Research Institute, Sichuan Agricultural University, Chengdu, China; 2 Nanchong Academy of Agricultural Sciences, Nanchong, Sichuan, China; 3 Crop Ecophysiology and Cultivation Key Laboratory of Sichuan Province, Chengdu, China; University of Kentucky, UNITED STATES OF AMERICA

## Abstract

Rice (*Oryza sativa* L.) is a major staple food crop worldwide. Drought stress induced by extreme weather severely limits its growth and yield. Proteins containing domains of unknown function (DUF) play important roles in plant stress responses, but their regulatory mechanisms remain largely unclear. In this study, we demonstrate that the plasma membrane-localized protein *OsDUF1223* functions as a negative regulator of drought tolerance in rice seedlings. Under drought stress, *OsDUF1223*-overexpressing lines exhibited lower survival rates, higher water loss rates, and reduced antioxidant enzyme activities and osmolyte accumulation. Transcriptomic and physiological analyses revealed that *OsDUF1223* modulates drought responses through ABA signaling and nitrogen response pathways. Collectively, this study elucidates that *OsDUF1223* regulates drought tolerance at the seedling stage in rice through functional association with the ABA and nitrogen signaling pathways, thereby providing a candidate gene resource for the genetic improvement of drought tolerance in rice.

## Author summary

Rice is a major staple food crop that feeds nearly half of the world's population. However, drought stress severely limits its growth and yield. As climate change increases the frequency and severity of extreme drought events, developing drought-resistant rice varieties has become critical for food security. Plants respond to drought through complex signaling networks, yet the functions of many proteins within these networks remain unclear. In this study, we discovered that a rice protein named *OsDUF1223* negatively regulates drought tolerance at the seedling stage. When this protein is overexpressed, rice plants show lower survival rates, faster water loss, and reduced antioxidant capacity under drought conditions. Through gene expression analysis and physiological experiments, we found that *OsDUF1223* modulates drought responses through the hormone abscisic acid signaling and nitrogen response pathways. Our findings provide a candidate gene resource for breeding drought-resistant rice and deepen our understanding of how proteins of previously unknown function contribute to plant stress adaptation.

## Introduction

Rice (*Oryza sativa* L.), an annual gramineous crop, serves as the staple food for approximately 50% of the global population and plays a pivotal role in worldwide food security, especially in densely populated regions where it acts as the primary source of energy and nutrition [1,2]. However, rice growth and development are highly vulnerable to abiotic stresses, particularly water deficit. In recent years, global warming has disrupted precipitation patterns, leading to increased frequency and severity of extreme drought events. It is projected that more than 70% of global land areas may face drought threats by the end of the 21st century [3]. Drought has thus become a major environmental factor limiting rice growth, development and yield. Elucidating the response mechanisms of rice to drought stress is therefore of great significance for breeding stress-resistant varieties and improving crop productivity.

Plant drought response is a complex multi-layer regulatory network involving signal perception, transduction, and physiological and biochemical responses [4]. Changes in external water conditions initiate specific signaling pathways modulated by stress intensity and perception sites, including abscisic acid (ABA), jasmonic acid (JA), and ethylene-dependent pathways [5]. Inter-tissue and inter-organ signal communication ultimately enhances plant drought tolerance [6]. Studies have demonstrated that ABA accumulation in mesophyll cells effectively improves drought resistance [7]. ABA remodels root architecture to adapt plants to drought and waterlogging stresses [8], and promotes symbiosis with rhizosphere microorganisms, thereby facilitating nutrient uptake, enhancing stress tolerance, and indirectly regulating plant growth [9,10]. Under adverse conditions, ABA-induced stomatal closure serves as a core mechanism for water conservation and defense against pathogen invasion [11,12]. Moreover, ABA modulates stomatal density, stomatal conductance, and the expression of stress-related genes, playing a pivotal role in plant adaptation to abiotic stresses such as salinity [13,14]. For instance, the rice *OsJAZ9* gene reduces stomatal density and markedly elevates drought tolerance [15].

Roots serve as the primary sensory site for drought signal perception [16–18]. Under water deficit, plants remodel root architecture through adaptive morphological differentiation to adapt to heterogeneous soil water distribution. Roots with longer length and smaller branching angles can efficiently absorb water from dry topsoil while utilizing moisture in deeper soil layers [19]. Moreover, drought-induced cellular water loss suppresses cell elongation and expansion [20]. Leaf stomata regulate their opening and closing depending on guard cell turgor pressure, and stomatal closure represents the fastest physiological response of plants to reduce transpiration and prevent dehydration. Studies have demonstrated that the XER gene in Arabidopsis [21] and *VvWRKY18* in grape negatively regulate drought tolerance by increasing stomatal density [22].

At the cellular level, reduced turgor pressure triggers the accumulation of osmoprotectants such as soluble sugars, phenolics, and free amino acids, which is one of the key drought-resistant mechanisms in rice [4]. Proline accumulation helps maintain leaf turgor and stomatal conductance [1], whereas soluble sugars play a critical role in balancing photosynthesis and mitochondrial respiration [23]. However, drought stress also induces excessive production of reactive oxygen species (ROS), disrupting cellular redox homeostasis [24]. Excess ROS causes systemic oxidative stress, leading to lipid peroxidation and macromolecular damage, thereby inhibiting growth and triggering cell death [25,26]. Plants eliminate excess ROS via enzymatic antioxidant systems such as superoxide dismutase (SOD), alleviating subsequent cellular damage [26,27].

As an essential macronutrient for plants, nitrogen is involved throughout the entire life cycle of rice, including seed germination, morphogenesis, root development and senescence [28–30], and also serves as a key factor limiting plant growth, development and yield. Nitrate and other nitrogen compounds act not only as nutrients but also as signaling molecules that regulate gene expression and participate in plant growth, development and adaptation to abiotic stresses [31]. Nitrate and ammonium are the primary inorganic nitrogen forms taken up and utilized by higher plants, among which NO_3^-^_ is the major nitrogen source for the majority of non-leguminous plants [32,33]. Plants complete NO_3_^-^uptake through transporters localized on the root cell plasma membrane, after which it is further reduced to ammonium and incorporated into organic nitrogen compounds, ultimately forming amino acids, proteins, and other nitrogen-containing compounds [34,35]. In addition, nitrogen and its metabolites also act as signaling molecules that regulate the expression of nitrogen-responsive genes and activate signaling pathways involved in the regulation of diverse biological processes. As the primary organ for nitrogen uptake, roots exhibit remarkable structural plasticity in response to changes in the form, abundance, and distribution of soil nitrogen. Nitrogen-dependent regulation of root system architecture (RSA) relies on the integration of local and systemic nitrogen signals [36], and plant hormones also play a critical role in systemic nitrogen signal transduction [37–39].

Proteins containing domains of unknown function (DUF) are a group of proteins with conserved amino acid sequences but incompletely characterized functions [40]. Although DUF family genes are generally not essential for plant survival, numerous studies have demonstrated their vital regulatory roles in plant growth, development, and stress adaptation [41]. In recent years, rapid advances in genomics and transcriptomics have greatly promoted functional studies of the DUF family. For instance, the maize DUF538 family is involved in root development and leaf stress responses [42], while rice *OsDUF936.6* and *OsDUF846.2* have been identified as negative regulators of salt tolerance [43,44]. Nevertheless, the full biological functions of the DUF family remain to be further elucidated.

This study focused on the function of *OsDUF1223* in the drought stress response of rice seedlings. Using transcriptome sequencing and quantitative real-time PCR, we analyzed its regulatory network and obtained evidence suggesting that *OsDUF1223* may be associated with drought tolerance through its functional links to nitrogen and ABA signaling pathways. This work not only identifies a novel candidate drought-tolerant gene for molecular breeding, but also deepens our understanding of the drought stress regulatory network and the functional roles of DUF family proteins in rice.

## Results

### Bioinformatics analysis

The gene encoding *OsDUF1223* is located on chromosome 7, with an open reading frame encoding 274 amino acid residues and a predicted molecular weight of approximately 28.97 kDa. To investigate the evolutionary conservation of the *OsDUF1223* protein, we analyzed DUF1223 family members from rice (*Oryza sativa* L.), *Arabidopsis thaliana* (L.) Heynh., wheat (*Triticum aestivum* L.), barley (*Hordeum vulgare* L.), cucumber (*Cucumis sativus* L.), soybean (*Glycine max* (L.) Merr.), tomato (*Solanum lycopersicum* L.), white poplar (*Populus tomentosa* Carrière), and maize (*Zea mays* L.). Phylogenetic analysis revealed strong sequence conservation among these orthologs (S1A Fig).

### *OsDUF1223* is specifically expressed at the seedling stage and responds to both drought stress and exogenous ABA

**Fig 1:**
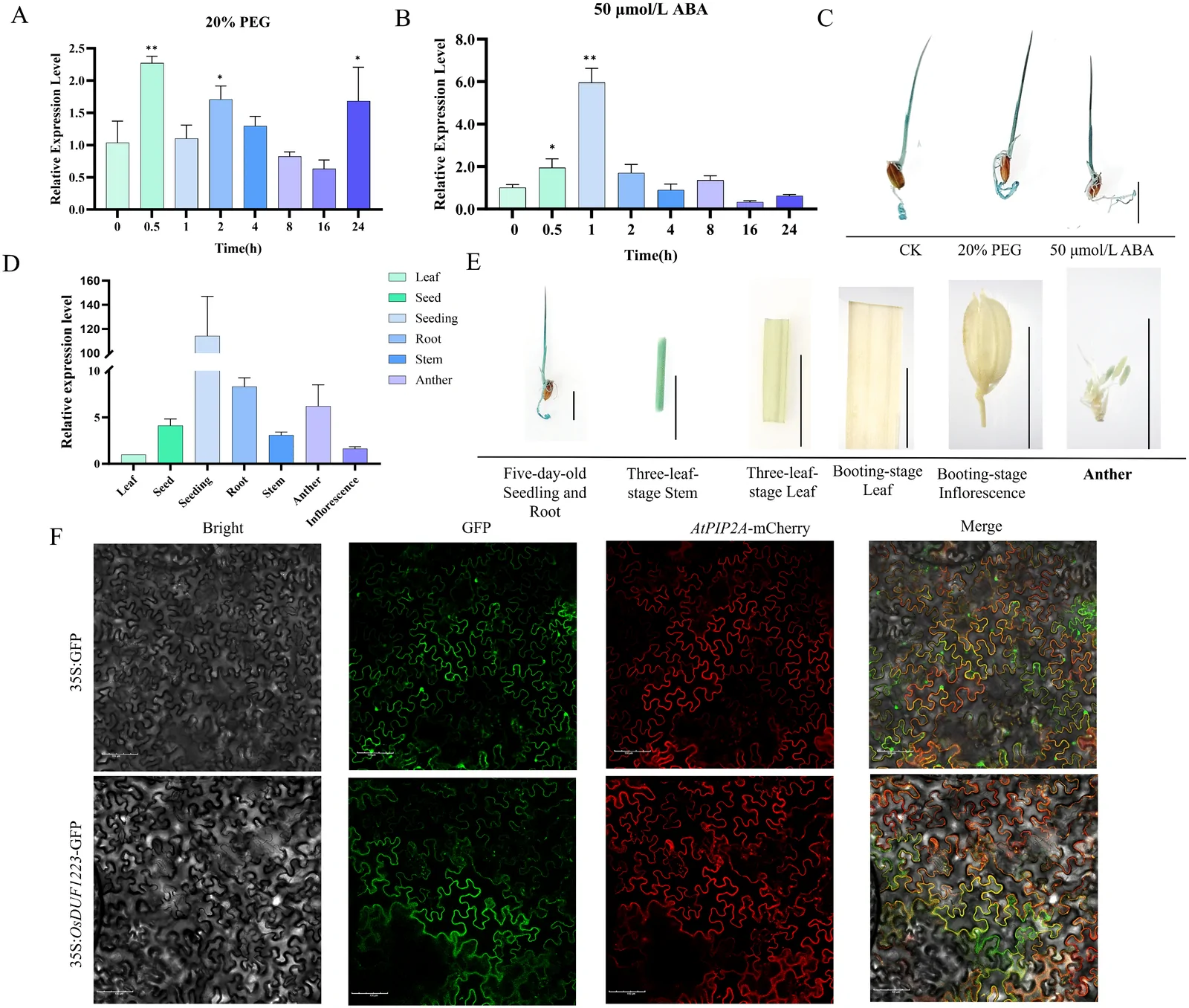
Expression pattern analysis of the *OsDUF1223* gene and subcellular localization of its protein. Note: A–B. Relative transcript levels of the *OsDUF1223* gene after 24 h of treatment with 20% PEG and 50 μmol/L ABA, respectively (n = 3, technical replicates); C. GUS staining results of the *OsDUF1223* gene after 0.5 h of treatment with 20% PEG and 50 μmol/L ABA, respectively. Scale bar = 2 cm; D. Relative transcript levels of the *OsDUF1223* gene in different spatiotemporal tissue regions (n = 3, technical replicates); E. GUS staining results of the *OsDUF1223* gene in different spatiotemporal tissue regions. Scale bar = 2 cm; F. Subcellular localization of the *OsDUF1223*-GFP fusion protein. Scale bar = 100 μm. Data are presented as mean ± SEM and were analyzed by one-way ANOVA with Tukey's multiple comparison test (A–B). * indicates a significant difference (0.01 < *P* < 0.05), ** indicates an extremely significant difference (*P* < 0.01). GUS staining (C, E) and subcellular localization (F) were performed with three independent biological replicates, all yielding consistent results; representative images are shown.

Based on publicly available transcriptomic data and subsequent experimental validation from rice seedlings under drought and ABA treatments, we hypothesized that *OsDUF1223* may be involved in drought stress and ABA signal responses. RT-qPCR analysis revealed that in three-leaf stage wild-type rice seedlings treated with 20% PEG (simulated drought) or 50 μmol/L exogenous ABA, the transcript level of *OsDUF1223* increased at 0.5 h, 2 h, and 24 h under PEG treatment, peaking at 0.5 h (Fig 1A). Under ABA treatment, its expression first increased and then decreased, reaching a maximum at 1 h that was 5.96-fold higher than that in the untreated control (Fig 1B), suggesting a role in ABA-mediated drought responses. To verify these results, histochemical staining was performed using pro*OsDUF1223*::GUS transgenic plants in the Nipponbare background (Fig 1C). GUS signals in the shoots of 5-day-old seedlings were significantly enhanced after 0.5 h of stress treatment compared with normal conditions, consistent with RT-qPCR data and providing visual tissue-level evidence for its responsiveness to drought and ABA signals.

Furthermore, to characterize the spatiotemporal expression pattern of *OsDUF1223*, its transcript abundance was examined in various tissues and developmental stages of wild-type rice via RT-qPCR and GUS staining. *OsDUF1223* exhibited distinct spatiotemporal specificity (Fig 1D–1E): expression was highest in seedling shoots, with specific accumulation in stems at the three-leaf stage. At the booting stage, only weak expression was detected in anthers, with little to no expression in other tissues, indicating that *OsDUF1223* functions mainly at the seedling stage.

### Generation of *OsDUF1223* transgenic plants and subcellular localization of its protein

In this study, we obtained *OsDUF1223* overexpression homozygous lines OE-1, OE-4, and OE-5 in the Nipponbare background, as well as knockout homozygous lines KO-1 and KO-2 in the Zhonghua 11 background (S1B–S1D Fig). The sequencing results showed that all knockout lines carried a 1 bp deletion at the target site, resulting in a frameshift mutation of *OsDUF1223* and leading to premature translation termination. To investigate the subcellular localization of the *OsDUF1223* protein, we first performed topological structure prediction. SignalP 6.0 analysis indicated that the protein lacks an N-terminal or C-terminal signal peptide; TMHMM 2.0 predicted that it contains no transmembrane helices and exhibits a predominantly hydrophilic conformation (S2 Fig). This topological feature is consistent with peripheral membrane-associated soluble proteins, distinct from integral membrane proteins, suggesting that *OsDUF1223* may localize to the plasma membrane in a non-transmembrane manner. To this end, we conducted co-localization assays in *Nicotiana benthamiana* epidermal cells by co-expressing 35S:*OsDUF1223*-GFP with the plasma membrane marker *AtPIP2A*-mCherry. As shown in Fig 1F, under confocal microscopy, the *OsDUF1223*-GFP signal (green) and the *AtPIP2A*-mCherry signal (red) exhibited substantial overlap and significant co-localization at the plasma membrane. Quantitative co-localization analysis (S1 Table) revealed a Pearson's correlation coefficient of 0.835, with Manders’ coefficients of M1 = 0.809 (GFP overlapping with RFP) and M2 = 0.802 (RFP overlapping with GFP). The topological predictions and co-localization experimental results corroborate each other, collectively demonstrating that *OsDUF1223* localizes to the plasma membrane as a peripheral membrane-associated soluble protein, rather than being embedded in the lipid bilayer as an integral membrane protein.

### *OsDUF1223* reduces drought tolerance in rice seedlings

**Fig 2:**
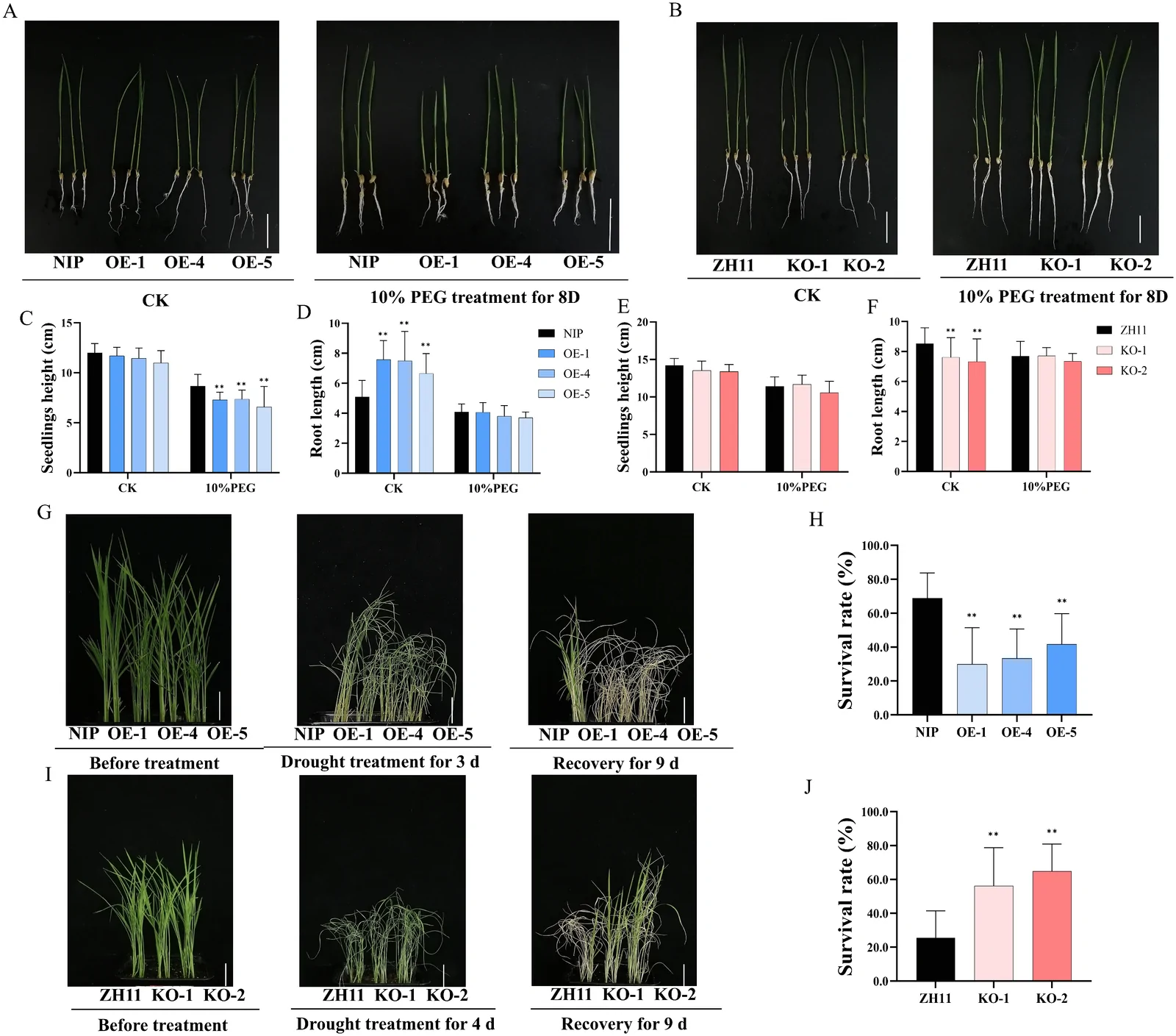
Drought stress tolerance of *OsDUF1223* transgenic seedlings. Note: A. Growth phenotypes of Nipponbare WT and OE lines under normal conditions and 10% PEG treatment (representative images shown). Scale bar = 4 cm; B. Growth phenotypes of Zhonghua 11 WT and KO lines under normal conditions and 10% PEG treatment (representative images shown). Scale bar = 4 cm; C–D. Statistical analysis of plant height and root length of Nipponbare WT and OE lines under normal conditions and 10% PEG treatment (n = 16 plants); E–F. Statistical analysis of plant height and root length of Zhonghua 11 WT and KO lines under normal conditions and 10% PEG treatment (n = 16 plants); G–H. Phenotypic analysis and survival rate of Nipponbare WT and OE seedlings at the three-leaf stage before and after drought stress treatment (n = 9 biological replicates, 16 plants per replicate); I–J. Phenotypic analysis and survival rate of Zhonghua 11 and KO seedlings at the three-leaf stage before and after drought stress treatment (n = 9 biological replicates, 16 plants per replicate). Data are presented as mean ± SEM and were analyzed by one-way ANOVA with Tukey's multiple comparison test (H, J) and two-way ANOVA with Tukey's multiple comparison test (C–F), ** denotes an extremely significant difference (*P* < 0.01).

Given that *OsDUF1223* is specifically expressed at the seedling stage and induced by drought, we evaluated drought tolerance at multiple seedling developmental stages. First, 10% PEG was used to simulate mild drought stress for growth inhibition assays on young seedlings. Under normal conditions, no significant differences in plant height were observed among all lines. However, after 8 days of 10% PEG treatment, the plant height of overexpression lines was significantly lower than that of the wild type Nipponbare; in contrast, the knockout lines showed no obvious changes compared with ZH11. For root traits, under normal conditions, overexpression lines exhibited significantly longer roots than the wild type; conversely, the root length of the knockout lines was significantly shorter than that of ZH11. These differences in root length disappeared under mild drought treatment (Fig 2A–2F), indicating that overexpression lines suffered more severe growth inhibition under drought stress, whereas knockout lines displayed a growth advantage.

Furthermore, extreme drought treatments were performed on three-leaf and four-leaf stage seedlings in pot experiments (pre-experiment confirmed the optimal stress duration for different lines). Three-leaf stage pot assays showed that overexpression lines displayed more severe wilting under drought stress, with survival rates of 29.9%, 33.4%, and 41.7% after rewatering, which were significantly lower than the 68.8% of the Nipponbare. In contrast, compared with ZH11, the knockout lines showed milder wilting, with survival rates of 56.2% and 64.8%, which were significantly higher than that of their wild type (Fig 2G–2J). Phenotypes in four-leaf stage pot experiments were consistent with those at the three-leaf stage (S3 Fig). Collectively, overexpression of *OsDUF1223* reduced drought tolerance in rice seedlings, demonstrating that *OsDUF1223* acts as a negative regulator of drought stress responses at the rice seedling stage.

### Determination of drought tolerance indicators in *OsDUF1223* transgenic plants

**Fig 3:**
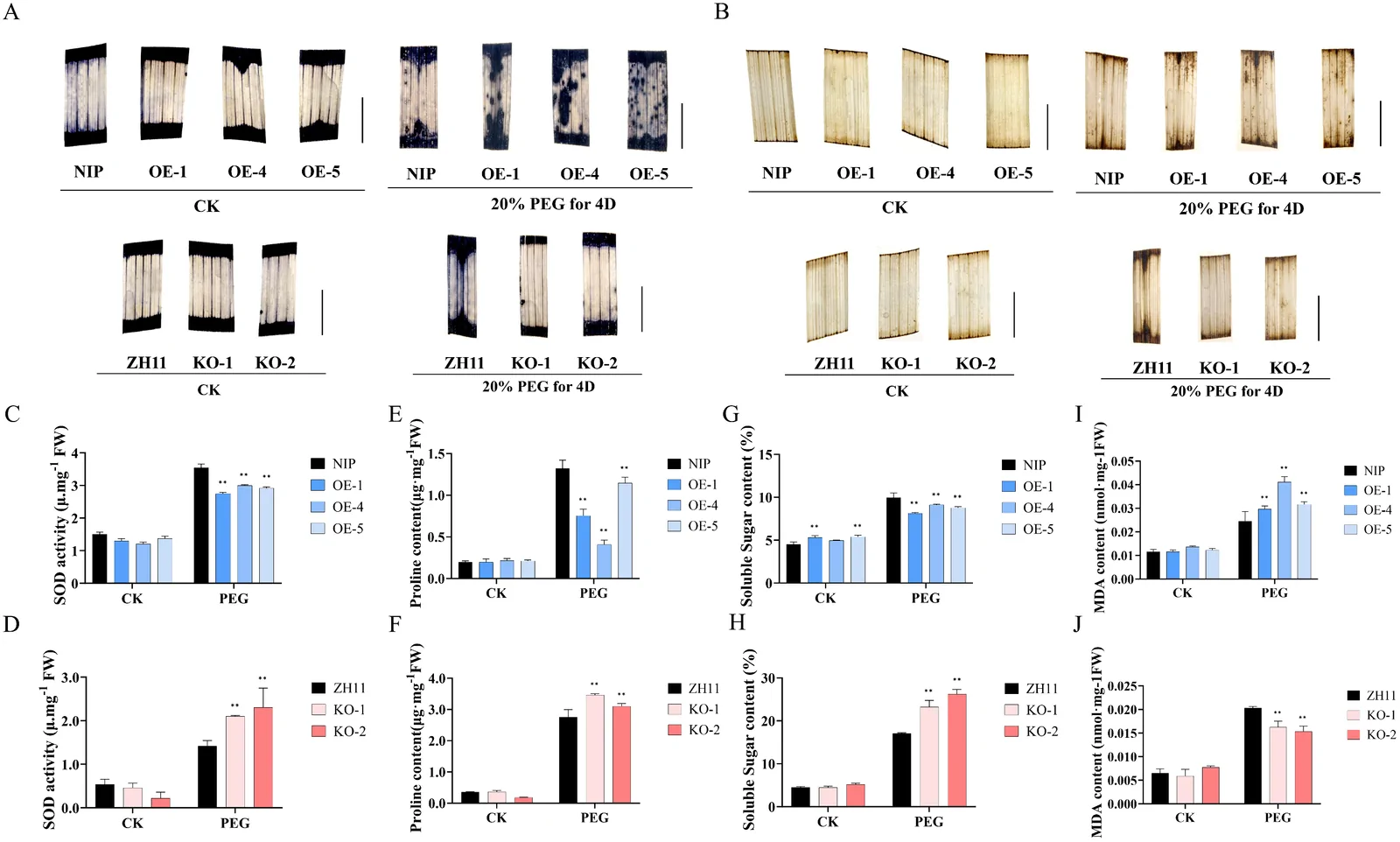
Physiological and biochemical index analysis of *OsDUF1223* transgenic seedlings. Note: A. NBT staining of transgenic lines (n = 3 biological replicates; representative images shown). Scale bar = 1 cm; B. DAB staining of transgenic lines (n = 3 biological replicates; representative images shown). Scale bar = 1 cm; C–D. SOD activity in Nipponbare WT/OE and Zhonghua 11 WT/KO lines (n = 3 technical replicates); E–F. Proline content in Nipponbare WT/OE and Zhonghua 11 WT/KO lines (n = 3 technical replicates); G–H. Soluble sugar content in Nipponbare WT/OE and Zhonghua 11 WT/KO lines (n = 3 technical replicates); I–J. MDA content in Nipponbare WT/OE and Zhonghua 11 WT/KO lines (n = 3 technical replicates). Data are presented as mean ± SEM and were analyzed by two-way ANOVA with Tukey’s multiple comparison test (C–J).. ** indicates a highly significant difference (*P* < 0.01).

To further clarify the regulatory mechanism of *OsDUF1223* on rice drought tolerance, this study used 20% PEG (pre-experiment confirmed for simulating severe drought) to systematically detect key physiological indicators in the leaves of transgenic lines, including reactive oxygen species (ROS) accumulation, antioxidant enzyme activity, osmotic adjustment substance content, and membrane lipid peroxidation level. First, to address drought-induced oxidative stress, DAB and NBT in situ staining were used to detect the accumulation of H_2_O_2_ and O_2_^-^ in leaves. The results showed no significant difference in ROS accumulation among all lines under normal conditions; however, after drought treatment, the DAB and NBT staining signals in the leaves of overexpression lines in the Nipponbare background were significantly enhanced, indicating explosive ROS accumulation, in contrast, the staining signals in knockout lines in the ZH11 background were significantly weakened (Fig 3A–3B). To objectively evaluate the staining results, we further performed semi-quantitative analysis of the DAB and NBT staining signals (S4 Fig). The results showed that after drought treatment, the relative accumulation levels of H_2_O_2_ and O_2_^-^ in the overexpression lines were significantly higher than those in Nipponbare; conversely, the knockout lines in the ZH11 background exhibited significantly lower levels than ZH11, which was fully consistent with the staining phenotypes. Together, these qualitative and quantitative results indicate that *OsDUF1223* may negatively regulate the plant's ROS scavenging capacity.

Under drought stress, plants maintain oxidative homeostasis by increasing the activity of ROS-scavenging enzymes and accumulate osmotic adjustment substances to maintain cell turgor, thereby synergistically alleviating oxidative damage. To further confirm that *OsDUF1223* negatively regulates ROS scavenging capacity, the activity of superoxide dismutase (SOD) activity was determined in this study. The results showed no significant difference in SOD activity among all lines under normal conditions; after drought treatment, the SOD activity of overexpression lines was significantly lower than that of Nipponbare; in contrast, under the ZH11 background, the SOD activity of the knockout lines was significantly higher than that of ZH11 (Fig 3C–3D). Meanwhile, the contents of major osmotic adjustment substances (proline and soluble sugars) were measured. The results showed no significant difference in proline content between transgenic lines and the wild type under normal conditions. Notably, under normal conditions, the soluble sugar content in some overexpression lines was significantly higher than that in Nipponbare; in contrast, under the ZH11 background, no significant difference was observed in the knockout lines. After drought treatment, the contents of proline and soluble sugars in overexpression lines were significantly lower than those Nipponbare; conversely, under the ZH11 background, these contents in the knockout lines were significantly higher than those in ZH11 (Fig 3E–3H). Disruption of ROS homeostasis causes cell membrane damage, which in turn increases malondialdehyde (MDA) content. To evaluate the degree of cell membrane damage, MDA content of each line was detected before and after drought treatment. The results showed no significant difference in MDA content among all lines under normal conditions. After drought treatment, the MDA content of overexpression lines was significantly higher than that of the Nipponbare, indicating more severe cell membrane damage; in contrast, under the ZH11 background, the MDA content of the knockout lines was significantly lower than that of ZH11 (Fig 3I–3J).

In summary, under drought stress, overexpression of *OsDUF1223* in rice seedlings is accompanied by reduced ROS scavenging capacity, decreased antioxidant enzyme activity, insufficient accumulation of osmotic adjustment substances, and aggravated membrane lipid peroxidation, while knockout lines in the ZH11 background show the opposite trends. These changes in physiological and biochemical indicators suggest that the expression level of *OsDUF1223* is negatively correlated with drought tolerance in rice seedlings. However, its regulatory role in drought tolerance still requires further validation through functional complementation and genetic interaction experiments.

### *OsDUF1223* affects the water loss rate of rice by regulating the stomatal morphological characteristics of rice leaves

**Fig 4:**
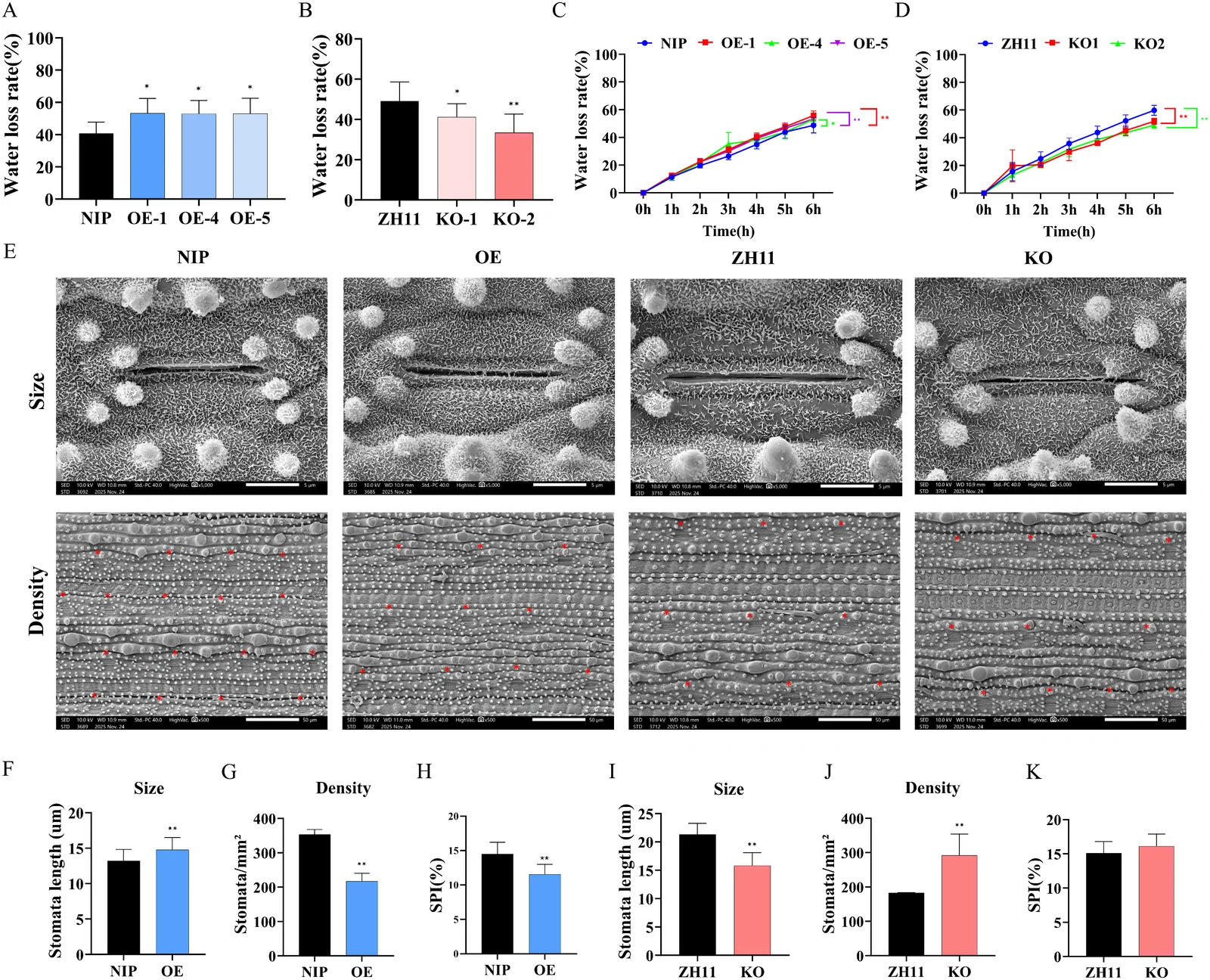
Water loss rate and stomatal morphology of *OsDUF1223* transgenic seedlings under drought stress. Note: Water loss rate of transgenic lines under drought stress: A. Nipponbare WT and OE lines (n = 9 plants); B. Zhonghua 11 WT and KO lines (n = 9 plants); Water loss rate of detached leaves of transgenic lines: C. Nipponbare WT and OE lines (n = 9 plants); D. Zhonghua 11 WT and KO lines (n = 9 plants); E. Leaf stomatal morphological characteristics of WT and transgenic seedlings after 8 h of drought stress (5 fields of view were selected from the same plant, representative images shown), red * indicates stomatal position. Scale bar = 5 μm; F, I: Stomatal aperture size statistics of OE plants in the Nipponbare background and KO plants in the Zhonghua 11 background (n = 20 stomata); G, J: Stomatal density statistics of OE plants in the Nipponbare background and KO plants in the Zhonghua 11 background (n = 5 fields of view); H, K: Stomatal pore index (SPI) statistics of OE plants in the Nipponbare background and KO plants in the Zhonghua 11 background (n = 20 stomata). Data are presented as mean  ±  SEM and were analyzed by unpaired, two-tailed Student’s t-test (F-K), one-way ANOVA with Tukey’s multiple comparison test (A-B) and two-way ANOVA with Tukey’s multiple comparison test (C-D), ** indicates an extremely significant difference (*P* < 0.01).

To explore the physiological basis for the differences in drought-induced wilting among transgenic lines in pot experiments, the leaf water loss rate of each line was measured. The results showed that under 20% PEG-induced severe drought stress, the water loss rate of overexpression lines was significantly higher than that of Nipponbare; in contrast, under the ZH11 background, the water loss rate of the knockout lines was significantly lower than that of the wild type (Fig 4A–4B). Under normal conditions, dynamic detection of water loss rate in detached leaves within 6 hours showed the same trend (Fig 4C–4D). To clarify the underlying mechanism, scanning electron microscopy was used to observe the leaf stomatal morphology after 8 hours of drought treatment. Results showed that, compared with the wild-type Nipponbare, overexpression lines exhibited significantly longer guard cells and significantly lower stomatal density (Fig 4F–4G). In contrast, knockout lines displayed significantly shorter guard cells and significantly higher stomatal density compared with the wild-type ZH11 (Fig 4I–4J). The stomatal pore-area index (SPI) was calculated as an integrated measure of stomatal morphology (Fig 4H, 4K). In overexpression lines, the reduction in stomatal density outweighed the effect of increased stomatal size, resulting in a significantly lower SPI value relative to the wild-type Nipponbare. In knockout lines, the SPI value showed no significant difference from that of the wild-type ZH11.

### Transcriptome analysis of *OsDUF1223* overexpression lines in response to drought stress

To explore the molecular mechanism, RNA-seq analysis was performed on Nipponbare wild-type (WT) and overexpression (OE) lines. Differentially expressed genes (DEGs) were identified using a threshold of |log_2_(fold change)| ≥ 1 and FDR < 0.05.. The results showed that, prior to treatment, compared with the WT, the OE lines exhibited 132 upregulated genes and 196 downregulated genes (S5A Fig). GO and KEGG enrichment analyses demonstrated that the upregulated DEGs were significantly enriched in the “nitrogen metabolism” and “fatty acid elongation” pathways (S5B–S5C Fig), whereas the downregulated DEGs were significantly enriched in the “plant hormone signal transduction,” “MAPK signaling pathway,” and “benzoxazinoid biosynthesis” pathways (S5D–S5E Fig).

**Fig 5:**
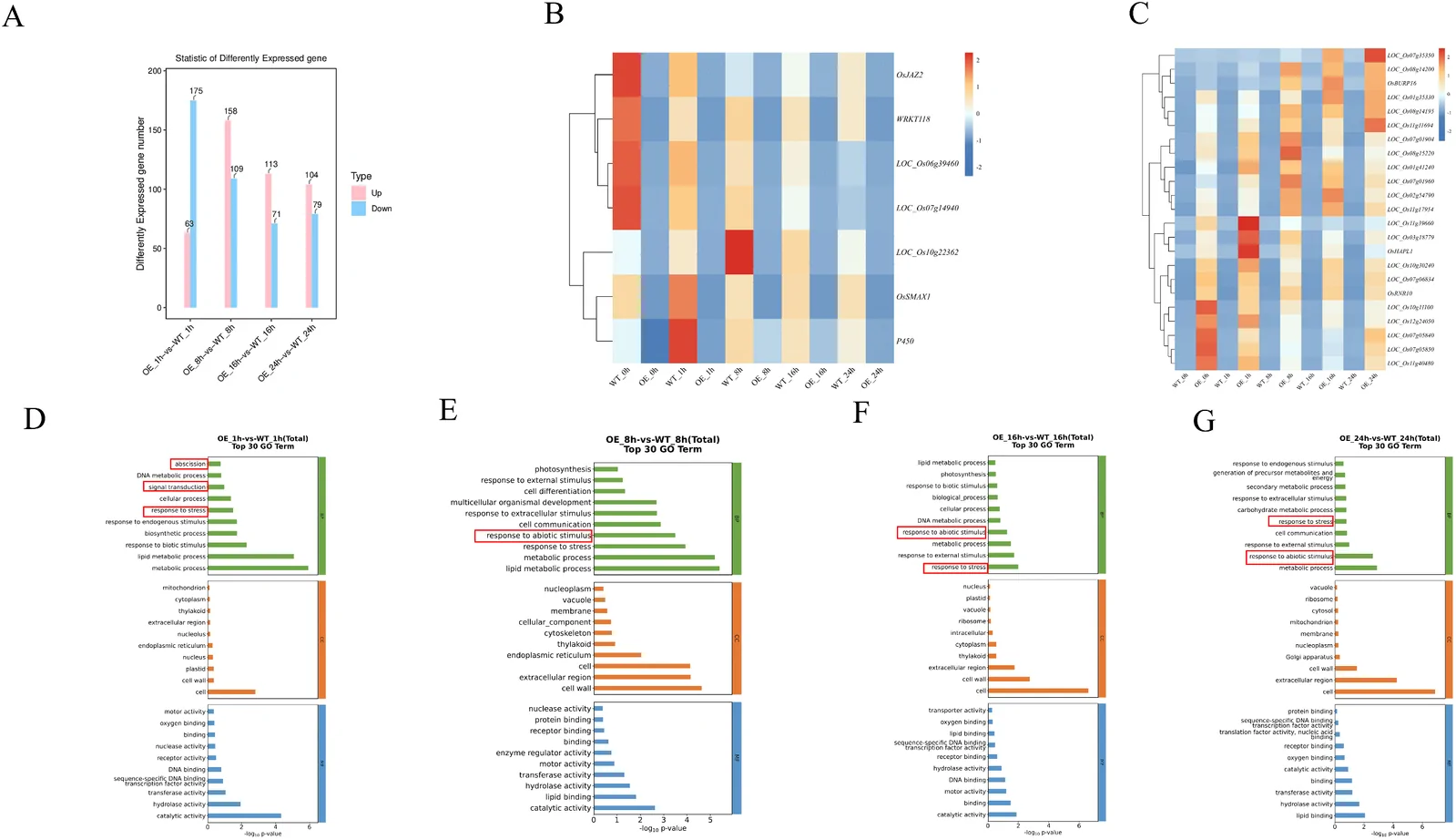
Transcriptome Analysis of *OsDUF1223* OE Plants. Note: A. Number of DEGs under different stress treatment durations; B. Overlapping downregulated DEGs commonly expressed across time points; C. Overlapping upregulated DEGs commonly expressed across time points; D–G. GO enrichment analysis bar charts under different stress treatment durations, with red boxes marking “abscission”, “signal transduction”, “response to abiotic stimulus”, and “response to stress”.

To further dissect the dynamic transcriptional changes induced by drought stress, we compared the DEG profiles of OE lines at 1, 8, 16, and 24 h of stress with those at 0 h. The number of upregulated DEGs at these time points was 63, 158, 113, and 104, respectively, while the number of downregulated DEGs was 175, 109, 71, and 79, respectively (Fig 5A). Subsequent overlapping analysis identified 30 common DEGs, including 7 downregulated genes (Fig 5B) and 23 upregulated genes (Fig 5C). The downregulated genes mainly included the JA signal negative regulator *OsJAZ2*, the abiotic stress transcription factor *OsWRKY118*, the strigolactone synthesis negative regulator *OsSMAX1*, as well as cytochrome P450 and transposon protein-encoding genes. The upregulated genes included the drought-sensitive gene *OsBURP16* and the root development/nitrogen response regulator *OsRNR10*.

**Fig 6:**
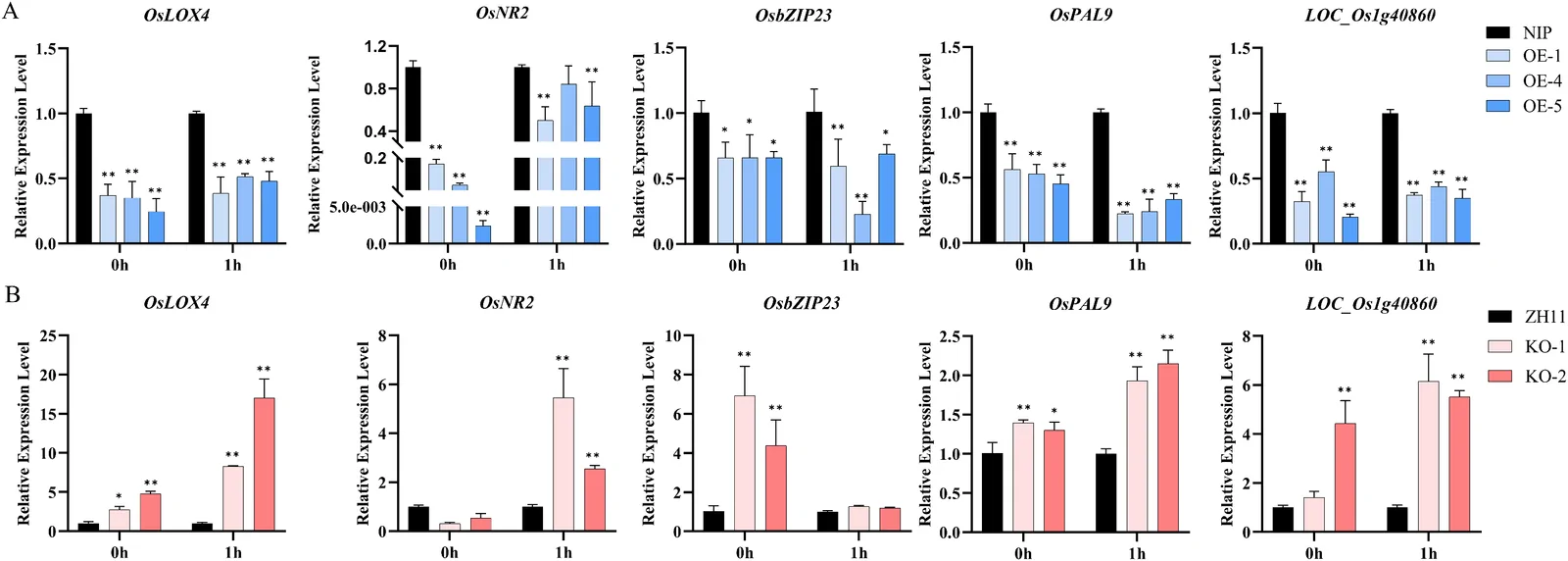
RT-qPCR validation of key stress-responsive genes in *OsDUF1223* transgenic lines under drought stress. Note: Relative transcript levels of genes OsLOX4, OsNR2, OsbZIP23, OsPAL9, and LOC_Os1g40860 before and after drought stress for 1 h in each line: A. Nipponbare WT and OE lines (n = 3, technical replicates); B. Zhonghua 11 WT and KO lines (n = 3, technical replicates). Data are presented as mean ± SEM and were analyzed by two-way ANOVA with Tukey’s multiple comparison test (H-I). * denotes a significant difference (0.01 < P < 0.05), ** denotes an extremely significant difference (P < 0.01). Because of this split, the numbering of subsequent figures needs to be shifted, and the corresponding figure citations throughout the text and figure legends should be updated accordingly. We would appreciate your assistance in making these changes.

GO enrichment analysis revealed that DEGs at all time points were significantly enriched in abiotic stimulus response and stress response pathways (Fig 5D–5G). We selected a set of key stress-responsive genes (*OsLOX4*, *OsNR2*, *OsbZIP23*, *OsPAL9*, *LOC_Os1g40860*) for RT-qPCR validation across the transgenic lines. The results showed that the expression trends in the OE lines under the Nipponbare background were consistent with the RNA-seq data, whereas the KO lines under the ZH11 background exhibited opposite trends compared to the RNA-seq data (Fig 6A, B). KEGG analysis further indicated that DEGs at 1 h of drought stress were enriched in pathways such as plant hormone signal transduction, betalain biosynthesis, and benzoxazinoid biosynthesis; those at 8 h were significantly enriched in cutin, suberin and wax biosynthesis, fatty acid elongation, and nitrogen metabolism pathways; and those at 16 h and 24 h were both significantly enriched in the nitrogen metabolism pathway (S6A–S6D Fig). Collectively, overexpression of *OsDUF1223* modulates the expression of genes associated with stress response, hormone signal transduction, and nitrogen metabolism. It should be noted that the transcriptomic analysis in this study was conducted exclusively in the Nipponbare overexpression (OE) background. The transcriptome data of knockout (KO) lines in the Zhonghua 11 background have not yet been presented. Therefore, the following descriptions of molecular responses in KO lines are based solely on RT-qPCR validation and have not been systematically confirmed at the transcriptomic level.

### *OsDUF1223* modulates the expression of genes related to hormone signaling pathways and affects the homeostasis of endogenous hormones

**Fig 7:**
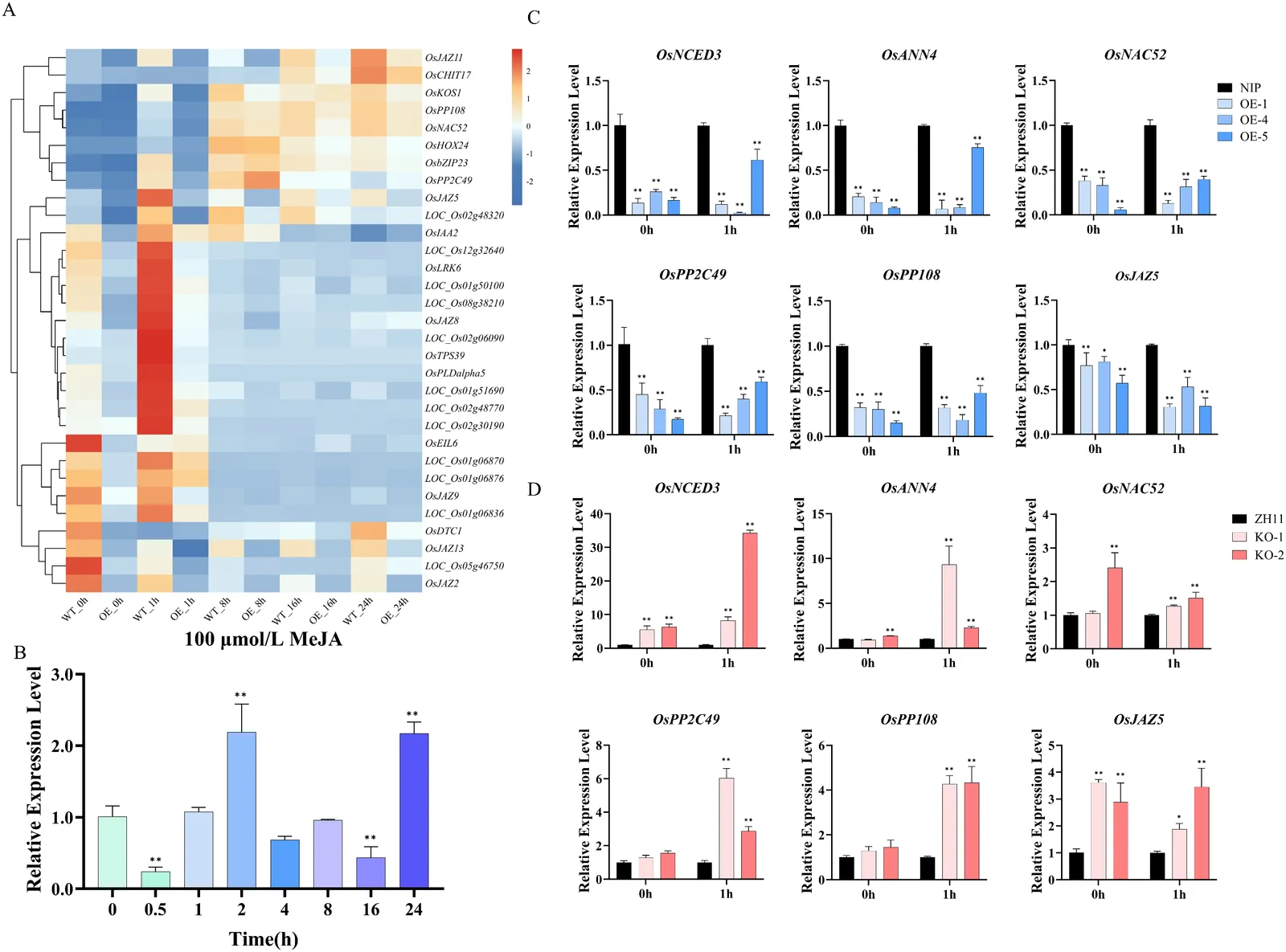
Expression and validation of hormone signaling pathway-related genes in *OsDUF1223* transgenic lines. Note: A. Heatmap of DEGs significantly enriched in signaling pathways (GO) and hormone signaling pathways (KEGG); B. Relative transcript levels of the *OsDUF1223* gene after 24 h of treatment with 100 μmol/L MeJA (n = 3, technical replicates). Relative transcript levels of genes *OsNCED3*, *OsANN4*, *OsNAC52*, *OsPP2C49*, *OsPP108* and *OsJAZ5* in each line before and after drought stress treatment for 1 h: C. Nipponbare WT and OE lines (n = 3, technical replicates); D. Zhonghua 11 WT and KO lines (n = 3, technical replicates). Data are presented as mean  ±  SEM and were analyzed by one-way ANOVA with Tukey’s multiple comparison test (B) and two-way ANOVA with Tukey’s multiple comparison test (C-D). * denotes a significant difference (0.01 < *P* < 0.05), ** denotes an extremely significant difference (*P* < 0.01).

To investigate the regulatory role of *OsDUF1223* in hormone signal transduction, we analyzed the differentially expressed genes (DEGs) enriched in signal transduction and hormone signal transduction pathways in OE lines at 0 h and 1 h after drought stress treatment. Heatmap analysis (Fig 7A) showed that these genes included key regulators of multiple hormone signaling pathways, such as *OsNCED3* (encoding an ABA biosynthesis enzyme), *OsANN4* (encoding a Ca² ⁺ -binding protein that mediates ABA-induced antioxidant responses), and *OsNAC52* (encoding a transcription factor involved in ABA responses). The results of RT-qPCR validation were consistent with the expression trends observed in the transcriptome data (Fig 7C–6D), indicating that *OsDUF1223* modulates the expression of hormone signal transduction-related genes in rice seedlings. Based on these results, we hypothesized that the JA and ABA signaling pathways are the major hormone signal transduction pathways regulated by *OsDUF1223*. To further explore the response characteristics of *OsDUF1223* to JA signals, wild-type three-leaf-stage seedlings were treated with 100 μmol/L MeJA. RT-qPCR analysis revealed that the relative transcript level of *OsDUF1223* was significantly down-regulated at 0.5 h and 16 h, and significantly up-regulated at 2 h and 24 h after treatment (Fig 7B), demonstrating that the expression of *OsDUF1223* is induced by MeJA and further confirming its involvement in the regulation of hormone signal transduction in rice. To investigate the regulatory role of *OsDUF1223* in endogenous hormone homeostasis, we measured the contents of abscisic acid (ABA), jasmonic acid (JA), and salicylic acid (SA) in transgenic materials. Under both normal and drought conditions, the endogenous hormone levels in the knockout and overexpression lines differed from those in the wild type, with the most pronounced difference observed in ABA content. After drought treatment, the ABA content in the knockout lines was significantly higher than that in ZH11, whereas under both normal and drought conditions, the ABA content in the overexpression lines was lower than that in Nipponbare (S2 and S3 Tables). These results suggest that *OsDUF1223* may participate in the regulation of endogenous hormone homeostasis by modulating ABA biosynthesis and metabolism, thereby influencing drought tolerance in rice.

### *OsDUF1223* positively regulates the ABA signaling pathway, and ABA acts as an essential downstream regulatory signal for it

**Fig 8:**
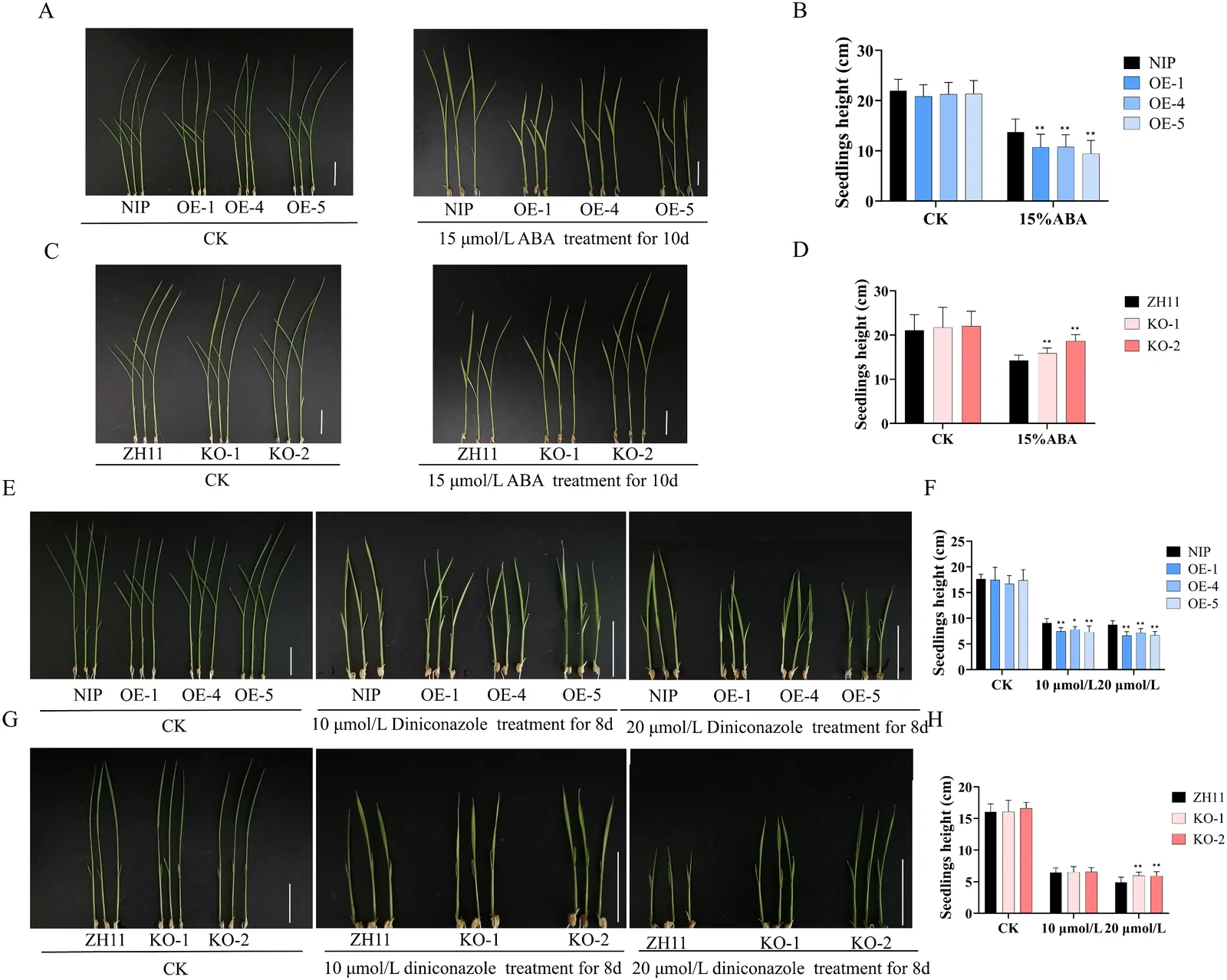
Growth performance of *OsDUF1223* transgenic seedlings under ABA treatment conditions. Note: Plant height phenotypes and statistical analysis of transgenic lines under normal conditions and 15 μmol/L ABA treatment: A–B. Nipponbare WT and OE lines (B: n = 16 plants); C–D. Zhonghua 11 WT and KO lines (D: n = 16 plants). Plant height phenotypes and statistical analysis of transgenic lines under normal conditions, 10 μmol/L diniconazole, and 20 μmol/L diniconazole treatment: E–F. Nipponbare WT and OE lines (F: n = 16 plants); G–H. Zhonghua 11 WT and KO lines (H: n = 16 plants). Data are presented as mean  ±  SEM and were analyzed by two-way ANOVA with Tukey’s multiple comparison test (B, D, F, H). * denotes a significant difference (0.01 < *P* < 0.05), ** denotes an extremely significant difference (*P* < 0.01).

Further investigations into the ABA pathway revealed that after treating two-day-old seedlings with 15 μmol/L ABA for 10 days, the plant height of overexpression lines was significantly lower than that of Nipponbare; in contrast, under the ZH11 background, the plant height of the knockout lines was significantly higher than that of ZH11 (Fig 8A–7D). When two-day-old seedlings were treated with different concentrations of diniconazole (an ABA synthesis inhibitor), the plant height of the overexpression lines was lower than that of Nipponbare at both 10 μmol/L and 20 μmol/L concentrations; in contrast, under the ZH11 background, the knockout lines showed significantly greater plant height than ZH11 only under 20 μmol/L diniconazole treatment (Fig 8E–7H). These results demonstrated that *OsDUF1223* positively regulates the ABA signaling pathway. Complementation assays showed that no significant difference in water loss rate was observed among all lines after combined treatment with 20% PEG and 50 μmol/L ABA for 2 days (S7A–S7B Fig). Scanning electron microscopy observations revealed that the combined treatment induced stomatal closure in the overexpression lines (S7C Fig). These findings indicated that exogenous ABA could eliminate the difference in water loss rate between transgenic lines and the wild type, confirming that ABA serves as an essential downstream signal for *OsDUF1223* to regulate drought tolerance.

### *OsDUF1223* affects the drought tolerance of rice by participating in the regulation of root architecture mediated by nitrogen

**Fig 9:**
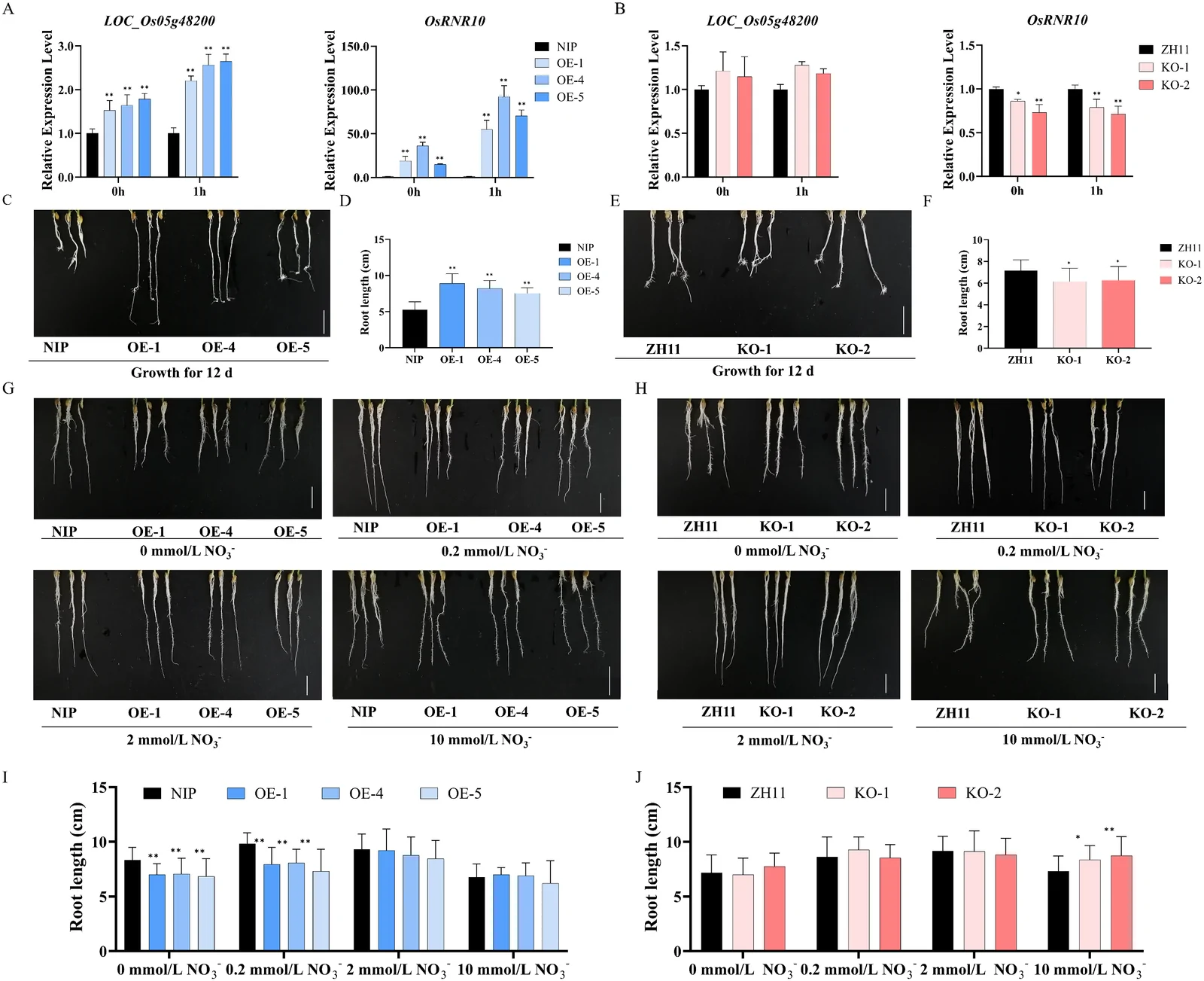
Nitrogen response of *OsDUF1223* transgenic plants. Note: Relative transcript levels of genes *LOC_Os05g48200* and *OsRNR10* in transgenic lines before and after drought stress treatment for 1 h: A. Nipponbare WT and OE lines (n = 3, technical replicates); B. Zhonghua 11 WT and KO lines (n = 3, technical replicates); C–D. Root system architecture phenotypes and root length statistics of Nipponbare WT and OE lines (D: n = 16 plants). Scale bar = 2 cm; E–F. Root system architecture phenotypes and root length statistics of Zhonghua 11 WT and KO lines (F: n = 16 plants). Scale bar = 2 cm; G–H. Root growth phenotypes of transgenic lines under different NO_3_^-^ concentrations. Scale bar = 2 cm; I–J. Statistical analysis of root length of transgenic lines under different NO_3_^-^ concentrations (n = 24 plants). Data are presented as mean  ±  SEM and were analyzed by one-way ANOVA with Tukey’s multiple comparison test (D, F) and two-way ANOVA with Tukey’s multiple comparison test (A-B, I-J). * denotes a significant difference (0.01 < *P* < 0.05), ** denotes an extremely significant difference (*P* < 0.01).

To verify the transcriptome results, this study selected nitrogen metabolism pathway-related DEGs, such as *LOC_Os05g48200* (encoding glutamine synthetase) and *OsRNR10* (regulating rice root architecture and nitrogen response), and detected their relative transcription levels in each transgenic line using RT-qPCR (Fig 8A–8B). The results were consistent with the trend of transcriptome data, suggesting that *OsDUF1223* may be involved in the nitrogen response. Combined with the literature report that nitrate can promote plant root elongation [45], this study further explored the effect of *OsDUF1223* on the root growth of rice seedlings. After 2-day-old seedlings were cultured in modified nitrogen-free Hoagland's medium containing 2 mmol/L NO_3_^-^ for 12 days, the root lengths of *OsDUF1223* overexpression lines were 8.91 cm, 8.18 cm, and 7.54 cm, respectively, which were significantly longer than the 5.27 cm of Nipponbare. In contrast, under the ZH11 background, the root lengths of the knockout lines were 6.16 cm and 6.27 cm, respectively, which were significantly shorter than the 7.16 cm of ZH11 (Fig 9C–8F). These results indicate that *OsDUF1223* positively regulates the root elongation of rice seedlings.

Four concentration gradients of NO_3_^-^ (0, 0.2, 2, and 10 mmol/L) were set (with 1 mmol/L glutamine as the background nitrogen source) to simulate different nitrogen conditions. After 8 days of culture, it was found that the root length of overexpression lines was significantly shorter than that of Nipponbare under nitrogen-free and low-nitrogen conditions, while no significant differences were observed under normal-nitrogen and high-nitrogen conditions. In contrast, under the ZH11 background, the knockout lines exhibited significantly longer roots than ZH11 under nitrogen-free and low-nitrogen conditions, while no significant differences were observed under normal-nitrogen and high-nitrogen conditions either (Fig 9G–9J). Moreover, under 2 mmol/L NO_3_^-^ and 10 mmol/L NO_3_^-^ treatment, there was no significant difference in root length between each transgenic line and the wild type. This may be because glutamine, as a direct product of nitrogen assimilation, can be preferentially utilized by plants, thereby reducing their dependency on exogenous nitrate [46,47]. The above results indicate that the regulation of root growth by *OsDUF1223* is nitrate concentration-dependent and affected by the organic nitrogen background, suggesting that it may participate in the regulation of rice drought tolerance by regulating nitrogen-mediated root architecture.

## Discussion

This study demonstrates that the expression of *OsDUF1223* is significantly induced by drought stress and shows spatiotemporal specificity at the rice seedling stage. Phenotypic analysis revealed that *OsDUF1223*-knockout lines exhibited milder growth inhibition, less wilting, and higher survival rates after rewatering under drought stress relative to their Zhonghua 11 controls, whereas overexpression lines in the Nipponbare background showed lower survival rates relative to their Nipponbare controls. These results confirm that *OsDUF1223* functions as a negative regulator of drought tolerance in rice seedlings. Because the knockout and overexpression lines were generated in two different japonica backgrounds (Zhonghua 11 and Nipponbare, respectively), all comparisons were performed exclusively between each transgenic line and its corresponding wild-type control. Cross-background differences were interpreted only as qualitative trends, not as quantitative comparisons. The two wild-type cultivars exhibited inherent background-dependent differences in basal drought tolerance, with Zhonghua 11 being more tolerant than Nipponbare. Accordingly, drought treatments were applied for 3 days to the Nipponbare-based lines and for 4 days to the Zhonghua 11-based lines—durations at which each background reached a comparable moderate stress intensity—so that genotype effects could be evaluated under similar stress levels. Notably, the direction of the *OsDUF1223* effect was consistent across both backgrounds: loss of function enhanced survival, whereas overexpression reduced survival, relative to the respective control. This consistency indicates that the regulatory function of *OsDUF1223* is reproducible regardless of genetic background. Physiological and biochemical assays further indicated that knockout lines possessed stronger ROS-scavenging ability, greater osmolyte accumulation, and more stable membrane systems under drought stress, which underpin their enhanced drought resistance [44]. The dynamic changes in soluble sugar and proline content reveal a key physiological pattern: although overexpression lines exhibited higher soluble sugar levels prior to treatment relative to their wild-type controls, these levels declined sharply after drought treatment— suggesting excessive consumption of osmoprotective substances or impaired homeostatic maintenance capacity [48]. In contrast, knockout lines in the ZH11 background maintained relatively high levels of both soluble sugars and proline during the later stages of treatment, consistent with their enhanced drought tolerance. These results indicate that *OsDUF1223* loss of function enhances drought tolerance by reducing excessive mobilization of osmolytes under drought conditions, whereas overexpression promotes excessive mobilization of carbon and nitrogen metabolites, leading to metabolic homeostasis imbalance and drought sensitivity [49].

Stomatal observations revealed that under drought conditions, overexpression lines exhibited larger stomata but a significantly lower stomatal pore-area index (SPI) compared to Nipponbare, due to a more pronounced reduction in stomatal density. However, leaf water loss rate assays showed that overexpression lines had a significantly higher water loss rate than Nipponbare. This apparent discrepancy between low SPI and high water loss suggests that differences in water loss are not solely determined by static stomatal morphology, but likely reflect alterations in stomatal movement regulation. Previous studies have established that guard cell size is closely linked to closure kinetics: larger guard cells require greater ion flux changes to complete turgor regulation, resulting in significantly slower response rates compared to smaller stomata [50,51]. Based on these findings, we propose that the elevated water loss rate in overexpression lines may stem from delayed or incomplete stomatal closure under drought stress due to larger guard cells, leading to persistently higher stomatal conductance. Future studies measuring stomatal conductance (g_s_) and stomatal opening/closing kinetics under drought conditions will be essential to further elucidate the specific molecular mechanisms by which *OsDUF1223* regulates stomatal movement and water use efficiency.

Transcriptome analysis provided direct molecular evidence for the involvement of *OsDUF1223* in ABA signal transduction. Under drought treatment at 0 h and 1 h, differentially expressed genes in the overexpression lines were significantly enriched in the “stress response” and “hormone signal transduction” pathways, encompassing multiple core ABA signaling components. The heatmap results revealed that the abiotic stress and ABA response factor *OsPP108*, the negative regulator of ABA signaling *OsPP2C49*, and the core positive regulator of ABA signaling *OsbZIP23* were all downregulated in the overexpression lines. PP2C family proteins are core negative regulators of the ABA signaling pathway; their reduced expression relieves the inhibition of downstream SnRK2 kinases, rendering the ABA signaling pathway more prone to activation [52]. Notably, *OsbZIP23* functions not only as a transcriptional activator of *OsPP2C49* but also as a positive regulator of ABA-responsive gene expression and NCED-dependent ABA biosynthesis [52,53]; its synchronous downregulation with *OsPP2C49* therefore reflects a cause-and-effect relationship within the same regulatory cascade rather than two independent signals. This coordinated reduction exerts opposing effects on ABA signaling: the loss of *OsPP2C49* sensitizes signal transduction, whereas the reduced *OsbZIP23* attenuates ABA biosynthesis and ABA-responsive gene activation. The net ABA phenotype thus depends on the balance between these two arms. Furthermore, altered expression of JAZ family proteins in the JA pathway implied a complex crosstalk between ABA and JA signals, which together shaped the *OsDUF1223*-mediated stress response network [54,55].

This study found that endogenous ABA content in *OsDUF1223* overexpression lines was significantly lower than that in the wild type. Despite this, the OE lines exhibited significantly higher sensitivity to exogenous ABA and stronger growth inhibition in response to the triazole-type P450 inhibitor diniconazole. Diniconazole has been shown to inhibit ABA 8’-hydroxylase (CYP707A), the key enzyme in ABA catabolism, thereby elevating endogenous ABA levels [56]. This differential response to the inhibitor versus exogenous ABA can be explained by their opposing effects on ABA catabolism: exogenously applied ABA is rapidly cleared by ABA-induced, CYP707A-mediated degradation, resulting in only moderate growth inhibition [57]. In contrast, diniconazole blocks this degradation pathway, allowing endogenous ABA to accumulate and exert sustained signaling in the hypersensitive OE lines. This suggests that *OsDUF1223* exerts a dual regulatory effect on the ABA system. First, within the bZIP23–*OsPP2C49* cascade, although the downregulation of *OsbZIP23* attenuates its positive regulatory function in activating ABA-responsive genes, the net phenotype is dominated by the reduction of the terminal negative regulator, *OsPP2C49*. Because PP2Cs function as gatekeepers that directly dephosphorylate and inactivate SnRK2s, their removal can sensitize the signaling pathway even when ABA ligand levels are moderately reduced. Furthermore, given that *OsbZIP23* activity is primarily controlled by SnRK2-mediated phosphorylation rather than by transcript abundance, its reduced mRNA level does not necessarily translate into reduced protein activity. The elevated SnRK2 activity resulting from *OsPP2C49* reduction may even enhance the phosphorylation status of the remaining *OsbZIP23* protein, further reinforcing the hypersensitive phenotype. Second, the downregulation of *OsbZIP23* leads to attenuated NCED-dependent ABA biosynthesis, maintaining endogenous ABA at a low steady-state level. This “low-content, high-sensitivity” regulatory model allows plants to maintain a stress-alert state without accumulating high concentrations of inhibitory hormones, thereby achieving a balance between growth and defense [58]. However, this model should be interpreted within a temporal context. Under well-watered conditions, the sensitized pathway primes stress alertness without a growth penalty. Conversely, upon drought onset, the limited endogenous ABA pool cannot mount a sufficiently rapid and strong ABA response, which accounts for the impaired drought tolerance of the OE lines. This interpretation is supported by exogenous ABA complementation experiments, which confirmed that ABA is an essential downstream signaling molecule for *OsDUF1223* in regulating drought tolerance. Under combined treatment with 20% PEG and 50 μmol/L ABA, the differences in water loss rate among all lines were eliminated; scanning electron microscopy observations also indicated that the combined treatment induced stomatal closure in the overexpression lines, demonstrating that exogenous ABA could rescue the drought tolerance defect caused by *OsDUF1223* overexpression. Furthermore, differentially expressed genes enriched in the “stress response” pathway included multiple ABA-dependent stress response factors: *OsNAC52* can transcriptionally activate ABA-inducible gene expression [59], and *OsANN4* can mediate ABA-induced calcium influx and regulate antioxidant enzyme activity [60]. These findings further support the role of *OsDUF1223* in regulating drought tolerance through the ABA pathway.

Our study demonstrates that *OsDUF1223* positively regulates root elongation in rice seedlings. Under normal conditions, overexpression (OE) lines in the Nipponbare background exhibited significantly longer roots, while knockout (KO) lines in the ZH11 background displayed markedly shorter roots. However, under mild drought stress, these differences diminished: OE lines showed greater root growth inhibition, whereas KO lines exhibited milder suppression, indicating that root plasticity is dynamically modulated by water status. The disappearance of root length differences under drought, while drought tolerance differences persisted, suggests that root architecture is not a direct causal pathway through which *OsDUF1223* regulates drought tolerance. Transcriptomic analyses provided key insights into the core mechanism. KEGG enrichment analysis revealed that, even at the 0 h time point, upregulated differentially expressed genes in the overexpression lines were significantly enriched in the “nitrogen metabolism” pathway, suggesting that *OsDUF1223* exerts a constitutive regulatory effect on nitrogen metabolism. Although the 0 h enrichment may partially reflect genotypic differences in homeostatic state— including potential effects of transgene insertion— the consistent and significant enrichment of this pathway at 8 h, 16 h, and 24 h under stress indicates that this regulation is both maintained and specifically engaged during drought stress. Therefore, we propose that the drought-responsive specificity of the nitrogen metabolism pathway is primarily supported by its dynamic transcriptional changes during the later stages of stress, rather than being invalidated by the 0 h enrichment alone. Taken together, *OsDUF1223* participates in the drought response by regulating nitrogen metabolism, and its function is not limited to root development.

Among the overlapping DEGs consistently upregulated across all time points, *OsRNR10* is known to inhibit NO_3_^-^ uptake and root development by modulating auxin accumulation [61]. Theoretically, *OsRNR10* upregulation should suppress root growth; however, OE lines exhibited elongated roots. This contradiction has not been mechanistically resolved in the present study; it may reflect functional plasticity of *OsRNR10* across different genetic backgrounds or stress conditions, or may suggest that *OsDUF1223* functionally antagonizes *OsRNR10* through yet-to-be-identified downstream factors. Resolving this contradiction will require further auxin phenotypic analyses and genetic interaction experiments between *OsRNR10* and *OsDUF1223*. Nitrate concentration gradient experiments demonstrated that the root phenotype effects of *OsDUF1223* are dependent on nitrogen availability: under nitrogen-free or low-nitrogen conditions, OE lines exhibited significantly shorter roots than wild-type plants, whereas no difference was observed under high-nitrogen conditions. Conversely, KO lines in the ZH11 background showed the opposite trend, suggesting that *OsDUF1223*-mediated root developmental regulation is closely linked to nitrogen signaling pathways. These findings reveal an association between nitrogen signaling and root development, and provide a reasonable explanation for the apparent contradiction between *OsRNR10* upregulation and root elongation in overexpression lines: the final root phenotype is not determined by a single gene, but rather reflects the integrated output of the nitrogen signaling network. Under nitrogen-sufficient conditions, the net balance between the auxin-mediated inhibitory effect of *OsRNR10* and the promoting effect of *OsDUF1223* on root growth may favor elongation. When nitrogen availability decreases, this balance shifts toward inhibition, consistent with the shorter roots observed in overexpression lines under nitrogen-free or low-nitrogen conditions. Definitive resolution of this balance will require auxin phenotypic analyses and genetic interaction experiments between *OsRNR10* and *OsDUF1223*.

Based on the above results, we propose the following working model: *OsDUF1223* serves as a potential multifunctional regulatory hub that may coordinate the drought response in rice through two synergistic mechanisms. It should be emphasized that this model is based predominantly on correlational evidence from phenotypic and transcriptomic analyses; it does not yet establish direct physical or regulatory interactions between *OsDUF1223* and ABA or nitrogen pathway components, and causal relationships remain to be experimentally validated. First, *OsDUF1223* is functionally associated with the ABA signaling pathway, and its regulation appears to modulate both the homeostasis and the transduction of ABA signals during drought stress. At the seedling stage, in the *OsDUF1223*-overexpressing lines, drought-induced *OsDUF1223* expression is accompanied by the downregulation of bZIP23 and its downstream target *OsPP2C49*. Within this cascade, the coordinated downregulation of *OsbZIP23* and *OsPP2C49* governs two dimensions of the ABA system: the reduction of *OsPP2C49* relieves the inhibition of SnRK2 kinases, sensitizing the signaling pathway even under low ABA levels; while the downregulation of *OsbZIP23* attenuates NCED-dependent ABA biosynthesis, maintaining endogenous ABA at a low steady-state level. Together, they establish a “low-content, high-sensitivity” state. In overexpression (OE) lines, the reduced endogenous ABA is insufficient to fully activate downstream drought responses under natural drought conditions, and exogenous ABA application can rescue this defect; conversely, knockout (KO) lines exhibit elevated endogenous ABA levels and enhanced overall drought tolerance, despite reduced signaling sensitivity. Second, *OsDUF1223* is associated with the nitrogen metabolism pathway in a nitrate concentration-dependent manner, thereby remodeling root architecture. Previous studies have shown that rational nitrogen supply under drought stress can enhance plant drought tolerance [62], and efficient root architecture is fundamental for plant water uptake [63]. Under specific nitrogen conditions, *OsDUF1223* reshapes root architecture by regulating key nitrogen metabolism genes. These pre-established root differences may affect overall water balance and stress adaptation when drought occurs. Notably, the disappearance of root differences under mild drought stress suggests that plants may dynamically remodel root architecture to adapt to environmental changes by coordinating the “nitrogen-water coupling” mechanism. This provides a new genetic target for understanding the regulatory mechanisms of nitrogen-water interactions, although the precise molecular mechanisms by which *OsDUF1223* perceives nitrogen signals and translates them into root developmental instructions remain to be further investigated. Based on these transcriptomic clues, we propose the following explicit, testable hypotheses. First, *OsDUF1223* may modulate ABA signaling sensitivity through direct or indirect protein–protein interactions with clade A PP2C family members (e.g., *OsPP2C49*) or SnRK2 kinases (OsSAPKs). This can be tested by yeast two-hybrid (Y2H), bimolecular fluorescence complementation (BiFC), co-immunoprecipitation (Co-IP), and in vitro phosphorylation assays. Second, *OsDUF1223* may influence nitrogen metabolism and root architecture through transcriptional regulation of key nitrogen-signaling components, such as OsNLP family transcription factors or nitrate transporter genes. This can be tested by DAP-seq/ChIP-seq, electrophoretic mobility shift assays (EMSA), and genetic interaction analyses with nitrogen-signaling mutants. Third, *OsDUF1223* may exert its effects at the ABA–nitrogen crosstalk node through shared downstream physiological outputs (e.g., osmotic adjustment and reactive oxygen species homeostasis), rather than through direct physical interactions with pathway components. This hypothesis is testable through epistasis analysis in double mutants.

This study provides evidence that *OsDUF1223* may be associated with drought tolerance in rice seedlings through its functional links to ABA signaling and nitrogen metabolism. Through this functional association with both pathways, we have preliminarily proposed a potential molecular framework underlying *OsDUF1223*-mediated drought tolerance in rice seedlings, although several limitations remain. First, transgenic materials were generated in different genetic backgrounds (Nipponbare for overexpression and ZH11 for knockout), which may interfere with direct phenotypic comparisons. Additionally, the current transcriptomic data only reflect transcriptional regulatory effects of *OsDUF1223* under the overexpression background and cannot fully exclude background-related confounding factors. Second, the direct interaction between *OsDUF1223* and core components of the ABA signaling pathway remains unclear; the crosstalk network between nitrogen and ABA signals, as well as the hormonal mechanisms regulating root plasticity, have yet to be resolved. Third, the current consistency among phenotypic, physiological, and gene expression data constitutes an indirect line of evidence supporting the negative regulation of drought tolerance by *OsDUF1223*, but has not yet reached the level of rigorous genetic causal validation—the possibility of CRISPR off-target effects in the CRISPR-generated KO lines, as well as whether *OsDUF1223* mediates its drought phenotype through known ABA signaling components such as *OsPP2C49* or *OsbZIP23*, both require further confirmation. Fourth, this study focused solely on the seedling stage; the functions of *OsDUF1223* at the adult stage and under field drought conditions, as well as its potential impacts on agronomic traits, remain to be verified.

## Materials and methods

### Bioinformatic analysis

Multi-omics data of rice seedlings under drought stress and drought-ABA combined stress were retrieved from the Genome Sequence Archive (GSA) of the National Genomics Data Center, China National Center for Bioinformation (https://ngdc.cncb.ac.cn/gsa-human/). The gene sequence of *OsDUF1223* (*LOC_Os07g37310*) and its 1.5 kb upstream promoter sequence were obtained from the Rice Genome Annotation Project (RGAP) database (https://rice.uga.edu/index.shtml) and the National Center for Biotechnology Information (NCBI) database, respectively.

DUF1223 protein sequences were retrieved from the Ensembl Plants database (https://plants.ensembl.org/index.html), including those from rice, *Arabidopsis thaliana* (L.) Heynh., wheat (*Triticum aestivum* L.), barley (*Hordeum vulgare* L.), cucumber (*Cucumis sativus* L.), soybean (*Glycine max* (L.) Merr.), tomato (*Solanum lycopersicum* L.), *Populus trichocarpa* Torr. & Gray, and maize (*Zea mays* L.). Motif analysis was performed using the MEME Suite (https://meme-suite.org/meme/), and image processing was conducted with TBtools.

### Plant material and growth conditions

The overexpression (OE) lines used in this study were developed with Oryza sativa L. subsp. japonica cv. Nipponbare as the genetic background and provided by Wuhan Tianwen Biotechnology Co., Ltd. The knockout (KO) lines were generated with Oryza sativa subsp. japonica cv. Zhonghua 11 as the genetic background and provided by Baige Biotechnology Co., Ltd. (Note: the two varieties are both conventional japonica rice, and the follow-up will supplement the same background transgenic lines for verification).

Seeds were soaked in 3% H_2_O_2_ solution for 30 min for disinfection, followed by rinsing with sterile water and germination in the dark. Rice seedlings were then cultured in a growth chamber with modified Yosida nutrient solution [64], and grown to the three-leaf stage under the conditions of 16 h light (28°C) and 8 h dark (25°C). The nutrient solution for cultivation and stress treatment was renewed every 3 days to ensure nutrient supply.

### RNA extraction and real-time qPCR analysis

Two-week-old wild-type seedlings were treated with 20% PEG (drought simulation), 50 μmol/L ABA, and 100 μmol/L methyl jasmonate (MeJA) for 24 h, respectively. Shoot samples were collected at 0, 0.5, 1, 2, 4, 8, 16, and 24 h after treatment, with 3 biological replicates per time point. Total RNA was extracted using RNA TRIzol (Vazyme) following the manufacturer’s instructions and reverse-transcribed into cDNA using the HiFiScript All-in-one RT Master Mix kit. RT-qPCR was performed with SYBR Green PCR Master Mix (ABclonal) on a real-time PCR instrument, using Ubiquitin 5 (Os01g0328400) as the internal reference gene. All primer sequences are listed in S4 Table.

### Histochemical detection of GUS activity

The 1.5 kb promoter sequence upstream of the start codon of *OsDUF1223* was cloned into the pCAMBIA1305 vector to generate the recombinant *proOsDUF1223*::GUS construct. Transgenic plants were obtained via *Agrobacterium*-mediated transformation [65] and propagated in the experimental field. Mature leaves, anthers, glumes, 5-day-old seedlings, and stems and leaves at the three-leaf stage were collected for GUS staining. Five-day-old seedlings were treated with 20% PEG and 50 μmol/L ABA for 0.5 h, followed by GUS staining in the dark for 12 h [66]. The samples were then destained with a 95% ethanol gradient and observed under a Zeiss stereomicroscope. The detection primers are listed in S5 Table.

### Subcellular localization of *OsDUF1223* protein

The full-length coding sequence (CDS) of *OsDUF1223* without the stop codon was cloned into the pCAMBIA1300-35S-GFP vector to generate the recombinant 35S:*OsDUF1223*-GFP construct. Meanwhile, the coding sequence of the *Arabidopsis thaliana* plasma membrane aquaporin gene *AtPIP2A* was cloned into the pK7m34GW vector to generate the *AtPIP2A*-mCherry fusion construct [67], which served as a plasma membrane marker. The constructs were then transformed into *Agrobacterium tumefaciens* strain EHA105 and stored at −80°C. *Nicotiana benthamiana* leaves were co-infiltrated with *Agrobacterium* strains harboring the recombinant vector and plasma membrane localization markers. After 48 h of dark incubation at 25°C, fluorescence signals were observed under an Olympus FV3000 confocal laser scanning microscope.

### Drought stress treatment

Hydroponic simulated drought: Two-day-old seedlings with consistent growth (16 plants per line) were used, with normal nutrient solution as the control, and treated with 10% PEG (mild drought) for 8 d. Plant height and root length were measured with a vernier caliper.

Extreme drought in soil (three-leaf stage): Two-day-old seedlings were transplanted into nutrient soil (16 plants per line, 9 biological replicates) and watered with 150 mL every 2 d. Water was withheld after 10 d of growth, and drought stress was initiated when the soil water content decreased to 16% at the three-leaf stage (detected by a soil moisture meter). Because the overexpression (OE) and knockout (KO) lines were generated in two different *japonica* backgrounds, Nipponbare and Zhonghua 11, which exhibit intrinsic differences in basal drought tolerance, the stress duration for each background was determined in preliminary experiments to achieve a comparable moderate stress intensity, rather than an equal treatment duration. Specifically, stress was initiated when soil water content decreased to approximately 16% in both backgrounds. Accordingly, the Nipponbare-based lines (OE lines and their wild-type controls) were rewatered after 3 days of stress, whereas the Zhonghua 11-based lines (KO lines and their wild-type controls) were rewatered after 4 days of stress. Survival rates were scored after 9 days of recovery. All statistical comparisons were performed exclusively within the same genetic background, and cross-background observations were interpreted only as qualitative trends.

Extreme drought in soil (four-leaf stage): Seeds were raised in the field, and seedlings at the three-leaf stage were transplanted into plastic pots (16 plants per line, 3 biological replicates). Water was withheld after 20 d of growth, and stress was initiated when the soil water holding capacity reached 20% at the four-leaf stage. Plants were rewatered after 30 d of stress, and the survival rate was determined after 17 d of recovery.

### Physiological measurements and staining

Shoot tissues of wild-type and transgenic seedlings at the three-leaf stage were collected before treatment and after 20% PEG treatment for 2 d. Approximately 0.1 g of tissue was homogenized in 100 mmol/L phosphate buffer (pH 7.0) on ice, centrifuged at 8000 g for 15 min at 4℃, and the supernatant was used to determine SOD activity and the contents of MDA, soluble sugar and proline according to [68]. All experiments and analyses were performed using samples collected from the same batch. Three technical replicates were completed for each sample.

The second true leaves before treatment and after 20% PEG treatment for 4 d were collected and immersed in 1 mg/mL DAB and 6 mmol/L NBT solutions, respectively. After incubation at 28℃ under light for 12 h and decolorization with absolute ethanol, ROS accumulation was observed and photographed.

### Water loss rate

Detached leaf water loss assay: Three seedlings at the three-leaf stage per line were grouped with 3 biological replicates. After measuring the initial fresh weight (FW), the seedlings were placed in a constant temperature and humidity chamber (28°C, 40% humidity), and the dehydrated aerial weight (DAW) was measured hourly for 6 h. Water loss rate = (FW − DAW) / FW × 100%.

Intact plant water loss assay: Whole three-leaf-stage seedlings were weighed after root drying with filter paper (FW1), then reweighed after 20% PEG treatment for 2 d (FW2). The residual weight (FWr) was recorded after removing shoots. Water loss rate = [(FW1 − FWr) − (FW2 − FWr)] / (FW1 − FWr) × 100%.

### Observation of stomatal morphological characteristics

Wild-type and transgenic lines were grown to the three-leaf stage and treated with 20% PEG alone for 8 h, while overexpression lines were additionally treated with 20% PEG combined with 50 μmol/L ABA for 2 d. After leaf sampling and fixation with FAA fixative, critical point drying (Quorum K850, UK) was performed by Chengdu Lilaikeshuo Medical Laboratory, and stomatal morphology was observed using a scanning electron microscope (FEI Inspect, USA). For each line, five visual fields were randomly selected for statistical analysis of stomatal aperture and density. The stomatal pore index (SPI), calculated as SPI = SLen^2^ × SD × 10 ^-4^ [69], serves as an approximate measure of the proportion of stomatal pore area to leaf area and is widely used as an indicator of stomatal opening degree in crop leaves [70]. Here, SD represents stomatal density and SLen represents stomatal length.

### ABA treatment and content determination

Two-day-old seedlings were subjected to treatments with 15 μmol/L ABA, normal hydroponic solution, and 10/20 μmol/L diniconazole (an ABA synthesis inhibitor), respectively. Plant height was measured after 8 days of cultivation. Sixteen plants per line were evaluated.

Determination of endogenous hormones: 1 g of shoot tissue was collected from three-leaf stage seedlings before treatment and after 2 days of 20% PEG treatment, then snap-frozen in liquid nitrogen and stored at −80°C. The samples were sent to Nanjing Weibairui Detection Co., Ltd. for endogenous hormone analysis. Hormones were extracted with acetonitrile and purified via the QuEChERS method, and their contents were determined using an ultra-performance liquid chromatography-triple quadrupole mass spectrometer (UPLC-QQQ, PerkinElmer). Internal standards were added to the extract for result calibration. All determinations were performed with two technical replicates, and the average values were used for subsequent analysis.

### Nitrate treatment

Two-day-old seedlings were cultivated in modified Hoagland nutrient solution devoid of nitrogen and calcium nitrate. The normal control group was supplied with 2 mmol/L NO_3_^-^ without any background nitrogen source. The different treatment groups (4-day-old) were supplemented with nitrate (supplied as sodium nitrate) at concentrations of 0, 0.2, 2, or 10 mmol/L, respectively. All groups received 1 mmol/L glutamine as a background nitrogen source to maintain basal nitrogen supply and avoid nitrogen starvation stress [71]. Sixteen plants per line were evaluated. After 8 days of culture at 28 °C under a 16 h light / 8 h dark photoperiod, root length was measured using a vernier caliper.

### RNA sequencing and analysis

Nipponbare wild-type and overexpression seedlings at the three-leaf stage were treated with 20% PEG6000 for 0, 1, 8, 16, and 24 h, and shoots were collected (with three biological replicates per sample). After flash freezing in liquid nitrogen, RNA purification, library construction and high-throughput sequencing were performed by OE Biotech Co., Ltd. (Shanghai). Gene expression levels were normalized using FPKM (Fragments Per Kilobase per Million reads) and visualized as a heatmap. Differential expression analysis was performed using raw read counts as input, with median-of-ratios normalization, fold change calculation, and Wald test based on negative binomial distribution implemented in DESeq2. Differentially expressed genes were identified within the set of protein-coding genes using the thresholds of |log_2_FC| ≥ 1 and FDR < 0.05 [72].Bioinformatics analysis and visualization were conducted on the OECloud platform. Several DEGs were selected to verify transcriptome results by RT-qPCR (0 h and 1 h samples).

## Supporting information

S1 Fig DUF1223 protein sequence analysis and identification of *OsDUF1223* transgenic lines. (TIF)

S2 Fig *OsDUF1223* transmembrane structure and signal peptide prediction. (TIF)

S3 Fig *OsDUF1223* transgenic plants’ drought tolerance at the four-leaf stage. (TIF)

S4 Fig Quantitative analysis of NBT and DAB staining in leaves of *OsDUF1223* transgenic rice lines. (TIF)

S5 Fig Transcriptome analysis of *OsDUF1223* OE plant under drought stress for 0 h. (TIF)

S6 Fig KEGG enrichment analysis of transcriptome data from *OsDUF1223* OE plants. (TIF)

S7 Fig Water loss rate and stomatal morphological characteristics of *OsDUF1223* Transgenic Lines During ABA Replenishment Under Drought Stress. (TIF)

S1 Table The results of quantitative co-localization analysis. (XLSX)

S2 Table Endogenous hormone contents in ZH11 and KO line seedlings at the three-leaf stage under normal conditions and drought stress. (XLSX)

S3 Table Endogenous hormone contents in WT and OE line seedlings at the three-leaf stage under normal conditions and drought stress. (XLSX)

S4 Table RT-qPCR primers. (XLSX)

S5 Table Primers required for vector construction and positive detection. (XLSX)

S1 Data Source data for all of the main figures. (XLSX)

S2 Data Source data for all of the supplemental figures. (XLSX)
