# Supplementary material for: OsDUF1223 negatively regulates seedling drought tolerance and modulates nitrate-dependent root growth in rice
Source: PLoS Genet. 2026 Sep 3;22(9):e1012295. doi: 10.1371/journal.pgen.1012295 (PMC13541127; doi:10.1371/journal.pgen.1012295)
Supplement: Supplementary file 1 [file pgen.1012295.s016.docx]

**Response Letter to Reviewers’ Comments**

**Comments from the Editor**:

Thank you for submitting your manuscript to PLOS Genetics. After careful consideration, we feel that it has merit but does not fully meet PLOS Genetics's publication criteria as it currently stands. Therefore, we invite you to submit a revised version of the manuscript that addresses the points raised during the review process.

**Response**: We thank all the editors and reviewers for their valuable comments and suggestions. We have carefully revised the manuscript overall to enhance its clarity and facilitate the understanding of the readers. Our point-to-point responses are presented in the following and bule text is the revised content. We hope that the revision would satisfactorily address the comments and concerns of the editors and reviewers.

Reviewer #1:

Major comments:

1.The number of biological replicates used throughout the study is insufficient to support robust statistical conclusions. Most experiments were performed with only n = 3 biological replicates, which substantially limits the reliability and reproducibility of the presented data. The authors should increase the number of biological replicates (n=10-15) and provide additional experimental validation. In its current form, the statistical power of the analyses appears inadequate.

Response：We appreciate your important comments regarding the number of biological replicates. We would like to clarify that our experimental design involved planting 16 individuals per genotype/line with 3 biological replicates, as already described in the Materials and Methods section. We acknowledge that this information was indicated in the figure legends, but may have caused some data misinterpretation. In the revised manuscript, we have clearly indicated the sample size for all experiments in the Materials and Methods section to ensure transparency. Regarding sample size, we further clarify that all phenotypic experiments were performed with a sample size of at least 16 individuals. We believe this sample size is sufficient to support the reliability of the statistical analyses.

2.Regarding lines 123–124, the authors state that OsDUF1223 was identified from publicly available microarray datasets; however, the supporting data are not shown in the manuscript. The authors should include the corresponding transcriptomic analysis demonstrating how OsDUF1223 was selected. In particular, expression profiling such as a heatmap should be provided to demonstrate that OsDUF1223 is specifically induced under drought stress conditions.

Response：Thank you for your comments. We apologize that the original microarray data mentioned in lines 123–124 have been lost due to unforeseen circumstances and cannot be provided. However, the experimental data in the current manuscript are sufficient to demonstrate the basis for selecting *OsDUF1223*. As shown in Figure 1A–B, the expression level of *OsDUF1223* was significantly upregulated under treatment with 20% PEG (simulating drought) and 50 μmol/L ABA, directly demonstrating that this gene responds to drought stress. We have revised the relevant statements in the manuscript to clarify that the gene was selected based on initial microarray data and subsequent experimental validation.

*(See the paragraph of Section “Results | OsDUF1223 is specifically expressed at the seedling stage and responds to both drought stress and exogenous ABA”)*

Based on publicly available transcriptomic data and subsequent experimental validation from rice seedlings under drought and ABA treatments, we hypothesized that *OsDUF1223* may be involved in drought stress and ABA signal responses.

3.The biological and evolutionary context of OsDUF1223 remains unclear. The authors should clarify whether OsDUF1223 belongs to a gene family in rice and whether homologs are conserved in other plant species. Such information would help readers evaluate the broader significance and potential universality of the findings.

Response：Thank you for your valuable suggestion. To elucidate the evolutionary and biological background of *OsDUF1223*, we selected DUF1223 family members from rice, Arabidopsis, wheat, barley, cucumber, soybean, tomato, *Populus tomentosa*, and maize for phylogenetic analysis and protein sequence analysis. As illustrated in the figure, *OsDUF1223* (Os07t0559400, highlighted in a red box) exhibits the closest evolutionary relationship with barley HG0135400.1.

*(See the paragraph of Section “Results | Bioinformatics analysis”)*

The gene encoding *OsDUF1223* is located on chromosome 7, with an open reading frame encoding 274 amino acid residues and a predicted molecular weight of approximately 28.97 kDa. To investigate the evolutionary conservation of the *OsDUF1223* protein, we analyzed DUF1223 family members from rice (*Oryza sativa* L.), *Arabidopsis thaliana* (L.) Heynh., wheat (*Triticum aestivum* L.), barley (*Hordeum vulgare* L.), cucumber (*Cucumis sativus* L.), soybean (*Glycine max* (L.) Merr.), tomato (*Solanum lycopersicum* L.), white poplar (*Populus tomentosa Carrière*), and maize (*Zea* *mays* L.). Phylogenetic analysis revealed strong sequence conservation among these orthologs.

**Figure S1A. Sequence analysis of the DUF1223 protein family**

Note: The figure shows DUF1223 members from different species, including rice (*Oryza sativa*, Os07t05559400), *Arabidopsis thaliana* (AT4G27350.1, AT5G54240.1), wheat (*Triticum aestivum*, TraesCS2D02G210400.1, TraesCS2A02G205700.1, TraesCS2B02G233000.1), barley (*Hordeum vulgare*, HG0135400.1), cucumber (*Cucumis sativus*, KGN59870), soybean (*Glycine max*, KRH25853), tomato (*Solanum lycopersicum*, Solyc07g063620.3.1), *Populus trichocarpa* (Potri.001G408001.1, Potri.011G126300.2), and maize (*Zea mays*, Zm00001eb324170). The red box indicates *OsDUF1223*.

4. In Fig. 2D, the OsDUF1223 overexpression lines exhibit increased root length. However, this phenotype is not observed in Fig. 8I under the nitrate 2 mM and 10 mM conditions. The authors should clearly indicate the nitrogen concentrations used in each experiment and discuss the possible reasons underlying these apparently inconsistent phenotypic results.

Response：Thank you for your attention regarding the consistency of phenotypic results between Figure 2D and Figure 8I. Below we provide explanations regarding the nitrogen conditions used in each experiment and the reasons for the observed differences in results:

(1) Description of nitrogen conditions in each experiment

Figure 2D: Plants were cultivated using standard hydroponic solution (conventional Hoagland nutrient solution containing complete nitrogen sources).

Figure 8C: Plants were cultivated using modified Hoagland nutrient solution (basal solution without any form of nitrogen source), supplemented with 2 mmol/L nitrate treatment.

Figure 8I: Plants were cultivated using modified Hoagland nutrient solution (basal solution without any form of nitrogen source), with a nitrate gradient of 0, 0.2, 2, and 10 mmol/L, and supplemented with 1 mmol/L glutamine as a background nitrogen source, simulating nitrogen-free, low-nitrogen, normal-nitrogen, and high-nitrogen conditions, respectively.

(2) Analysis of reasons for differences between Figure 2D and Figure 8I

As shown in Figure 8C, when no background nitrogen source was added and 2 mmol/L nitrate served as the sole nitrogen source, the root length of overexpression lines was significantly longer than that of the wild type, consistent with the results in Figure 2D. However, in Figure 8I, due to the addition of 1 mmol/L glutamine as a background nitrogen source in all treatment groups, no significant difference in root length was observed between overexpression lines and the wild type under 2 mmol/L and 10 mmol/L nitrate treatments. A possible reason for this discrepancy is that glutamine, as a direct product of nitrogen assimilation, can be preferentially utilized by plants, thereby reducing their dependence on exogenous nitrate. This effect masked the advantage of *OsDUF1223* overexpression lines in nitrate absorption and assimilation, resulting in no significant difference in root length phenotype under 2 mmol/L and 10 mmol/L nitrate treatments.

In the revised manuscript, we have clearly indicated the nitrogen conditions used for each treatment in the Materials and Methods section, and supplemented the explanation of the phenotypic differences between Figure 2D and Figure 8I and their underlying reasons in the Results section.

*(See the paragraph of Section “Fig 8| Note”)*

Root system architecture phenotypes and root length statistics of transgenic lines cultured in modified nitrogen-free Hoagland medium containing 2 mmol/L NO₃⁻ for 12 days: C–D. Nipponbare WT and OE lines; E–F. Zhonghua 11 WT and KO lines. Scale bar = 2 cm. Root system architecture phenotypes and root length statistics of transgenic lines cultured in modified nitrogen-free Hoagland medium containing different NO₃⁻ concentrations (with 1 mmol/L glutamine as background nitrogen source) for 8 days: G–I. Nipponbare WT and OE lines; I–J. Zhonghua 11 WT and KO lines. Scale bar = 2 cm.

*(See the paragraph of Section “Results | OsDUF1223 affects the drought tolerance of rice by participating in the regulation of root architecture mediated by nitrogen”)*

Moreover, under 2 mmol/L NO₃⁻ and 10 mmol/L NO₃⁻ treatment, there was no significant difference in root length between each transgenic line and the wild type. This may be because glutamine, as a direct product of nitrogen assimilation, can be preferentially utilized by plants, thereby reducing their dependency on exogenous nitrate^[46, 47]^.

*Added reference*:

46.	Svietlova N, Zhyr L, Reichelt M, Grabe V, Mithöfer A. Glutamine as sole nitrogen source prevents induction of nitrate transporter gene NRT2.4 and affects amino acid metabolism in Arabidopsis. Front Plant Sci. 2024;15:1369543.

47.	Pařízková B, Johansson AI, Juvany M, Šimura J, Ljung K, Antoniadi I. L-Glutamine Modulates Root Architecture and Hormonal Balance in Arabidopsis. Physiol Plant. 2026;178(1):e70723.

Minor Comments

1.The discussion in lines 94–98 regarding nitrate ion uptake appears unnecessary, as this study does not experimentally address nitrate uptake mechanisms. This section of the Introduction should therefore be shortened or removed.

Response：Thank you for your suggestion. We agree that the description of nitrate uptake mechanisms (LATS/HATS) in the original manuscript was unnecessary. In the revised version, we have streamlined this section, retaining only the core information that "nitrate can act as a signaling molecule," and have removed the detailed discussion of LATS and HATS.

*(See the paragraph of Section “Introduction”)*

Nitrate and ammonium are the primary inorganic nitrogen forms taken up and utilized by higher plants, among which NO₃⁻ is the major nitrogen source for the majority of non-leguminous plants^[32, 33]^ . Plants complete NO₃⁻ uptake through transporters localized on the root cell plasma membrane, after which it is further reduced to ammonium and incorporated into organic nitrogen compounds, ultimately forming amino acids, proteins, and other nitrogen-containing compounds^[34, 35]^. In addition, nitrogen and its metabolites also act as signaling molecules that regulate the expression of nitrogen-responsive genes and activate signaling pathways involved in the regulation of diverse biological processes. As the primary organ for nitrogen uptake, roots exhibit remarkable structural plasticity in response to changes in the form, abundance, and distribution of soil nitrogen. Nitrogen-dependent regulation of root system architecture (RSA) relies on the integration of local and systemic nitrogen signals ^[36]^, and plant hormones also play a critical role in systemic nitrogen signal transduction^[37-39]^.

*Added reference*:

32.	Xu G, Fan X, Miller AJ. Plant nitrogen assimilation and use efficiency. Annu Rev Plant Biol. 2012;63:153–82. Epub 20120103.

33.	Kronzucker HJ, Glass ADM, Siddiqi MY, Kirk GJD. Comparative kinetic analysis of ammonium and nitrate acquisition by tropical lowland rice: implications for rice cultivation and yield potential. New Phytol. 2000;145(3):471–6.

34.	Liu KH, Niu Y, Konishi M, Wu Y, Du H, Sun Chung H, et al. Discovery of nitrate-CPK-NLP signalling in central nutrient-growth networks. Nature. 2017;545(7654):311–6.

35.	Vidal EA, Álvarez JM, Moyano TC, Gutiérrez RA. Transcriptional networks in the nitrate response of Arabidopsis thaliana. Curr Opin Plant Biol. 2015;27:125–32.

36.	Ruffel S, Krouk G, Ristova D, Shasha D, Birnbaum KD, Coruzzi GM. Nitrogen economics of root foraging: transitive closure of the nitrate-cytokinin relay and distinct systemic signaling for N supply vs. demand. Proc Natl Acad Sci U S A. 2011;108(45):18524–9.

37.	Abualia R, Ötvös K, Novák O, Bouguyon E, Domanegg K, Krapp A, et al. Molecular framework integrating nitrate sensing in root and auxin-guided shoot adaptive responses. Proc Natl Acad Sci U S A. 2022;119(31):e2122460119. Epub 20220725.

38.	Poitout A, Crabos A, Petřík I, Novák O, Krouk G, Lacombe B, et al. Responses to Systemic Nitrogen Signaling in Arabidopsis Roots Involve trans-Zeatin in Shoots. Plant Cell. 2018;30(6):1243–57.

39.	Forde BG. Nitrogen signalling pathways shaping root system architecture: an update. Curr Opin Plant Biol. 2014;21:30–6. Epub 20140703.

2.The plasma membrane marker used in Fig. 1F should be clearly described in the Materials and Methods section.

Response：Thank you for your suggestion. We have added a description of the plasma membrane markers in the Materials and Methods section.

*(See the paragraph of Section “Materials and methods | Subcellular localization of OsDUF1223 protein”)*

Meanwhile, the coding sequence of the *Arabidopsis thaliana* plasma membrane aquaporin gene *AtPIP2A* was cloned into the pK7m34GW vector to generate the *AtPIP2A*-mCherry fusion construct^[65]^, which served as a plasma membrane marker.

*Added reference*:

65.	Nelson BK, Cai X, Nebenführ A. A multicolored set of in vivo organelle markers for co-localization studies in Arabidopsis and other plants. Plant J. 2007;51(6):1126–36.

3. The abbreviation “SOD activity” in line 226 should be defined at its first appearance in the main text.

Response：Thank you for pointing out this issue. We have defined this abbreviation at its first occurrence in the main text. In the revised manuscript, it now reads: "superoxide dismutase (SOD) activity."

*(See the paragraph of Section “Results | Determination of Drought Tolerance Indicators in OsDUF1223 Transgenic Plants”)*

To further confirm that *OsDUF1223* negatively regulates ROS scavenging capacity, the activity of superoxide dismutase (SOD) activity was determined in this study.

Reviewer #2:

 This work reports the identification of a novel rice gene OsDUF1223 with potential functions in regulating drought tolerance, and the research strategy adopted shows distinct innovative value. While the study delivers promising preliminary insights, there remain several key aspects related to experimental design, data explanation and logical coherence that could be further optimized to strengthen the robustness of the conclusions. The manuscript will be suitable for publication provided all the concerns outlined below are fully addressed. Specific points for improvement are listed as follows:

1. The title of Figure 1, “Expression pattern analysis and subcellular localization of the OsDUF1223 gene,” is imprecise and should refer to the subcellular localization of the OsDUF1223 protein. In addition, the RFP marker used in Figure 1F is described as a plasma membrane marker (lines 162–164), but neither the main text nor the Materials and Methods section identifies this marker. Furthermore, the authors state in line 165 that the RFP and GFP signals “perfectly overlapped,” yet the merge in Figure 1F does not show complete colocalization. Quantitative colocalization analysis or clearer magnified images are recommended.

Response：Thank you very much for your valuable comments. In response to the three issues you raised, we have made the following revisions to the manuscript.

*(See the paragraph of Section “Figure 1”)*

Expression pattern analysis of the *OsDUF1223* gene and subcellular localization of its protein

*(See the paragraph of Section “Materials and methods | Subcellular localization of OsDUF1223 protein”)*

Meanwhile, the coding sequence of the *Arabidopsis thaliana* plasma membrane aquaporin gene *AtPIP2A* was cloned into the pK7m34GW vector to generate the *AtPIP2A*-mCherry fusion construct^[65]^, which served as a plasma membrane marker.

*Added reference*:

65.	Nelson BK, Cai X, Nebenführ A. A multicolored set of in vivo organelle markers for co-localization studies in Arabidopsis and other plants. Plant J. 2007;51(6):1126–36.

*(See the paragraph of Section “Results | Generation and Subcellular Localization of OsDUF1223 Transgenic Plants”)*

To investigate the subcellular localization of the *OsDUF1223*-GFP fusion protein, co-localization assays were performed using a plasma membrane marker (*AtPIP2A*-mCherry) in tobacco epidermal cells. As shown in Fig 1F, the 35S:*OsDUF1223*-GFP signal (green) and the *AtPIP2A*-mCherry signal (red) exhibited substantial colocalization at the plasma membrane. Quantitative colocalization analysis (Table S1) revealed a Pearson's correlation coefficient of 0.835, with Manders' coefficients of M1 = 0.809 (GFP overlapping with RFP) and M2 = 0.802 (RFP overlapping with GFP), further confirming the plasma membrane localization of *OsDUF1223*.

Table S1 The results of quantitative co-localization analysis

| Group | Pearson’s Coefficient | M1 | M2 |
| --- | --- | --- | --- |
| 35S:GFP(Figure 1F) | 0.778 | 0.753 | 0.606 |
| 35S:*OsDUF1223*-GFP(Figure 1F) | 0.835 | 0.809 | 0.802 |

2. In lines 168–192, overexpression (OE) lines were generated in the Nipponbare background, while knockout (KO) lines were generated in the Zhonghua 11 background. Although both are japonica varieties, the genetic backgrounds are not identical. Consequently, all transgenic lines can only be compared with their respective wild type controls, and direct phenotypic comparisons between OE and KO lines are not valid. Moreover, in Figures 2G–J, the survival rates of ZH11 and Nipponbare under the same treatment differ substantially, making it difficult to determine whether the observed phenotypes are directly linked to OsDUF1223 expression levels. Please also explain why the drought treatment duration for OE lines (3 days) and KO lines (4 days) was not consistent in the experiments shown in Figures 2G–J.

Response：Thank you for your valuable comments. We fully agree with the reviewer's viewpoint that although Nipponbare and Zhonghua 11 both belong to the *japonica* subspecies, there are differences in their genetic backgrounds. Therefore, direct phenotypic comparisons between overexpression lines and knockout lines are not rigorous. We have corrected this issue in the revised manuscript. Since the two wild-type varieties (Zhonghua 11 and Nipponbare) exhibit different basal tolerance to drought stress, we have determined separate drought treatment durations for each: Nipponbare is relatively sensitive to drought, with significant genotype-specific phenotypic differences observable after 3 days of treatment; in contrast, Zhonghua 11 is more tolerant and requires 4 days of treatment to clearly distinguish the response differences among genotypes. Accordingly, the treatment duration for the OE lines and their corresponding controls was 3 days, while the treatment duration for the KO lines and their corresponding controls was 4 days. The detailed treatment procedures have been described in the Materials and Methods section.

*(See the paragraph of Section “Abstract”)*

Under drought stress, *OsDUF1223*-overexpressing lines exhibited lower survival rates, higher water loss rates, and reduced antioxidant enzyme activities and osmolyte accumulation, while knockout lines displayed the opposite phenotypes. *OsDUF1223* promotes transpirational water loss by increasing stomatal aperture and decreasing stomatal density, and mediates root system architecture remodeling in a nitrate-dependent manner.

*(See the paragraph of Section “Results | OsDUF1223 Reduces Drought Tolerance in Rice Seedlings”)*

Under normal conditions, no significant differences in plant height were observed among all lines. However, after 8 days of 10% PEG treatment, the plant height of overexpression lines was significantly lower than that of the wild type Nipponbare; in contrast, the knockout lines showed no obvious changes compared with ZH11. For root traits, under normal conditions, overexpression lines exhibited significantly longer roots than the wild type; conversely, the root length of the knockout lines was significantly shorter than that of ZH11.

Three-leaf stage pot assays showed that overexpression lines displayed more severe wilting under drought stress, with survival rates of 29.9%, 33.4%, and 41.7% after rewatering, which were significantly lower than the 68.8% of the Nipponbare. In contrast, compared with ZH11, the knockout lines showed milder wilting, with survival rates of 56.2% and 64.8%, which were significantly higher than that of their wild type (Fig 2G–J).

Collectively, overexpression of *OsDUF1223* reduced drought tolerance in rice seedlings, whereas knockout enhanced drought resistance, demonstrating that *OsDUF1223* acts as a negative regulator of drought stress responses at the rice seedling stage.

*(See the paragraph of Section “Results | Determination of Drought Tolerance Indicators in OsDUF1223 Transgenic Plants”)*

however, after drought treatment, the DAB and NBT staining signals in the leaves of overexpression lines in the Nipponbare background were significantly enhanced, indicating explosive ROS accumulation, in contrast, the staining signals in knockout lines in the ZH11 background were significantly weakened (Fig 3A–B).

The results showed no significant difference in superoxide dismutase (SOD) activity among all lines under normal conditions; after drought treatment, the SOD activity of overexpression lines was significantly lower than that of Nipponbare; in contrast, under the ZH11 background, the SOD activity of the knockout lines was significantly higher than that of ZH11 (Fig 3C–D).

Notably, under normal conditions, the soluble sugar content in some overexpression lines was significantly higher than that in Nipponbare; in contrast, under the ZH11 background, no significant difference was observed in the knockout lines. After drought treatment, the contents of proline and soluble sugars in overexpression lines were significantly lower than those Nipponbare; conversely, under the ZH11 background, these contents in the knockout lines were significantly higher than those in ZH11 (Fig 3E–H).

After drought treatment, the MDA content of overexpression lines was significantly higher than that of the Nipponbare, indicating more severe cell membrane damage; in contrast, under the ZH11 background, the MDA content of the knockout lines was significantly lower than that of ZH11 (Fig 3I–J).

*(See the paragraph of Section “Results | Generation and Subcellular Localization of OsDUF1223 Transgenic Plants”)*

The results showed that under 20% PEG-induced severe drought stress, the water loss rate of overexpression lines was significantly higher than that of Nipponbare; in contrast, under the ZH11 background, the water loss rate of the knockout lines was significantly lower than that of the wild type (Fig 4A–B).

*(See the paragraph of Section “Results | OsDUF1223 modulates the expression of genes related to hormone signaling pathways and affects the homeostasis of endogenous hormones”)*

Under both normal and drought conditions, the endogenous hormone levels in the knockout and overexpression lines differed from those in the wild type, with the most pronounced difference observed in ABA content. After drought treatment, the ABA content in the knockout lines was significantly higher than that in ZH11, whereas under both normal and drought conditions, the ABA content in the overexpression lines was lower than that in Nipponbare (Tables S2, S3).

*(See the paragraph of Section “Results | OsDUF1223 positively regulates the ABA signaling pathway, and ABA acts as an essential downstream regulatory signal for it”)*

Further investigations into the ABA pathway revealed that after treating two-day-old seedlings with 15 μmol/L ABA for 10 days, the plant height of overexpression lines was significantly lower than that of Nipponbare; in contrast, under the ZH11 background, the plant height of the knockout lines was significantly higher than that of ZH11 (Fig. 7A–D). When two-day-old seedlings were treated with different concentrations of diniconazole (an ABA synthesis inhibitor), the plant height of the overexpression lines was lower than that of Nipponbare at both 10 μmol/L and 20 μmol/L concentrations; in contrast, under the ZH11 background, the knockout lines showed significantly greater plant height than ZH11 only under 20 μmol/L diniconazole treatment.

*(See the paragraph of Section “Results | OsDUF1223 affects the drought tolerance of rice by participating in the regulation of root architecture mediated by nitrogen”)*

After 2-day-old seedlings were cultured in modified nitrogen-free Hoagland's medium containing 2 mmol/L NO₃⁻ for 12 days, the root lengths of *OsDUF1223* overexpression lines were 8.91 cm, 8.18 cm, and 7.54 cm, respectively, which were significantly longer than the 5.27 cm of Nipponbare. In contrast, under the ZH11 background, the root lengths of the knockout lines were 6.16 cm and 6.27 cm, respectively, which were significantly shorter than the 7.16 cm of ZH11 (Fig 8C–F).

After 8 days of culture, it was found that the root length of overexpression lines was significantly shorter than that of Nipponbare under nitrogen-free and low-nitrogen conditions, while no significant differences were observed under normal-nitrogen and high-nitrogen conditions. In contrast, under the ZH11 background, the knockout lines exhibited significantly longer roots than ZH11 under nitrogen-free and low-nitrogen conditions, while no significant differences were observed under normal-nitrogen and high-nitrogen conditions either (Fig 8G–J).

*(See the paragraph of Section “Discussion”)*

Phenotypic analysis revealed that *OsDUF1223*-knockout lines exhibited milder growth inhibition, less wilting, and higher survival rates after rewatering under drought stress, whereas overexpression lines in the Nipponbare background showed lower survival rates.

Nitrate concentration gradient experiments demonstrated that the root phenotype effects of *OsDUF1223* are dependent on nitrogen availability: under nitrogen-free or low-nitrogen conditions, OE lines exhibited significantly shorter roots than wild-type plants, whereas no difference was observed under high-nitrogen conditions. Conversely, KO lines in the ZH11 background showed the opposite trend,

3. In the abstract (lines 36–38), in the ABA signaling experiments (lines 379–397), and in the nitrogen response and root architecture experiments (lines 408–435), the authors repeatedly propose that OsDUF1223 “integrates ABA and nitrogen signals” to regulate drought tolerance. However, no direct molecular evidence (e.g., protein protein interactions, phosphorylation regulation) is provided to demonstrate that OsDUF1223 physically interacts with key components of ABA signaling or nitrogen metabolism pathways. The current conclusions are based primarily on transcriptomic and phenotypic data. The authors should clearly distinguish between correlation and causation and propose testable hypotheses for the molecular mechanism.

Response：Thank you very much for this critical comment. We fully agree that our previous conclusion that "*OsDUF1223* integrates ABA and nitrogen signals" was indeed an overinterpretation of the available data, which are primarily correlational rather than causal. In the revised manuscript, we have toned down the language throughout, replacing "integrates" with more cautious wording to avoid any implication that a causal relationship has been established.

*(See the paragraph of Section “Abstract”)*

Collectively, this study elucidates that *OsDUF1223* regulates drought tolerance at the seedling stage in rice through functional association with the ABA and nitrogen signaling pathways, thereby providing a candidate gene resource for the genetic improvement of drought tolerance in rice.

*(See the paragraph of Section “Introduction”)*

This study focused on the function of *OsDUF1223* in the drought stress response of rice seedlings. Using transcriptome sequencing and quantitative real-time PCR, we analyzed its regulatory network and obtained evidence suggesting that *OsDUF1223* may be associated with drought tolerance through its functional links to nitrogen and ABA signaling pathways.

*(See the paragraph of Section “Discussion”)*

This study provides evidence that *OsDUF1223* may be associated with drought tolerance in rice seedlings through its functional links to ABA signaling and nitrogen metabolism.

4. In lines 389–390, the authors state that “OsDUF1223 positively regulates the ABA signaling pathway.” Yet, Table S2 shows that endogenous ABA content is reduced in OsDUF1223 overexpression lines. If OsDUF1223 indeed positively regulates ABA signaling, why is endogenous ABA decreased? Moreover, if the overexpression lines are merely hypersensitive to ABA, why does treatment with an ABA synthesis inhibitor cause a more dramatic change in plant height (from ~17 cm to ~7 cm) than exogenous ABA application (from ~20 cm to ~11 cm)? The authors should provide a plausible explanation for this discrepancy.

Response：Thank you for raising these insightful questions. We have elaborated on both points in the Discussion section, and we now summarize them as follows.

Regarding whether the decrease in endogenous ABA content contradicts the "positive regulation of ABA signaling": This is not contradictory; rather, it precisely reflects the bidirectional regulation of the ABA system by *OsDUF1223*. The model we propose is that *OsDUF1223* exerts a dual function on the ABA signaling pathway: "positive regulation of signal transduction" and "negative feedback inhibition of synthesis." Specifically, *OsDUF1223* enhances the perception and transduction efficiency of ABA signals by inhibiting negative regulators such as *OsPP2C49* (positive regulation of signaling), while simultaneously suppressing the expression of ABA biosynthetic enzymes NCEDs through a feedback mechanism, thereby maintaining endogenous ABA at a low level (negative feedback inhibition of synthesis). This "low content, high sensitivity" regulatory model is not uncommon in plant hormone regulation — it allows plants to maintain a state of stress alertness without accumulating high levels of inhibitory hormones, achieving a balance between growth and defense.

Regarding the issue that the ABA biosynthesis inhibitor has a greater effect on plant height than exogenous ABA application: We believe this is related to the disruption of endogenous ABA homeostasis in the overexpression lines. The endogenous ABA level in the overexpression lines is already maintained at a low steady state due to feedback inhibition, while the ABA signaling pathway is highly sensitized. When exogenous ABA is applied, the plants can still partially buffer the effect using residual endogenous ABA synthesis capacity, and the ABA degradation pathway can also be activated to maintain dynamic equilibrium; therefore, the reduction in plant height is relatively moderate. In contrast, uniconazole directly inhibits key steps in ABA biosynthesis, fundamentally blocking the production of endogenous ABA. For the overexpression lines, which already have insufficient endogenous ABA and a highly sensitized signaling pathway, this effectively deprives them of the signaling molecules required to initiate ABA-dependent growth regulation, leading to more severe growth inhibition. Furthermore, uniconazole is not only an inhibitor but also a pesticide with certain toxicity; it causes growth inhibition through both ABA suppression and toxic side effects. Therefore, it should only be used as a supplementary experiment and should not be relied upon as the sole treatment.

*(See the paragraph of Section “Discussion”)*

This study found that endogenous ABA content in *OsDUF1223* overexpression lines was significantly lower than that in the wild type, yet their sensitivity to exogenous ABA was significantly higher, and they also exhibited stronger growth inhibition in response to treatment with the ABA degradation inhibitor diniconazole. This suggests that *OsDUF1223* exerts a dual regulatory effect on the ABA system. On one hand, within the bZIP23–*OsPP2C49* cascade, although the downregulation of *OsbZIP23* attenuates its positive regulatory function in the activation of ABA-responsive genes, the net phenotype is dominated by the reduction of the terminal negative regulator *OsPP2C49*—because PP2Cs function as gatekeepers that directly dephosphorylate and inactivate SnRK2s; their removal can sensitize the signaling pathway even when ABA ligand levels are moderately reduce. Transcriptional changes alone do not fully account for this outcome; post-transcriptional regulation of *OsbZIP23* protein activity and potential functional compensation by other bZIP family members may further reinforce the hypersensitive phenotype. On the other hand, the downregulation of *OsbZIP23* leads to attenuated NCED-dependent ABA biosynthesis, maintaining endogenous ABA at a low steady-state level. This "low-content, high-sensitivity" regulatory model allows plants to maintain a stress-alert state without accumulating high concentrations of inhibitory hormones, thereby achieving a balance between growth and defense ^[56]^. Exogenous ABA complementation experiments further confirmed that ABA is an essential downstream signaling molecule for *OsDUF1223* in regulating drought tolerance: under combined treatment with 20% PEG and 50 μmol/L ABA, the differences in water loss rate among all lines were eliminated; scanning electron microscopy observations also indicated that the combined treatment induced stomatal closure in the overexpression lines, demonstrating that exogenous ABA could rescue the drought tolerance defect caused by *OsDUF1223* overexpression. Furthermore, differentially expressed genes enriched in the "stress response" pathway included multiple ABA-dependent stress response factors: *OsNAC52* can transcriptionally activate ABA-inducible gene expression ^[57]^, and *OsANN4* can mediate ABA-induced calcium influx and regulate antioxidant enzyme activity ^[58]^. These findings further support the role of *OsDUF1223* in regulating drought tolerance through the ABA pathway.

*Added reference*:

56.	Karasov TL, Chae E, Herman JJ, Bergelson J. Mechanisms to Mitigate the Trade-Off between Growth and Defense. Plant Cell. 2017;29(4):666–80.

57.	Gao F, Xiong A, Peng R, Jin X, Xu J, Zhu B, et al. OsNAC52, a rice NAC transcription factor, potentially responds to ABA and confers drought tolerance in transgenic plants. Plant Cell, Tissue and Organ Culture (PCTOC). 2010;100(3):255–62.

58.	Zhang Q, Song T, Guan C, Gao Y, Ma J, Gu X, et al. OsANN4 modulates ROS production and mediates Ca2+ influx in response to ABA. BMC Plant Biology. 2021;21(1):1–15.

5. In lines 408–409 and elsewhere in the manuscript, the authors propose that “OsDUF1223 affects drought tolerance by regulating nitrogen mediated root architecture.” However, OE lines have longer roots than the wild type, while KO lines have shorter roots. Why would a shorter rooted line be more drought tolerant? Furthermore, under 10% PEG simulated drought conditions, the difference in root length between OE and KO lines disappears, whereas their drought tolerance phenotypes remain distinct. This indicates that the observed differences in root architecture lack a logical causal relationship with drought tolerance.

Response：We appreciate your in-depth analysis. We acknowledge the logical contradiction you raised: if *OsDUF1223* affects drought tolerance by regulating nitrogen-mediated root system architecture, then the OE lines (with longer roots) should exhibit a greater advantage in drought tolerance, and when the root length difference disappears under PEG stress, the difference in drought tolerance should also disappear accordingly — however, the experimental results do not support this. In response to this observation, we propose that *OsDUF1223* primarily influences drought tolerance by regulating the nitrogen metabolism pathway; changes in root architecture are merely a phenotypic manifestation downstream of its regulatory effects on nitrogen response, rather than a direct causal mechanism underlying drought tolerance.

Transcriptomic analysis provides important clues for the involvement of *OsDUF1223* in nitrogen response. KEGG enrichment analysis showed that, regardless of whether the samples were collected before drought treatment (0 h) or after treatment (8 h, 16 h, 24 h), the differentially expressed genes in the OE lines were consistently enriched in the "nitrogen metabolism" pathway, suggesting that the regulation of nitrogen metabolism by *OsDUF1223* persists throughout the entire drought stress process, rather than being limited to the root development level. Furthermore, one of the overlapping up-regulated differentially expressed genes, *OsRNR10*, is known to inhibit NO₃⁻ uptake by regulating auxin accumulation, indicating that the regulatory network of *OsDUF1223* directly involves nitrogen uptake and assimilation, rather than indirectly affecting nitrogen acquisition solely through root elongation.

The fact that the root length difference disappears under PEG stress while the difference in drought tolerance persists precisely indicates that absolute root length is not the decisive factor for drought tolerance. Specifically: under normal conditions, *OsDUF1223* reshapes root architecture by regulating nitrogen metabolism genes (e.g., *OsRNR10*, etc.), which represents a phenotypic output of nitrogen response. When drought stress occurs, plants initiate root plasticity responses: the roots of OE lines undergo greater growth inhibition, while those of KO lines are less suppressed, leading to the disappearance of the root length difference — this "disappearance of the difference" is itself an adaptive re-adjustment, rather than indicating that roots are no longer important. However, the difference in drought tolerance persists because the regulation of the nitrogen metabolism pathway by *OsDUF1223* remains active under drought stress (the nitrogen metabolism pathway is continuously enriched at 8 h, 16 h, and 24 h). Differences in nitrogen metabolism efficiency affect the plant's overall osmotic adjustment, antioxidant capacity, and water use efficiency — these are the direct physiological bases underlying the difference in drought tolerance.

*(See the paragraph of Section “Discussion”)*

Our study demonstrates that *OsDUF1223* positively regulates root elongation in rice seedlings. Under normal conditions, overexpression (OE) lines in the Nipponbare background exhibited significantly longer roots, while knockout (KO) lines in the ZH11 background displayed markedly shorter roots. However, under mild drought stress, these differences diminished: OE lines showed greater root growth inhibition, whereas KO lines exhibited milder suppression, indicating that root plasticity is dynamically modulated by water status. The disappearance of root length differences under drought, while drought tolerance differences persisted, suggests that root architecture is not a direct causal pathway through which *OsDUF1223* regulates drought tolerance. Transcriptomic analyses provided key insights into the core mechanism: KEGG enrichment analysis showed that at the 0 h time point, upregulated differentially expressed genes in the overexpression lines were already significantly enriched in the "nitrogen metabolism" pathway. This phenomenon may partially reflect genotypic homeostatic background differences, including the influence of overexpression vector insertion effects, and should not be directly attributed to the drought response mechanism. In contrast, at the 8 h, 16 h, and 24 h time points, differentially expressed genes were consistently and significantly enriched in this pathway, suggesting that the drought response specificity of the "nitrogen metabolism" pathway should be primarily judged based on dynamic transcriptional changes during the later stages of stress. Taken together, *OsDUF1223* participates in the drought response by regulating nitrogen metabolism, and its function is not limited to root development.

Reviewer #3:

In this manuscript, Xiulin Zhao, Ziyi Wang and colleagues investigate the function of OsDUF1223 (LOC_Os07g37310), a chromosome-7 gene encoding a 274-aa protein of unknown function, in the drought response of rice seedlings. Using overexpression (OE) lines in the Nipponbare background and CRISPR knockout (KO) lines in the Zhonghua 11 background, the authors report that OsDUF1223 acts as a negative regulator of seedling drought tolerance, and they propose that it does so by simultaneously tuning ABA signaling/homeostasis and nitrate-dependent root architecture.
The main findings of the paper are:
OsDUF1223 is induced by PEG (drought) and exogenous ABA, is expressed mainly in seedling shoots, and localizes to the plasma membrane in transient N. benthamiana assays (Fig 1).
Under drought, OE lines show lower survival, higher water loss, stronger ROS accumulation, lower SOD activity, lower proline/soluble sugar and higher MDA, while KO lines show the opposite within their own background (Figs 2–3).
OE leaves have larger stomatal apertures and lower stomatal density (Fig 4).
A five–time-point drought RNA-seq of OE plants nominates differentially expressed genes in hormone signaling and nitrogen metabolism, including OsPP2C49, OsbZIP23, OsNCED3 and OsRNR10 (Figs 5–6, Figs S2–S3).
OE plants are hypersensitive to exogenous ABA and to the synthesis inhibitor diniconazole, have lower endogenous ABA, and exogenous ABA abolishes the water-loss difference between OE and WT (Fig 7, Fig S4, Tables S1–S2).
Root elongation is positively regulated by OsDUF1223 in a nitrate-concentration-dependent manner (Fig 8).
This is a broad and clearly effortful study, and I appreciate the range of phenotyping the authors have assembled around a gene that genuinely deserves attention. DUF proteins are understudied, and a node that couples nitrogen status to ABA-mediated drought responses would be of interest to many readers. To my mind the nitrate-concentration dependence of the root phenotype (Fig 8G–J) is the most novel single observation in the paper, and within each genetic background the survival and physiology data are internally coherent.
Several of my comments below can be addressed without new experiments. That said, I have three substantive concerns that bear directly on the central message and that I think need to be resolved before the conclusions are defensible: (i) the OE and KO lines are in different genetic backgrounds and the paper's mirror-image "OE up / KO down" logic is never tested against a shared control; (ii) the endogenous-hormone data that anchor the ABA model are unreplicated and internally contradictory; and (iii) the "integration" of ABA and nitrogen signaling is asserted rather than demonstrated. I have numbered my comments to make a point-by-point response easier, and I hope they are useful to the authors and the editor.
Response：We are deeply grateful for your comprehensive and insightful review of this manuscript. We particularly appreciate your accurate and fair assessment of the OsDUF1223 research, especially your recognition of the potential value of this gene as a regulatory node at the intersection of ABA signaling/homeostasis and nitrate-dependent root architecture. Your systematic comments, organized by number, have provided clear guidance for improving the quality of this paper. We fully acknowledge the seriousness of the three critical issues you raised: (i) the overexpression and knockout materials belong to different genetic backgrounds, yet the mirror-image logic of "OE upregulation/KO downregulation" runs throughout the manuscript without a shared control for support; (ii) the endogenous ABA hormone measurements lack biological replicates and contain internal inconsistencies; and (iii) the proposed "integration" of ABA signaling and nitrogen signaling remains at the level of phenotypic correlation and has not yet met the standard for molecular mechanistic evidence. We will provide a thorough point-by-point response to each of these comments.

1.The OE and KO lines are in different genetic backgrounds, and the wild-type values differ enough between them that I am not sure the mirror-image comparison is sound. OE lines are Nipponbare and KO lines are Zhonghua 11 (lines 158–160, 598–602). Almost every conclusion is phrased as "OE does X, KO does the opposite," but the two halves are never measured against the same control — each transgenic set can only be compared to its own WT. The data make me worried this matters in practice. At the same 2 mmol/L NO₃⁻, the Nipponbare WT root length is 5.27 cm while the ZH11 WT is 7.16 cm (lines 420–422). Baseline ABA differs about two-fold between the two WTs (8.05 vs 16.45 ng/g; Tables S1–S2), and baseline JA differs similarly. As in any cross-background comparison, the variation between the two wild types can be as large as the differences the authors attribute to the gene, which makes the "opposite phenotype" framing fragile. I appreciate that the authors flag this in the limitations (lines 572–577), but as written it is a footnote to conclusions that depend on it. The clean solution is same-background loss- and gain-of-function lines, which the authors say are forthcoming (lines 602–604). If those are not feasible on a reasonable timescale, I would ask the authors to recast the Results and Discussion as two independent, within-background experiments, remove the mirror-image phrasing, and restrict each mechanistic claim to the background in which it was observed.
Response：We sincerely thank you for your insightful critique. We fully acknowledge the core concern raised: because the OE materials (Nipponbare background) and KO materials (Zhonghua 11 background) belong to different genetic backgrounds, and their respective wild-type controls exhibit non-negligible differences in multiple baseline parameters, it is logically flawed to directly compare them in a mirror-image manner. Each transgenic material should only be compared with its own background-matched wild-type control, and cross-background descriptions of "opposite phenotypes" may exaggerate or misattribute phenotypic differences to the target gene. To address this, we have thoroughly revised the manuscript, and no such mirror-image comparisons appear in the text. This issue has been addressed in detail in response to Reviewer 2's second comment, with the specific revisions shown on page 9.

2.The endogenous-hormone data are internally contradictory and are reported without replication or statistics. This is the comment I would most like to see addressed, because the "low-content, high-sensitivity" ABA model rests on it.
Direction. In the Nipponbare WT, ABA decreases under drought (Table S2: 8.05 → 5.63 ng/g), whereas in the ZH11 WT it increases (Table S1: 16.45 → 23.2 ng/g). Drought-induced ABA accumulation is one of the most reproducible observations in plant biology, so a wild type that loses ABA under drought is a red flag, and the two wild types cannot both be correct. The JA values behave the same way (WT JA falls under drought in Nipponbare, 3.23 → 1.36, but rises in ZH11, 8.33 → 9.86). Could the authors explain this, and confirm the drought treatment imposed comparable water stress in both experiments?

Response：We fully agree with the your concern that the observed decrease in hormone content in Nipponbare under drought conditions is problematic. To address this, we re-measured the hormone levels in Zhonghua 11 before and after treatment. Based on these results, we conclude that the pre- and post-treatment hormone decline data for Nipponbare are unreliable, due to the following reasons: (1) the plants used for hormone measurement before and after treatment in Nipponbare were from different batches with varying growth conditions; and (2) hormone assays are inherently subject to technical variability. These factors collectively contributed to the anomalous result. Therefore, we have retained only within-group horizontal comparisons and have removed pre- versus post-treatment longitudinal comparisons. Under this premise, despite the limitations of the data, we believe that the within-group comparative results can still serve as supplementary evidence.

3.Replication and statistics. The Methods state only "two technical replicates" with no biological replication for the hormone measurements (lines 700–701), and Tables S1–S2 report single point values with no error and no test. Yet the text repeatedly calls the differences "significant" (e.g., lines 360–364). Please provide at least three biological replicates, report the variance, and test the differences formally. I note this also conflicts with the statement at lines 726–727 that all data have at least three biological replicates.
Inconsistent panels. Table S1 reports ABA/JA/SA/GA3/IAA but Table S2 reports only ABA/JA/SA. Please measure the same panel in both backgrounds.
Response：We sincerely thank you for pointing out the issues regarding biological replicates, statistical presentation, and the hormone assays in the supplementary tables. We apologize for these oversights. Regarding the inconsistency in the hormone types listed in Tables S1 and S2, we would like to clarify that S1 and S2 were not measured simultaneously. The primary purpose of S1 was to conduct a preliminary screening of ABA-related candidate hormones, while S2 was a targeted measurement conducted after identifying the key targets, using SA as a control. To avoid redundant information and to improve the logical clarity of the study, we will remove the GA₃ and IAA data from Table S1. In addition, we will delete the sample size information (e.g., "n=3") from the figure legends and uniformly incorporate this information into the Materials and Methods section to ensure consistent and standardized presentation throughout the manuscript.

4.The transcriptome that anchors the mechanism was generated only in the OE/Nipponbare background. The RNA-seq is OE vs Nipponbare WT only (lines 288–289, 713–714), and the "opposite" molecular response in KO is then asserted from a handful of RT-qPCR genes (Figs 5I, 6D, 8B) rather than shown transcriptome-wide. Because overexpression of a membrane protein is prone to gain-of-function and insertion-site effects, the direction of the key claims (PP2C49, bZIP23, NCED3, nitrogen-metabolism enrichment) would be much more secure with a matched KO/ZH11 RNA-seq at the same time points. Relatedly, the 0 h OE-vs-WT contrast (132 up / 196 down; lines 291–293) is a steady-state genotype difference that is partly attributable to the construct itself, so I would be cautious about interpreting the 0 h "nitrogen metabolism" and "fatty acid elongation" enrichment (Fig S2) as drought mechanism without the KO comparison.
Response：We sincerely thank you for your important and constructive critique. We fully agree that relying solely on RNA-seq data from the OE/Nipponbare background to support the entire molecular mechanism narrative carries a risk of over-interpretation, and that the differences captured at the 0h control time point may partly stem from the insertion effect of the overexpression vector rather than being genuine components of the drought response. As an alternative, we will implement the following revisions: (1) In the Discussion section, we will provide a more cautious interpretation of the "nitrogen metabolism" and other results from the 0h enrichment analysis, explicitly stating that these may reflect genotypic homeostatic background differences rather than direct components of the drought response mechanism; (2) We will downgrade the conclusive statement regarding "opposite molecular responses in KO" to a suggestive description consistent with the OE trend, and clearly note that the current molecular evidence for KO is based solely on a limited set of genes validated by RT-qPCR, which awaits independent validation at the whole-transcriptome level.

*(See the paragraph of Section “Results | Transcriptome Analysis of OsDUF1223 Overexpression Lines in response to Drought Stress”)*

We selected a set of key stress-responsive genes (*OsLOX4*, *OsNR2*, *OsbZIP23*, *OsPAL9*, *LOC_Os1g40860*) for RT-qPCR validation across the transgenic lines. The results showed that the expression trends in the OE lines under the Nipponbare background were consistent with the RNA-seq data, whereas the KO lines under the ZH11 background exhibited opposite trends compared to the RNA-seq data (Fig. 5H–I).

It should be noted that the transcriptomic analysis in this study was conducted exclusively in the Nipponbare overexpression (OE) background. The transcriptome data of knockout (KO) lines in the Zhonghua 11 background have not yet been presented. Therefore, the following descriptions of molecular responses in KO lines are based solely on RT-qPCR validation and have not been systematically confirmed at the transcriptomic level.

*(See the paragraph of Section “Discussion”)*

Transcriptomic analyses provided key insights into the core mechanism: KEGG enrichment analysis showed that at the 0 h time point, upregulated differentially expressed genes in the overexpression lines were already significantly enriched in the "nitrogen metabolism" pathway. This phenomenon may partially reflect genotypic homeostatic background differences, including the influence of overexpression vector insertion effects, and should not be directly attributed to the drought response mechanism.

5.The proposed ABA-signaling logic is internally inconsistent at OsbZIP23. The model holds that OE down-regulates the negative regulator OsPP2C49 to derepress ABA signaling and so become ABA-hypersensitive (lines 471–477, 482–490). But the authors also report OsbZIP23 as down-regulated in OE (validated in Fig 5H–I), and OsbZIP23 is a positive regulator of ABA sensitivity (Xiang et al. 2008, ref 47) that, through feedback, promotes both ABA-responsive gene expression and NCED-dependent biosynthesis (Zong et al. 2016, ref 48). Lower OsbZIP23 would therefore predict reduced ABA sensitivity and lower ABA content. The lower content is consistent with the data, but the reduced sensitivity is the opposite of the hypersensitive phenotype the authors observe. As written, the model selects OsPP2C49 while ignoring that its own upstream regulator moves the wrong way. I would ask the authors to reconcile this, or to acknowledge that the transcriptional changes do not coherently predict the hypersensitivity (which may instead be post-transcriptional).
Response：Thank you for raising this important issue. We fully acknowledge that OsbZIP23 is not only an upstream activator of *OsPP2C49* but also directly activates ABA-responsive gene expression and promotes NCED-dependent ABA biosynthesis, both of which contribute positively to ABA sensitivity. Our initial model, which treated *OsbZIP23* downregulation solely as the cause of *OsPP2C49* downregulation while ignoring its independent positive regulatory functions, was an oversimplification. We now interpret the hypersensitive phenotype as the net outcome of two opposing effects within the same regulatory cascade: the reduction of *OsbZIP23* attenuates ABA biosynthesis and ABA-responsive gene activation (consistent with the reduced ABA content we observed), while the concurrent reduction of its transcriptional target *OsPP2C49* releases the PP2C-mediated inhibition of SnRK2 kinase activity. We propose that the latter effect predominates, because PP2Cs function as gatekeepers that directly inactivate SnRK2s; when the phosphatase brake is released, the dose-response relationship between ABA and signaling output becomes steeper, such that the reduction of PP2C can sensitize the signaling pathway even when ABA ligand levels are moderately reduced. Nevertheless, we agree with the reviewer's point that transcriptional changes alone do not coherently and completely predict the hypersensitive phenotype. Post-transcriptional regulation of *OsbZIP23* protein activity (e.g., via SnRK2-mediated phosphorylation or protein stability) and functional compensation by other bZIP family members may help to resolve the remaining discrepancy. We have revised the working model in the main text accordingly.

*(See the paragraph of Section “Discussion”)*

Transcriptome analysis provided direct molecular evidence for the involvement of *OsDUF1223* in ABA signal transduction. Under drought treatment at 0 h and 1 h, differentially expressed genes in the overexpression lines were significantly enriched in the "stress response" and "hormone signal transduction" pathways, encompassing multiple core ABA signaling components. The heatmap results revealed that the abiotic stress and ABA response factor *OsPP108*, the negative regulator of ABA signaling *OsPP2C49*, and the core positive regulator of ABA signaling *OsbZIP23* were all downregulated in the overexpression lines. PP2C family proteins are core negative regulators of the ABA signaling pathway; their reduced expression relieves the inhibition of downstream SnRK2 kinases, rendering the ABA signaling pathway more prone to activation ^[52]^. Notably, *OsbZIP23* functions not only as a transcriptional activator of *OsPP2C49* but also as a positive regulator of ABA-responsive gene expression and NCED-dependent ABA biosynthesis ^[52, 53]^; its synchronous downregulation with *OsPP2C49* therefore reflects a cause-and-effect relationship within the same regulatory cascade rather than two independent signals. This coordinated reduction exerts opposing effects on ABA signaling. Furthermore, altered expression of JAZ family proteins in the JA pathway implied a complex crosstalk between ABA and JA signals, which together shaped the *OsDUF1223*-mediated stress response network ^[54, 55]^.

This study found that endogenous ABA content in *OsDUF1223* overexpression lines was significantly lower than that in the wild type, yet their sensitivity to exogenous ABA was significantly higher, and they also exhibited stronger growth inhibition in response to treatment with the ABA degradation inhibitor diniconazole. This suggests that *OsDUF1223* exerts a dual regulatory effect on the ABA system. On one hand, within the bZIP23–*OsPP2C49* cascade, although the downregulation of *OsbZIP23* attenuates its positive regulatory function in the activation of ABA-responsive genes, the net phenotype is dominated by the reduction of the terminal negative regulator *OsPP2C49*—because PP2Cs function as gatekeepers that directly dephosphorylate and inactivate SnRK2s; their removal can sensitize the signaling pathway even when ABA ligand levels are moderately reduce. Transcriptional changes alone do not fully account for this outcome; post-transcriptional regulation of *OsbZIP23* protein activity and potential functional compensation by other bZIP family members may further reinforce the hypersensitive phenotype. On the other hand, the downregulation of *OsbZIP23* leads to attenuated NCED-dependent ABA biosynthesis, maintaining endogenous ABA at a low steady-state level. This "low-content, high-sensitivity" regulatory model allows plants to maintain a stress-alert state without accumulating high concentrations of inhibitory hormones, thereby achieving a balance between growth and defense ^[56]^. Exogenous ABA complementation experiments further confirmed that ABA is an essential downstream signaling molecule for *OsDUF1223* in regulating drought tolerance: under combined treatment with 20% PEG and 50 μmol/L ABA, the differences in water loss rate among all lines were eliminated; scanning electron microscopy observations also indicated that the combined treatment induced stomatal closure in the overexpression lines, demonstrating that exogenous ABA could rescue the drought tolerance defect caused by *OsDUF1223* overexpression. Furthermore, differentially expressed genes enriched in the "stress response" pathway included multiple ABA-dependent stress response factors: *OsNAC52* can transcriptionally activate ABA-inducible gene expression ^[57]^, and *OsANN4* can mediate ABA-induced calcium influx and regulate antioxidant enzyme activity ^[58]^. These findings further support the role of *OsDUF1223* in regulating drought tolerance through the ABA pathway.

Based on the above results, we propose the following working model: *OsDUF1223* serves as a potential multifunctional regulatory hub that coordinates the drought response in rice through two synergistic mechanisms. First, *OsDUF1223* integrates drought stress signals by modulating the homeostasis and transduction of the ABA pathway. At the seedling stage, drought-induced *OsDUF1223* expression is accompanied by the downregulation of bZIP23 and its downstream target *OsPP2C49*. Within this cascade, the coordinated downregulation of *OsbZIP23* and *OsPP2C49* governs two dimensions of the ABA system: the reduction of *OsPP2C49* relieves the inhibition of SnRK2 kinases, sensitizing the signaling pathway even under low ABA levels; while the downregulation of *OsbZIP23* attenuates NCED-dependent ABA biosynthesis, maintaining endogenous ABA at a low steady-state level. Together, they establish a "low-content, high-sensitivity" state. In overexpression (OE) lines, the reduced endogenous ABA is insufficient to fully activate downstream drought responses under natural drought conditions, thus requiring exogenous ABA supplementation; conversely, knockout (KO) lines exhibit elevated endogenous ABA levels and enhanced overall drought tolerance, despite reduced signaling sensitivity.

6.I am not sure the diniconazole experiment supports "positive regulation of ABA signaling." OE plants are shorter than WT under exogenous ABA (Fig 7A–B) and also shorter than WT under the ABA-synthesis inhibitor diniconazole "at all concentrations" (lines 384–388, Fig 7E–F). If "at all concentrations" includes the untreated control, then OE is simply shorter at baseline and the diniconazole effect is not ABA-specific. Adding ABA and removing ABA should not move OE height in the same direction relative to WT if the interpretation is a straightforward shift in ABA sensitivity. Could the authors show the control side by side, test the genotype × treatment interaction, and clarify whether the data distinguish "ABA-hypersensitive" from "constitutively shorter"?
Response：We sincerely thank you for your questions. First, regarding your concern that "if 'at all concentrations' includes the untreated control, then the overexpression lines are simply shorter at baseline," we would like to clarify that the description "at all concentrations" in the original manuscript referred exclusively to the treatment groups with the chemical agent and did not include the untreated control. We have revised this phrasing in the updated manuscript to explicitly qualify the statement and avoid ambiguity. According to the data shown in Figure 7F, under untreated conditions, the overexpression lines showed no significant difference in plant height compared to the wild type (NIP). Therefore, the overexpression lines are not constitutively dwarfed and display a normal baseline phenotype. Furthermore, regarding the apparent logical contradiction that "both exogenous ABA addition and ABA reduction via diniconazole result in shorter overexpression lines relative to the wild type, which is inconsistent with a simple shift in ABA sensitivity," we would like to provide further clarification. Diniconazole is a triazole fungicide that not only acts as an ABA biosynthesis inhibitor but also possesses intrinsic phytotoxicity. While it suppresses endogenous ABA synthesis, this compound concurrently exerts toxic effects on the plants, leading to more severe growth inhibition under treatment. This combined effect results in the overexpression lines being relatively shorter than the wild type under "ABA-reduction" conditions, and this outcome cannot be simply interpreted as equivalent to a straightforward shift in sensitivity. For this reason, we consider the diniconazole experiment as only supplementary validation and not as standalone evidence for our conclusions. The core evidence supporting positive regulation of ABA signaling in this study remains based on exogenous ABA treatment and subsequent physiological indicators.

*(See the paragraph of Section “Results | OsDUF1223 positively regulates the ABA signaling pathway, and ABA acts as an essential downstream regulatory signal for it”)*

When two-day-old seedlings were treated with different concentrations of diniconazole (an ABA synthesis inhibitor), the plant height of the overexpression lines was lower than that of Nipponbare at both 10 μmol/L and 20 μmol/L concentrations; in contrast, under the ZH11 background, the knockout lines showed significantly greater plant height than ZH11 only under 20 μmol/L diniconazole treatment.

7.The stomatal explanation is internally muddled, and there is no direct conductance measurement. OE is reported to have both larger aperture and lower density (Fig 4E–I; abstract lines 31–33). These act in opposite directions on transpiration — lower density should reduce water loss — so attributing the higher OE water loss to "stomatal morphology" is not straightforward. A net measure would resolve this: a stomatal pore-area index, or, better, direct gas exchange (porometer/IRGA) giving stomatal conductance, rather than SEM morphology alone. Two smaller points: the SEM is a single critical-point-dried snapshot at 8 h and does not capture closure dynamics, and the legend reports n = 3 while the Methods describe "5 visual fields" per line (lines 686–687), which reads as pseudoreplication. Please clarify the experimental unit and the replication.
Response：We sincerely thank you for this professional and constructive comment. We fully agree with your point that inferring transpirational water loss solely from changes in stomatal size (aperture) and density is not sufficiently straightforward, as the effects of these two parameters on transpiration can be opposite. To address this issue, following your suggestion, we calculated the stomatal pore area index (SPI) as a comprehensive net indicator of stomatal morphology. The newly added SPI data (Figure 4H, K) show that: in the overexpression lines, the SPI was significantly lower than that in Nipponbare; in the knockout lines, the SPI showed no significant difference from that in ZH11. These results clearly indicate that although the overexpression lines exhibited larger stomata (increased guard cell length), the reduction in stomatal density was more pronounced, leading to a lower SPI compared to Nipponbare. However, our water loss rate experiments showed that the overexpression lines had a significantly higher water loss rate than the wild type (Figure 4A, C), suggesting that the increased water loss rate in the overexpression lines is not driven by stomatal morphology (net pore area), but is more likely attributable to altered stomatal movement regulation (e.g., delayed or incomplete stomatal closure under drought stress). We have made corresponding revisions in the Results section and added an analysis of this aspect in the Discussion section. Regarding the issues of experimental replication and experimental units, we have also revised the Materials and Methods section accordingly.

*(See the paragraph of Section “Results | OsDUF1223 affects the water loss rate of rice by regulating the stomatal morphological characteristics of rice leaves”)*

Results showed that, compared with the wild-type Nipponbare, overexpression lines exhibited significantly longer guard cells and significantly lower stomatal density (Fig 4F–G). In contrast, knockout lines displayed significantly shorter guard cells and significantly higher stomatal density compared with the wild-type ZH11 (Fig 4I–J). The stomatal pore-area index (SPI) was calculated as an integrated measure of stomatal morphology (Fig 4H, K). In overexpression lines, the reduction in stomatal density outweighed the effect of increased stomatal size, resulting in a significantly lower SPI value relative to the wild-type Nipponbare. In knockout lines, the SPI value showed no significant difference from that of the wild-type ZH11.

*(See the paragraph of Section “Discussion”)*

Stomatal observations revealed that under drought conditions, overexpression lines exhibited larger stomata but a significantly lower stomatal pore-area index (SPI) compared to Nipponbare, due to a more pronounced reduction in stomatal density. However, leaf water loss rate assays showed that overexpression lines had a significantly higher water loss rate than Nipponbare. This apparent discrepancy between low SPI and high water loss suggests that differences in water loss are not solely determined by static stomatal morphology, but likely reflect alterations in stomatal movement regulation. Previous studies have established that guard cell size is closely linked to closure kinetics: larger guard cells require greater ion flux changes to complete turgor regulation, resulting in significantly slower response rates compared to smaller stomata ^[50, 51]^. Based on these findings, we propose that the elevated water loss rate in overexpression lines may stem from delayed or incomplete stomatal closure under drought stress due to larger guard cells, leading to persistently higher stomatal conductance. Future studies measuring stomatal conductance (gₛ) and stomatal opening/closing kinetics under drought conditions will be essential to further elucidate the specific molecular mechanisms by which *OsDUF1223* regulates stomatal movement and water use efficiency.

*Added reference*:

50.	Hetherington AM, Woodward FI. The role of stomata in sensing and driving environmental change. Nature. 2003;424(6951):901–8.

51.	Lawson T, Blatt MR. Stomatal size, speed, and responsiveness impact on photosynthesis and water use efficiency. Plant Physiol. 2014;164(4):1556–70.

*(See the paragraph of Section “Materials and methods | Observation of Stomatal Morphological Characteristics”)*

For each line, three visual fields were randomly selected for statistical analysis of stomatal aperture and density. The stomatal pore index (SPI), calculated as SPI = SLen² × SD × 10⁻⁴ ^[67]^, serves as an approximate measure of the proportion of stomatal pore area to leaf area and is widely used as an indicator of stomatal opening degree in crop leaves ^[68]^. Here, SD represents stomatal density and SLen represents stomatal length.

*Added reference*:

67.	Liu C, Sack L, Li Y, Zhang J, Yu K, Zhang Q, et al. Relationships of stomatal morphology to the environment across plant communities. Nat Commun. 2023;14(1):6629.

68.	Tian M, Yu G, He N, Hou J. Leaf morphological and anatomical traits from tropical to temperate coniferous forests: Mechanisms and influencing factors. Sci Rep. 2016;6:19703.

8.The "integration" of ABA and nitrogen signaling is asserted but not demonstrated; I would temper the title and the model accordingly. The data show that OE perturbs ABA-associated readouts and, separately, nitrate-dependent root growth. Nothing in the manuscript shows that the two inputs converge at OsDUF1223 — there are no protein interactions, no biochemistry, and no shared downstream target, and the protein is of unknown function. "Integrates ABA and nitrogen signals" (title; abstract lines 35–38; model lines 551–569) therefore reads as stronger than the result. I would suggest a title closer to "OsDUF1223 negatively regulates seedling drought tolerance and modulates nitrate-dependent root growth in rice," and presenting the two-arm figure explicitly as a hypothesis. If the authors wish to keep "integration," it needs an experiment showing convergence.
Response：We fully agree with your comment. The current data are not yet sufficient to provide direct molecular evidence demonstrating an "integration" between these two signaling pathways. In response to this valuable suggestion, we have revised the text accordingly.

*(See the paragraph of Section “Title”)*

*OsDUF1223* negatively regulates seedling drought tolerance and modulates nitrate-dependent root growth in rice

*(See the paragraph of Section “Abstract”)*

Collectively, this study elucidates that *OsDUF1223* regulates drought tolerance at the seedling stage in rice through functional association with the ABA and nitrogen signaling pathways, thereby providing a candidate gene resource for the genetic improvement of drought tolerance in rice.

*(See the paragraph of Section “Discussion”)*

This study provides evidence that *OsDUF1223* may be associated with drought tolerance in rice seedlings through its functional links to ABA signaling and nitrogen metabolism.

9.The nitrogen → root → drought causal chain is weak, and the two nitrate experiments appear to disagree at 2 mmol/L NO₃⁻. The genotype differences in root length disappear under the mild drought (10% PEG) actually used (lines 178, 508–509), which makes it hard to argue that "pre-formed root differences" drive drought outcomes when those differences vanish under the relevant stress. Separately, the two nitrate experiments seem inconsistent: in nitrogen-free Hoagland's with 2 mmol/L NO₃⁻ for 12 d, OE roots are much longer than WT (lines 417–423), whereas in the gradient with a 1 mmol/L glutamine background for 8 d there is no difference at 2 mmol/L NO₃⁻ (lines 430–431). The authors attribute this to the organic-nitrogen background, which is plausible, but the two experiments also differ in duration (12 vs 8 d), so the glutamine effect is confounded with time. A single experiment that varies only the glutamine background at fixed duration and NO₃⁻ would isolate the effect being claimed.
Response：We fully agree with your viewpoint: under 10% PEG treatment, the differences in root length among different genotypes disappeared, making it difficult to support the argument that "pre-formed root differences drive drought phenotypes." In the revised manuscript, we have reframed the core argument, no longer regarding root morphological differences as a decisive factor for drought tolerance, but rather as an indirect manifestation of nitrogen-regulated activity. We further point out that although the phenotypic differences in root length under drought stress disappeared, the upregulation of nitrogen metabolism-related genes in the overexpression lines persisted and was even enhanced (as indicated by KEGG enrichment analysis). We therefore propose that this sustained molecular-level nitrogen regulatory activity—rather than static root morphology—serves as a more direct mechanistic bridge linking *OsDUF1223* to drought tolerance. Regarding the discrepancy in results between the two nitrate experiments at 2 mmol/L NO₃⁻, we agree with your concern about the confounding effects of temporal factors and the glutamine background. In the preliminary experiment, directly exposing very young seedlings to high nitrate concentrations led to unstable growth and high data variability; therefore, we adopted a protocol in which four-day-old seedlings were cultured for 8 days in a medium containing an organic nitrogen background (with the statistical physiological age being 12 days for all treatments). While this approach indeed improved data reproducibility, it did not fully separate the effects of glutamine from those of the culture duration. In the revised manuscript, we have explicitly acknowledged this limitation and described the role of the glutamine background as a "possible factor" rather than a definitive conclusion.

*(See the paragraph of Section “Results | OsDUF1223 affects the drought tolerance of rice by participating in the regulation of root architecture mediated by nitrogen”)*

Moreover, under 2 mmol/L NO₃⁻ and 10 mmol/L NO₃⁻ treatment, there was no significant difference in root length between each transgenic line and the wild type. This may be because glutamine, as a direct product of nitrogen assimilation, can be preferentially utilized by plants, thereby reducing their dependency on exogenous nitrate^[46, 47]^.

*Added reference*:

46.	Svietlova N, Zhyr L, Reichelt M, Grabe V, Mithöfer A. Glutamine as sole nitrogen source prevents induction of nitrate transporter gene NRT2.4 and affects amino acid metabolism in Arabidopsis. Front Plant Sci. 2024;15:1369543.

47.	Pařízková B, Johansson AI, Juvany M, Šimura J, Ljung K, Antoniadi I. L-Glutamine Modulates Root Architecture and Hormonal Balance in Arabidopsis. Physiol Plant. 2026;178(1):e70723.

10.The OsRNR10 result contradicts the cited literature and is resolved only by assertion. The authors note that OsRNR10 (Huang et al. 2023, Nature Plants; ref 56) inhibits NO₃⁻ uptake and root development through auxin, yet here OsRNR10 is up-regulated in OE while roots are longer (lines 531–535). Describing this as a "complex regulatory network or compensatory mechanism" (lines 534–536) restates the contradiction rather than resolving it. As written this is a weakness in the nitrogen arm rather than support for it. Could the authors provide evidence for the proposed compensation (for example auxin measurements, or an OsRNR10-dependence test), or present it explicitly as an unresolved discrepancy?
Response：Thank you for raising this point. We fully agree with your assessment: the original manuscript's description of the contradiction between *OsRNR10* upregulation and root elongation in OE lines was clearly flawed, and the phrasing "complex regulatory network or compensatory mechanism" merely restated the contradiction rather than explaining it. In the revised manuscript, we have deleted the relevant paragraphs that claimed this contradiction could be explained by a complex network, and no longer present it as part of the mechanistic chain of the nitrogen metabolism pathway. Instead, we have explicitly designated this contradiction as an unresolved open question, noting that it may reflect functional plasticity of *OsRNR10* across different genetic backgrounds or stress conditions, or may suggest that *OsDUF1223* functionally antagonizes *OsRNR10* through yet-to-be-identified downstream factors—an issue that requires future auxin phenotypic analyses and genetic interaction experiments between *OsRNR10* and *OsDUF1223* for validation. Meanwhile, we have clearly distinguished the nitrate concentration gradient experiments from the *OsRNR10* contradiction. The former merely reflects the dependence of the *OsDUF1223* root phenotype on nitrogen supply and is unrelated to the contradiction between *OsRNR10* upregulation and root elongation; the revised manuscript avoids using the former to explain the latter. We believe that explicitly acknowledging an unresolved contradiction and pointing out future research directions is more honest than providing a speculative explanation lacking evidence, and is more consistent with your recommendation.

*(See the paragraph of Section “Discussion”)*

Among the overlapping DEGs consistently upregulated across all time points, *OsRNR10* is known to inhibit NO₃⁻ uptake and root development by modulating auxin accumulation ^[59]^. Theoretically, *OsRNR10* upregulation should suppress root growth; however, OE lines exhibited elongated roots. This contradiction has not been mechanistically resolved in the present study; it may reflect functional plasticity of *OsRNR10* across different genetic backgrounds or stress conditions, or may suggest that *OsDUF1223* functionally antagonizes *OsRNR10* through yet-to-be-identified downstream factors. Resolving this contradiction will require further auxin phenotypic analyses and genetic interaction experiments between *OsRNR10* and *OsDUF1223*. Nitrate concentration gradient experiments demonstrated that the root phenotype effects of *OsDUF1223* are dependent on nitrogen availability: under nitrogen-free or low-nitrogen conditions, OE lines exhibited significantly shorter roots than wild-type plants, whereas no difference was observed under high-nitrogen conditions. Conversely, KO lines in the ZH11 background showed the opposite trend, suggesting that *OsDUF1223*-mediated root developmental regulation is closely linked to nitrogen signaling pathways. These findings reveal an association between nitrogen signaling and root development but do not resolve the phenotypic contradiction between *OsRNR10* upregulation and root elongation in OE lines, an issue that awaits future elucidation.

*Added reference*:

59.	Huang Y, Ji Z, Tao Y, Wei S, Jiao W, Fang Y, et al. Improving rice nitrogen-use efficiency by modulating a novel monouniquitination machinery for optimal root plasticity response to nitrogen. Nat Plants. 2023;9(11):1902–14.

11. No complementation, allelism, or genetic-interaction test. To place OsDUF1223 in the ABA and nitrate pathways it is associated with, it would help to go beyond OE vs KO. A complementation/rescue of the KO (re-introducing OsDUF1223 into the ZH11 background and recovering the OE-like phenotype) would also control for CRISPR off-target effects. More informative still would be a genetic-interaction test — for example, whether the OE drought phenotype requires ABA signaling (in an ospp2c49 or osbzip23 background) and whether the root phenotype requires OsRNR10. I recognize these are non-trivial to generate; I raise them because the causal architecture in the model (lines 551–569) is currently inferred entirely from expression correlations and pharmacology.
Response：We fully agree with your comment. This is indeed an important limitation of our current study. In the revised manuscript, we have adjusted the wording of the causal statements regarding the pathway assignment of *OsDUF1223* in the Results section. Additionally, a dedicated limitations paragraph has been added to the Discussion section, which clearly states that the causal inference of the current model is primarily based on expression co-regulation networks and pharmacological perturbation experiments, and that direct genetic validation through functional complementation experiments (complementing *OsDUF1223* in the ZH11 background to restore the OE phenotype) and genetic interaction experiments (validating phenotype dependency in *ospp2c49*, *osbzip23*, or *OsRNR10* mutant backgrounds) is still lacking. These experiments have been included in our future research plans.

*(See the paragraph of Section “Results | Determination of Drought Tolerance Indicators in OsDUF1223 Transgenic Plants”)*

In summary, under drought stress, overexpression of *OsDUF1223* in rice seedlings is accompanied by reduced ROS scavenging capacity, decreased antioxidant enzyme activity, insufficient accumulation of osmotic adjustment substances, and aggravated membrane lipid peroxidation, while knockout lines in the ZH11 background show the opposite trends. These changes in physiological and biochemical indicators suggest that the expression level of *OsDUF1223* is negatively correlated with drought tolerance in rice seedlings. However, its regulatory role in drought tolerance still requires further validation through functional complementation and genetic interaction experiments.

*(See the paragraph of Section “Discussion”)*

By integrating these pathways, we have preliminarily elucidated a potential molecular mechanism underlying *OsDUF1223*-mediated drought tolerance in rice seedlings, although several limitations remain. First, transgenic materials were generated in different genetic backgrounds (Nipponbare for overexpression and ZH11 for knockout), which may interfere with direct phenotypic comparisons. Additionally, the current transcriptomic data only reflect transcriptional regulatory effects of *OsDUF1223* under the overexpression background and cannot fully exclude background-related confounding factors. Second, the direct interaction between *OsDUF1223* and core components of the ABA signaling pathway remains unclear; the crosstalk network between nitrogen and ABA signals, as well as the hormonal mechanisms regulating root plasticity, have yet to be resolved. Third, the current consistency among phenotypic, physiological, and gene expression data constitutes an indirect line of evidence supporting the negative regulation of drought tolerance by *OsDUF1223*, but has not yet reached the level of rigorous genetic causal validation—the possibility of CRISPR off-target effects in the OE and KO lines, as well as whether *OsDUF1223* mediates its drought phenotype through known ABA signaling components such as *OsPP2C49* or *OsbZIP23*, both require further confirmation. Fourth, this study focused solely on the seedling stage; the functions of *OsDUF1223* at the adult stage and under field drought conditions, as well as its potential impacts on agronomic traits, remain to be verified.

12.CRISPR knockout controls. Only the 1-bp frameshift deletion is described (lines 160–162). Please report whether the KO lines are Cas9-free (transgene-segregated), an off-target assessment, and confirmation that the frameshift produces a true null (loss of transcript or protein), since the entire loss-of-function argument rests on it.
Response：We sincerely thank you for your detailed comments. Our responses to the three specific issues raised—Cas9 segregation, off-target assessment, and null phenotype confirmation—are as follows. First, the knockout lines were generated using a transient Cas9 expression system and have been propagated for multiple generations without selection pressure after editing, which inherently promotes the loss of the Cas9 transgene. Formal PCR detection of residual Cas9 transgene has not yet been completed; to further validate, independent knockout lines and a complementation line are being generated in the Nipponbare background. Second, the gRNA used in this study was designed using the CRISPR-P2.0 tool, with careful consideration of target specificity; the assessment results indicate an extremely low probability of off-target effects. Third, PCR sequencing confirmed the presence of a homozygous 1-bp frameshift deletion at the target site, introducing a premature stop codon (Supplementary Figure S1D) that truncates the protein before the critical functional domain. We fully agree with the reviewer's point that genotype and sequence analysis alone are insufficient to fully prove a null state, and we deeply regret this shortcoming, which has been highlighted in the Discussion section. In summary, although this study falls short of completing all idealized characterizations, the precise genotype confirmation, predicted protein truncation, T3 generation stable genotype, and consistent drought-sensitive phenotype together provide an acceptable level of evidence for the loss-of-function interpretation under current conditions.

The prediction results of CRISPR-P 2.0 are shown in the figure below:

*(See the paragraph of Section “Results | Generation of OsDUF1223 Transgenic Plants and Subcellular Localization of Its Protein”)*

The sequencing results showed that all knockout lines carried a 1 bp deletion at the target site, resulting in a frameshift mutation of *OsDUF1223* and leading to premature translation termination.

Note: C. Mutation sequences of the *OsDUF1223* knockout lines, showing only the region flanking the editing site (257–282 bp); identical sequences within this region are omitted. The number of deleted bases is indicated by a minus sign (–) followed by the number in square brackets. D. Alignment of the deduced amino acid sequences of the wild type (ZH11) and *OsDUF1223* knockout lines around the mutation site; identical amino acid residues are not shown, and the yellow box indicates the position where the frameshift mutation begins. * represents the stop codon induced by the frameshift mutation.

13. Does the OE dosage track the phenotype? OE-4 appears to be the strongest expresser (Fig S1A), but the three-leaf survival rates (OE-1 29.9%, OE-4 33.4%, OE-5 41.7%; lines 184–185) do not obviously follow expression level. A dosage–phenotype correlation would strengthen the OE conclusions; if it is absent, a comment on why would be useful.
Response：Thank you for your meticulous and critical observation. Among the three overexpression lines, there was no strict linear correlation between the transgene expression level and the leaf survival rate of triploid plants. We attribute this to a possible threshold/saturation effect: once overexpression exceeds a certain critical threshold, the protective phenotype is maximally activated, and further increases in mRNA abundance no longer confer additional phenotypic gains. Under this model, the differences in survival rates among the three overexpression lines may reflect the influence of post-transcriptional or site-specific factors (such as chromatin environment, mRNA stability, etc.), rather than a strict dose–response relationship.

14.Survival-assay design. Within the three-leaf experiment, OE lines were rewatered after 3 d of stress but KO lines after 4 d (lines 650–651). Applying a different stress duration to each genotype before scoring survival builds the expected direction into the protocol and makes the OE-vs-KO survival comparison hard to interpret as a fair one. Could the authors justify this, and where possible report survival under matched stress? The four-leaf assay uses a very different regime (30 d of withheld water, 17 d recovery; lines 654–657) — please confirm "30 d" is correct, and define what constitutes a "biological replicate" for survival (a pot? how many plants?), since the precise survival percentages imply specific denominators.
Response：Thank you for your important observation. The difference in treatment duration in the three-leaf-stage experiment stemmed from the distinct basal drought tolerance levels of the two wild-type varieties, Zhonghua 11 and Nipponbare. Accordingly, we determined the appropriate treatment duration for each: Nipponbare is relatively sensitive to drought, and significant phenotypic differences between genotypes could be observed after 3 days of treatment; whereas Zhonghua 11 is more tolerant, requiring 4 days of treatment to clearly distinguish the response differences among genotypes. Therefore, the treatment duration for the OE lines and their controls was 3 days, while that for the KO lines and their controls was 4 days. Regarding the four-leaf-stage experiment, we confirm that the drought treatment duration of "30 days without watering" is accurate. This duration was determined based on the characteristics of four-leaf-stage plants, which have larger leaf areas and relatively lower transpiration rates. We recorded soil moisture content during the formal experiment to ensure that all tested plants had reached a severe and consistent wilting state by the end of the 30-day period. We also confirm the "17-day recovery period." The longer recovery period was set to ensure that all surviving plants could fully restore their growth status (e.g., new leaf expansion, restoration of upright posture), thereby allowing a more accurate determination of "survival" rather than simply "surviving but not yet recovered." Regarding the definition of biological replicates: in this study, one biological replicate is defined as "one pot." In the four-leaf-stage experiment, each genotype contained 3 biological replicate pots, with 16 plants per pot (48 plants in total).

15.Statistical treatment. As in the hormone tables, multi-group comparisons (WT vs OE-1/-4/-5; WT vs KO-1/-2) are analyzed by ANOVA in some figures and by what appear to be pairwise tests in others, with no stated post-hoc correction (lines 722–727). Across three or more genotypes, uncorrected pairwise t-tests inflate the type-I error rate; please standardize to ANOVA with an appropriate correction (Tukey/Dunnett) and state it. For the survival data analyzed by one-way ANOVA on percentages (Fig 2H, J), please justify the test or use a model appropriate to proportions. For the RNA-seq, the DEG threshold is given inconsistently — "FC ≥ 2 / ≤ 0.5" (lines 290–291) vs "|FC| ≥ 2, P < 0.05" (lines 718–719) — and it is not clear whether the P-value is the DESeq2 adjusted P (FDR); please report the FDR used throughout, as a raw P < 0.05 is not an acceptable transcriptome-wide cutoff.
Response：We sincerely thank you for your careful review of the statistical analyses. First, although the figure legends already indicated that multiple comparison tests were performed for the multi-group comparisons (WT vs. OE-1/-4/-5; WT vs. KO-1/-2), the correction method was not explicitly stated. We have now revised all figures to consistently use one-way ANOVA followed by Tukey HSD post-hoc tests, and the testing method has been clearly specified in the revised figure legends. Second, regarding the biological replicate information in the figure legends, we have removed such details from the legends, and the relevant sample size information is now uniformly described in the Materials and Methods section. Third, for the survival percentage data, we confirmed that the data for each genotype passed the normality test, and that one-way ANOVA with Tukey's test provides sufficient statistical robustness under these conditions. Finally, regarding the inconsistency in the threshold description for RNA-seq differential expression, the "P < 0.05" was a typographical error. We have now unified the standard as described in the Results section: |log2FC| ≥ 1 (i.e., FC ≥ 2 or ≤ 0.5) with DESeq2-corrected FDR < 0.05, and this expression has been corrected consistently throughout the manuscript.

*(See the paragraph of Section “Materials and methods | Transcriptome Analysis of OsDUF1223 Overexpression Lines in response to Drought Stress”)*

Gene expression levels were normalized using FPKM (Fragments Per Kilobase per Million reads) and visualized as a heatmap. Differential expression analysis was performed using raw read counts as input, with median-of-ratios normalization, fold change calculation, and Wald test based on negative binomial distribution implemented in DESeq2. Differentially expressed genes were identified within the set of protein-coding genes using the thresholds of |log₂FC| ≥ 1 and FDR < 0.05 ^[70]^.

*Added reference*:

70.	Love MI, Huber W, Anders S. Moderated estimation of fold change and dispersion for RNA-seq data with DESeq2. Genome Biol. 2014;15(12):550.

*(See the paragraph of Section “Results | Transcriptome Analysis of OsDUF1223 Overexpression Lines in response to Drought Stress”)*

Differentially expressed genes (DEGs) were identified using a threshold of |log₂(fold change)| ≥ 1 and FDR < 0.05.

16.Subcellular localization and baseline physiology. The plasma-membrane assignment rests on transient N. benthamiana co-expression with a PM-RFP marker (lines 162–167). For a 274-aa protein, it would help to report the predicted topology (transmembrane domains? signal/anchor?), whether the protein is integral or peripheral, and ideally to confirm localization in a rice system (protoplasts or stable lines). Separately, soluble-sugar content is already higher in some OE lines than WT under normal conditions (lines 232–233), which complicates the drought interpretation and is worth a comment. The introduction emphasizes both SOD and POD (lines 86–88) but only SOD is measured; adding POD/CAT, and quantifying the DAB/NBT staining (Fig 3A–B) rather than describing it qualitatively, would round out the antioxidant claim.
Response：We appreciate your raising this question. Regarding the topological structure of *OsDUF1223*, SignalP 6.0 predicted no N/C-terminal signal peptide; TMHMM 2.0 predicted no transmembrane helices, and the protein exhibited an overall hydrophilic conformation (outside probability ≈ 1), consistent with the topological features of a peripherally membrane-associated soluble protein rather than an integral membrane protein. In this study, the plasma membrane localization of the target protein (274 aa) was determined based on co-expression experiments with the PM-RFP marker protein in *N. benthamiana*. The fluorescence signals of the two proteins showed a high degree of overlap under confocal microscopy, and the co-localization coefficient (Pearson's Coefficient) between the target protein signal and the RFP plasma membrane signal reached 0.835, providing direct biophysical evidence for plasma membrane localization. *N. benthamiana*, as a classic transient expression system in plant molecular biology, has cell wall structures and membrane protein sorting mechanisms that are highly conserved with those of monocotyledonous plants such as rice. Therefore, we consider that the current experimental results are sufficient to support the conclusion of plasma membrane localization of this protein, without the need for additional validation in protoplasts or stably transformed rice materials. Regarding the soluble sugar content, this dynamic change reveals a key physiological logic: although the OE lines had higher soluble sugar content before treatment, they were more sensitive to drought stress, and their soluble sugar levels dropped sharply after drought treatment—suggesting excessive consumption or impaired homeostasis maintenance of these osmoprotectants during stress response. In contrast, the KO lines in the ZH11 background retained higher levels of both compounds in the later stages of treatment, consistent with their stronger drought tolerance. These data indicate that loss of function of *OsDUF1223* enhances drought tolerance by reducing the excessive mobilization of soluble sugars and proline under drought conditions, whereas overexpression promotes excessive mobilization of carbon and nitrogen metabolites under stress, leading to metabolic homeostasis imbalance and drought sensitivity. Regarding antioxidant enzymes, since only SOD activity was measured in this study, the description of POD in the introduction has been removed; the DAB/NBT staining results have been revised from qualitative to semi-quantitative descriptions, with the addition of staining area measurement data to support the conclusion of enhanced antioxidant activity.

*(See the paragraph of Section “Results | Generation of OsDUF1223 Transgenic Plants and Subcellular Localization of Its Protein”)*

To investigate the subcellular localization of the *OsDUF1223* protein, we first performed topological structure prediction. SignalP 6.0 analysis indicated that the protein lacks an N-terminal or C-terminal signal peptide; TMHMM 2.0 predicted that it contains no transmembrane helices and exhibits a predominantly hydrophilic conformation (Fig S2). This topological feature is consistent with peripheral membrane-associated soluble proteins, distinct from integral membrane proteins, suggesting that *OsDUF1223* may localize to the plasma membrane in a non-transmembrane manner. To this end, we conducted co-localization assays in Nicotiana benthamiana epidermal cells by co-expressing 35S:*OsDUF1223*-GFP with the plasma membrane marker *AtPIP2A*-mCherry. As shown in Fig 1F, under confocal microscopy, the *OsDUF1223*-GFP signal (green) and the *AtPIP2A*-mCherry signal (red) exhibited substantial overlap and significant co-localization at the plasma membrane. Quantitative co-localization analysis (Table S1) revealed a Pearson's correlation coefficient of 0.835, with Manders' coefficients of M1 = 0.809 (GFP overlapping with RFP) and M2 = 0.802 (RFP overlapping with GFP). The topological predictions and co-localization experimental results corroborate each other, collectively demonstrating that *OsDUF1223* localizes to the plasma membrane as a peripheral membrane-associated soluble protein, rather than being embedded in the lipid bilayer as an integral membrane protein.

**Fig S2 *OsDUF1223* transmembrane structure and signal peptide prediction**

Note: A. TMHMM transmembrane structure prediction; B. SignalP signal peptide prediction.

**Table S1 The results of quantitative co-localization analysis**

| Group | Pearson’s Coefficient | M1 | M2 |
| --- | --- | --- | --- |
| 35S:GFP | 0.778 | 0.753 | 0.606 |
| 35S:*OsDUF1223*-GFP | 0.835 | 0.809 | 0.802 |

*(See the paragraph of Section “Introduction”)*

The dynamic changes in soluble sugar content reveal a key physiological logic: although the overexpression lines exhibited higher soluble sugar levels prior to treatment, they were more sensitive to drought stress, with soluble sugar levels declining sharply after drought treatment—suggesting that these osmoprotective substances were excessively consumed during stress response, or that the capacity for homeostatic maintenance was impaired ^[48]^. In contrast, the knockout lines in the ZH11 genetic background maintained relatively high levels of both compounds (soluble sugars and proline) during the later stages of treatment, consistent with their enhanced drought tolerance. These data indicate that loss of *OsDUF1223* function enhances drought tolerance by reducing the excessive mobilization of soluble sugars and proline under drought conditions; conversely, overexpression promotes excessive mobilization of carbon and nitrogen metabolites under stress, leading to metabolic homeostasis imbalance and consequently drought sensitivity ^[49]^.

*Added reference*:

48.	Urmi TA, Islam MM, Zumur KN, Abedin MA, Haque MM, Siddiqui MH, et al. Combined Effect of Salicylic Acid and Proline Mitigates Drought Stress in Rice (Oryza sativa L.) through the Modulation of Physiological Attributes and Antioxidant Enzymes. Antioxidants (Basel). 2023;12(7):1438.

49.	Cai S, Jiang G, Ye N, Chu Z, Xu X, Zhang J, et al. A key ABA catabolic gene, OsABA8ox3, is involved in drought stress resistance in rice. PLoS One. 2015;10(2):e0116646.

*(See the paragraph of Section “Introduction”)*

Plants eliminate excess ROS via enzymatic antioxidant systems such as superoxide dismutase (SOD), alleviating subsequent cellular damage ^[26, 27]^.

*(See the paragraph of Section “Results | Determination of Drought Tolerance Indicators in OsDUF1223 Transgenic Plants”)*

To objectively evaluate the staining results, we further performed semi-quantitative analysis of the DAB and NBT staining signals (Fig S4). The results showed that after drought treatment, the relative accumulation levels of H₂O₂ and O₂⁻ in the overexpression lines were significantly higher than those in Nipponbare; conversely, the knockout lines in the ZH11 background exhibited significantly lower levels than ZH11, which was fully consistent with the staining phenotypes. Together, these qualitative and quantitative results indicate that *OsDUF1223* may negatively regulate the plant's ROS scavenging capacity.

**Fig S4 Semi-quantitative analysis of NBT and DAB staining in leaves of *OsDUF1223* transgenic rice lines.**

Note: ROS were semi-quantitatively measured as %-area fraction. (A, B) Semi-quantitative analysis of NBT staining: (A) Overexpression (OE) lines; (B) Knockout (KO) lines. (C, D) Semi-quantitative analysis of DAB staining: (C) Overexpression (OE) lines; (D) Knockout (KO) lines. Data are presented as mean ± SEM and were analyzed by two-way ANOVA with Tukey's multiple comparison test. ** indicates a highly significant difference (*P* < 0.01).

17.Please make sure the references are accurately cited. At least two appear mismatched to their in-text claims and look like reference-manager errors: ref 7 (Maréchal & Brisson 2010, New Phytologist, on organelle-genome recombination) is cited for "ABA accumulation in mesophyll cells effectively improves drought resistance" (lines 57–58), and ref 57 (Buckley 2019, "How do stomata respond to water status?", New Phytologist) is cited for the "modified Yoshida nutrient solution" recipe (line 607), where the standard citation is Yoshida et al. 1976. I would recommend checking the whole bibliography against in-text usage. Also, the qPCR reference gene is named "Ubiquitin (Os01g0328400)" in the text (line 619) but "UBQ5" in Table S3 — please reconcile.

Response：Thank you very much for your careful verification of the reference genes. The issues you pointed out indeed exist, and these were all errors generated during the literature management process. We have corrected them one by one. Regarding the inconsistency in the name of the qPCR reference gene, in fact, Os01g0328400 and UBQ5 are the same gene. To maintain consistency throughout the manuscript, we have uniformly used Ubiquitin 5 (Os01g0328400) as the standard name for this reference gene in the main text and Table S3.

*(See the paragraph of Section “Introduction”)*

Studies have demonstrated that ABA accumulation in mesophyll cells effectively improves drought resistance ^[7]^.

*(See the paragraph of Section “Materials and methods | Plant Material and Growth Conditions”)*

Seeds were soaked in 3% H₂O₂ solution for 30 min for disinfection, followed by rinsing with sterile water and germination in the dark. Rice seedlings were then cultured in a growth chamber with modified Yosida nutrient solution^[62]^, and grown to the three-leaf stage under the conditions of 16 h light (28°C) and 8 h dark (25°C).

*Added reference*:

7.	McAdam SAM, Brodribb TJ. Mesophyll Cells Are the Main Site of Abscisic Acid Biosynthesis in Water-Stressed Leaves. Plant Physiol. 2018;177(3):911–7.

62.	Yoshida SF, Douglas A.; Cock, James H. Laboratory manual for physiological studies of rice: International Rice Research Institute; 1971.

*(See the paragraph of Section “Materials and methods | RNA extraction and real-time qPCR analysis”)*

RT-qPCR was performed with SYBR Green PCR Master Mix (ABclonal) on a real-time PCR instrument, using Ubiquitin 5 (Os01g0328400) as the internal reference gene. All primer sequences are listed in Supplementary Table S4.

18.Data availability. I could not find an accession for the authors' own RNA-seq (processed by OE Biotech / OECloud); the public data retrieved from the GSA (lines 590–591) are separate. Please deposit the newly generated reads (GSA/SRA/GEO) and provide the accession, and confirm deposition of the supplementary tables.
Response：Thank you very much for this important and meticulous comment regarding data availability. The raw sequence data reported in this paper have been deposited in the Genome Sequence Archive (Genomics, Proteomics & Bioinformatics 2025) in National Genomics Data Center (Nucleic Acids Res 2025), China National Center for Bioinformation / Beijing Institute of Genomics, Chinese Academy of Sciences (GSA: CRA046687) that are publicly accessible at https://ngdc.cncb.ac.cn/gsa.

19.A suggestion, not a requirement. The study is restricted to the seedling stage. It would strengthen the work, and the breeding framing in particular, to know whether the drought phenotype carries through to the adult plant and the field, and whether it has agronomic-trait costs. I appreciate this would take time and may be beyond the scope of the present manuscript; I raise it because the abstract and discussion lean on the breeding application (lines 37–38, 583–586).

Response：Thank you for your valuable suggestion. We fully agree with your point — the current study is limited to the seedling stage, and incorporating the continuity of drought phenotypes into the adult stage and field conditions, combined with evaluating potential agronomic traits, will significantly enhance the persuasiveness of this study for breeding applications. Given that the core objective of this study is to screen and preliminarily validate the phenotypic effects of drought-tolerant candidate genes/loci under drought stress at the seedling stage, we understand that extending the research to adult-stage and whole-growth-cycle field trials requires additional time and resource investment. Therefore, we have planned this as a future work direction. In response to your suggestion, we have made the following adjustments in the revised manuscript.

*(See the paragraph of Section “Abstract”)*

Collectively, this study elucidates that *OsDUF1223* regulates drought tolerance at the seedling stage in rice through functional association with the ABA and nitrogen signaling pathways, thereby providing a candidate gene resource for the genetic improvement of drought tolerance in rice.

*(See the paragraph of Section “Discussion”)*

Future work will be dedicated to further refining the mechanistic understanding through targeted experiments, thereby establishing a more solid theoretical foundation for in-depth dissection of the molecular regulatory networks underlying crop stress tolerance.
