# Supplementary material for: OsDUF1223 negatively regulates seedling drought tolerance and modulates nitrate-dependent root growth in rice
Source: PLoS Genet. 2026 Sep 3;22(9):e1012295. doi: 10.1371/journal.pgen.1012295 (PMC13541127; doi:10.1371/journal.pgen.1012295)
Supplement: Supplementary file 2 [file pgen.1012295.s017.docx]

**Response Letter to Reviewers’ Comments**

**Comments from the Editor**:

Reviewers agree that the manuscript has been substantially improved and that the key scientific concerns raised previously have been addressed. The remaining comments focus primarily on clarification of conclusions and methodological reporting rather than new experimentation. The authors should revise the text to avoid overstating mechanistic conclusions, address the remaining logical inconsistencies highlighted by the reviewers, provide the requested clarifications regarding Figure 1 and transgenic line comparisons, and ensure that experimental design information (including sample sizes and replication) remains clearly presented and easily accessible to readers.

**Response**: We sincerely thank all editors and reviewers for their valuable comments and suggestions on our manuscript. We are pleased that the reviewers acknowledge the manuscript has been substantially improved and that the key scientific concerns raised previously have been addressed. In response to the further comments regarding the conclusions and methodological reporting, we have carefully adopted all suggestions and made comprehensive revisions to the manuscript to enhance its clarity and facilitate readers' understanding: we have cautiously revised the wording of mechanistic conclusions to avoid overinterpretation, identified and corrected the remaining logical inconsistencies, provided the requested clarifications regarding Figure 1 and the comparison of transgenic lines, and ensured that experimental design information, including sample sizes and replication, is clearly presented and easily accessible. Our point-to-point responses are presented below, with the revised content marked in blue. We hope that these revisions satisfactorily address the comments and concerns of the editors and reviewers.

Reviewer #1:

The authors have adequately addressed my previous comments. Regarding the number of biological replicates, I recognize that my previous comment did not fully take into account the experimental design described by the authors, and I have no further concerns regarding this point.

Regarding the microarray data, although it is unfortunate that the original dataset is no longer available, I understand and accept the authors’ explanation.

The authors have also adequately addressed my concerns regarding the phylogenetic analysis of OsDUF1223 and the differences in root phenotypes under the various nitrogen conditions.

Overall, the manuscript has been appropriately revised, and I have no further major concerns.

**Response**: We sincerely thank the reviewer for taking the valuable time to re-review the revised manuscript and for providing such positive feedback. We are very pleased that our revisions have met with your approval. The reviewer's comments and suggestions have been highly constructive and have significantly improved the quality of the manuscript. Please accept our most sincere gratitude!

Reviewer #2:

1. Supplementary optimization of Figure 1-related details and phenotypic evidence

The title of Figure 1 is inaccurate and should specify the OsDUF1223 protein subcellular localization rather than the gene. The plasma membrane RFP marker used in Figure 1F lacks source and methodological description, which should be supplemented for reproducibility. In addition, the claimed “perfect overlap” between GFP and RFP signals is not fully supported by the merged image. The authors should provide magnified views or simple colocalization quantification (e.g., Pearson correlation coefficient) to solidify the subcellular localization results.

**Response**: We sincerely thank the reviewer for the valuable comments on Figure 1. We have revised the manuscript accordingly: the title of Figure 1 has been modified to "Expression pattern analysis of the *OsDUF1223* gene and subcellular localization of its protein" to accurately reflect that the presented results represent protein-level localization rather than gene localization; the source and detailed methodological description of the plasma membrane RFP marker have been supplemented in the Materials and Methods section to ensure experimental reproducibility; additionally, a quantitative colocalization analysis (Pearson's correlation coefficient) of the GFP and *AtPIP2A*-mCherry signals in Figure 1F has been performed to support the colocalization of the two signals with quantitative data, and the description of "complete overlap" in the main text has been revised accordingly to an accurate description based on the quantitative data, avoiding overstatement. All the above revisions have been incorporated into the revised manuscript.

*(See the paragraph of Section “Materials and methods | Subcellular localization of OsDUF1223 protein”)*

Meanwhile, the coding sequence of the *Arabidopsis thaliana* plasma membrane aquaporin gene *AtPIP2A* was cloned into the pK7m34GW vector to generate the *AtPIP2A*-mCherry fusion construct^[65]^, which served as a plasma membrane marker.

*(See the paragraph of Section “Results | Generation and Subcellular Localization of OsDUF1223 Transgenic Plants”)*

To this end, we conducted co-localization assays in *Nicotiana benthamiana* epidermal cells by co-expressing 35S:*OsDUF1223*-GFP with the plasma membrane marker *AtPIP2A*-mCherry. As shown in Fig 1F, under confocal microscopy, the *OsDUF1223*-GFP signal (green) and the *AtPIP2A*-mCherry signal (red) exhibited substantial overlap and significant co-localization at the plasma membrane. Quantitative co-localization analysis (Table S1) revealed a Pearson's correlation coefficient of 0.835, with Manders' coefficients of M1 = 0.809 (GFP overlapping with RFP) and M2 = 0.802 (RFP overlapping with GFP).

**Table S1 The results of quantitative co-localization analysis**

| Group | Pearson’s Coefficient | M1 | M2 |
| --- | --- | --- | --- |
| 35S:GFP(Figure 1F) | 0.778 | 0.753 | 0.606 |
| 35S:*OsDUF1223*-GFP(Figure 1F) | 0.835 | 0.809 | 0.802 |

*Previous Figure 1*

*Modified Figure 1*

2. Standardization of transgenic material background and unified interpretation of phenotypic data

OE and KO lines were generated in Nipponbare and Zhonghua 11 backgrounds, respectively. Although both are japonica rice, intrinsic genetic background differences preclude direct phenotypic comparison between OE and KO lines; each transgenic line should only be compared with its corresponding wild-type control. The background-dependent drought variation between the two cultivars should be explicitly discussed. Furthermore, the inconsistent drought treatment duration (3 d for OE, 4 d for KO) needs a clear methodological explanation to justify the experimental rationality and result reliability.

**Response**: Thank you for your suggestions. In the revised manuscript, we have systematically implemented the "same-background comparison" principle and provided further explanation regarding the methodological basis and data reliability of this principle. First, we have clearly stated throughout the manuscript (including figure legends, Results, and Discussion) that all transgenic lines are statistically compared only with their corresponding wild-type controls; any observations involving cross-background comparisons are presented solely as qualitative trends for reference without quantitative comparison, and all conclusions are drawn based on relative differences within the same background. Additionally, following your suggestion, we have added a discussion in the Discussion section regarding the inherent drought tolerance differences between the two varieties: in Figure 2G–J, the baseline survival rates of Nipponbare and Zhonghua 11 under the same treatment are clearly distinct, indicating intrinsic varietal differences in drought tolerance (Zhonghua 11 is relatively drought-tolerant, while Nipponbare is relatively sensitive). This baseline difference represents a background effect inherent to the two genetic backgrounds and precisely underscores why same-background comparisons must be used, and why direct cross-background comparisons between OE and KO phenotypes are not appropriate. Second, regarding the issue of inconsistent treatment duration (3 days for OE lines, 4 days for KO lines), we have added a clear methodological explanation: the endpoint of treatment in this study was determined not by "treatment days" but by the "stress intensity reached in each background." Because Nipponbare is relatively drought-sensitive, 3 days of treatment was sufficient to reach a moderate stress level and fully reveal genotypic differences; in contrast, Zhonghua 11 is relatively drought-tolerant, requiring 4 days of treatment to reach a comparable moderate stress level, thereby ensuring that the transgenic effects in each background were evaluated under comparable stress intensities. This experimental design has been fully described in the Materials and Methods section. Finally, we emphasize two lines of evidence supporting data reliability: first, despite the differences in treatment duration and background, the direction of the transgenic effect for OE lines (relative to Nipponbare) and KO lines (relative to ZH11) is completely consistent across both backgrounds (OE lines show reduced drought tolerance, KO lines show enhanced drought tolerance), indicating that the functional orientation of OsDUF1223 is not dependent on genetic background. Second, all statistical tests were performed independently within each background. We have also added a plan in the Discussion section to construct OE and KO materials in the same genetic background (e.g., homologous complementation or gene editing knockout based on Nipponbare) in future studies to completely eliminate background confounding. We believe that the above explanations reasonably justify the experimental design and the credibility of the results.

*(See the paragraph of Section “Discussion”)*

This study demonstrates that the expression of *OsDUF1223* is significantly induced by drought stress and shows spatiotemporal specificity at the rice seedling stage. Phenotypic analysis revealed that *OsDUF1223*-knockout lines exhibited milder growth inhibition, less wilting, and higher survival rates after rewatering under drought stress relative to their Zhonghua 11 controls, whereas overexpression lines in the Nipponbare background showed lower survival rates relative to their Nipponbare controls. These results confirm that *OsDUF1223* functions as a negative regulator of drought tolerance in rice seedlings. Because the knockout and overexpression lines were generated in two different japonica backgrounds (Zhonghua 11 and Nipponbare, respectively), all comparisons were performed exclusively between each transgenic line and its corresponding wild-type control. Cross-background differences were interpreted only as qualitative trends, not as quantitative comparisons. The two wild-type cultivars exhibited inherent background-dependent differences in basal drought tolerance, with Zhonghua 11 being more tolerant than Nipponbare. Accordingly, drought treatments were applied for 3 days to the Nipponbare-based lines and for 4 days to the Zhonghua 11-based lines—durations at which each background reached a comparable moderate stress intensity—so that genotype effects could be evaluated under similar stress levels. Notably, the direction of the *OsDUF1223* effect was consistent across both backgrounds: loss of function enhanced survival, whereas overexpression reduced survival, relative to the respective control. This consistency indicates that the regulatory function of *OsDUF1223* is reproducible regardless of genetic background. Physiological and biochemical assays further indicated that knockout lines possessed stronger ROS-scavenging ability, greater osmolyte accumulation, and more stable membrane systems under drought stress, which underpin their enhanced drought resistance ^[44]^. The dynamic changes in soluble sugar and proline content reveal a key physiological pattern: although overexpression lines exhibited higher soluble sugar levels prior to treatment relative to their wild-type controls, these levels declined sharply after drought treatment—suggesting excessive consumption of osmoprotective substances or impaired homeostatic maintenance capacity ^[48]^. In contrast, knockout lines in the ZH11 background maintained relatively high levels of both soluble sugars and proline during the later stages of treatment, consistent with their enhanced drought tolerance. These results indicate that *OsDUF1223* loss of function enhances drought tolerance by reducing excessive mobilization of osmolytes under drought conditions, whereas overexpression promotes excessive mobilization of carbon and nitrogen metabolites, leading to metabolic homeostasis imbalance and drought sensitivity ^[49]^.

*(See the paragraph of Section “Materials and methods | Drought Stress Treatment”)*

Extreme drought in soil (three-leaf stage): Two-day-old seedlings were transplanted into nutrient soil (16 plants per line, 9 biological replicates) and watered with 150 mL every 2 d. Water was withheld after 10 d of growth, and drought stress was initiated when the soil water content decreased to 16% at the three-leaf stage (detected by a soil moisture meter). Because the overexpression (OE) and knockout (KO) lines were generated in two different *japonica* backgrounds, Nipponbare and Zhonghua 11, which exhibit intrinsic differences in basal drought tolerance, the stress duration for each background was determined in preliminary experiments to achieve a comparable moderate stress intensity, rather than an equal treatment duration. Specifically, stress was initiated when soil water content decreased to approximately 16% in both backgrounds. Accordingly, the Nipponbare-based lines (OE lines and their wild-type controls) were rewatered after 3 days of stress, whereas the Zhonghua 11-based lines (KO lines and their wild-type controls) were rewatered after 4 days of stress. Survival rates were scored after 9 days of recovery. All statistical comparisons were performed exclusively within the same genetic background, and cross-background observations were interpreted only as qualitative trends.

3. Distinguish correlation and causality, and optimize the elaboration of molecular regulatory mechanisms

The manuscript proposes that OsDUF1223 integrates ABA and nitrogen signaling to regulate drought tolerance, based mainly on phenotypic and transcriptomic data. However, direct molecular evidence for physical or regulatory interactions between OsDUF1223 and key ABA/nitrogen pathway components is lacking. The authors should clearly distinguish correlation versus causation in the text and discussion, and provide explicit, testable mechanistic hypotheses, which will improve the rigor of the current model without requiring large-scale molecular validation.

**Response**: Thank you for your valuable comments. We fully agree with the reminder that "correlation does not imply causation," and have further refined the wording and mechanistic discussion in the revised manuscript accordingly. First, we have retained the toned-down expressions from the previous round and added a clear "evidence stratification" statement in the Discussion section: the current conclusions are explicitly positioned as correlational evidence based on transcriptomic and phenotypic data, and any causal claims require further molecular experimental validation, thereby clearly distinguishing between "observed facts" and "mechanisms to be verified" in the text. Second, to advance the current model into testable mechanistic hypotheses, we have proposed three molecular mechanistic models in the Discussion section based on existing transcriptomic clues, each explicitly labeled as a hypothesis rather than a conclusion, along with the corresponding experimental approaches for validation: First, on the ABA signaling side, *OsDUF1223* may regulate the sensitization state of ABA signaling through direct or indirect protein–protein interactions with clade A PP2C family members (e.g., *OsPP2C49*) or SnRK2 kinases (OsSAPKs); this hypothesis can be tested by yeast two-hybrid (Y2H), bimolecular fluorescence complementation (BiFC), co-immunoprecipitation (Co-IP), and in vitro phosphorylation assays. Second, on the nitrogen metabolism side, *OsDUF1223* may influence nitrogen assimilation and root architecture through transcriptional regulation of key nitrogen signaling components, such as OsNLP family transcription factors or nitrate transporter genes; this hypothesis can be tested by DAP-seq/ChIP-seq, electrophoretic mobility shift assays (EMSA), and genetic interaction analyses with nitrogen signaling mutants. Third, at the pathway crosstalk level, we propose that *OsDUF1223* may act at the intersection of ABA and nitrogen signaling, achieving functional association through shared downstream physiological outputs (e.g., osmotic adjustment and reactive oxygen species homeostasis) rather than through direct physical interactions with pathway components; this hypothesis can be tested by constructing double mutants for genetic epistasis analysis. We believe that this "evidence stratification + testable hypotheses" approach accurately reflects the current level of support provided by the data while offering a clear experimental framework for subsequent mechanistic studies, thereby effectively enhancing the rigor of the model.

*(See the paragraph of Section “Discussion”)*

Based on the above results, we propose the following working model: *OsDUF1223* serves as a potential multifunctional regulatory hub that may coordinate the drought response in rice through two synergistic mechanisms. It should be emphasized that this model is based predominantly on correlational evidence from phenotypic and transcriptomic analyses; it does not yet establish direct physical or regulatory interactions between *OsDUF1223* and ABA or nitrogen pathway components, and causal relationships remain to be experimentally validated. First, *OsDUF1223* is functionally associated with the ABA signaling pathway, and its regulation appears to modulate both the homeostasis and the transduction of ABA signals during drought stress. At the seedling stage, in the *OsDUF1223*-overexpressing lines, drought-induced *OsDUF1223* expression is accompanied by the downregulation of bZIP23 and its downstream target *OsPP2C49*. Within this cascade, the coordinated downregulation of *OsbZIP23* and *OsPP2C49* governs two dimensions of the ABA system: the reduction of *OsPP2C49* relieves the inhibition of SnRK2 kinases, sensitizing the signaling pathway even under low ABA levels; while the downregulation of *OsbZIP23* attenuates NCED-dependent ABA biosynthesis, maintaining endogenous ABA at a low steady-state level. Together, they establish a "low-content, high-sensitivity" state. In overexpression (OE) lines, the reduced endogenous ABA is insufficient to fully activate downstream drought responses under natural drought conditions, and exogenous ABA application can rescue this defect; conversely, knockout (KO) lines exhibit elevated endogenous ABA levels and enhanced overall drought tolerance, despite reduced signaling sensitivity. Second, *OsDUF1223* is associated with the nitrogen metabolism pathway in a nitrate concentration-dependent manner, thereby remodeling root architecture. Previous studies have shown that rational nitrogen supply under drought stress can enhance plant drought tolerance ^[62]^, and efficient root architecture is fundamental for plant water uptake ^[63]^. Under specific nitrogen conditions, *OsDUF1223* reshapes root architecture by regulating key nitrogen metabolism genes. These pre-established root differences may affect overall water balance and stress adaptation when drought occurs. Notably, the disappearance of root differences under mild drought stress suggests that plants may dynamically remodel root architecture to adapt to environmental changes by coordinating the "nitrogen-water coupling" mechanism. This provides a new genetic target for understanding the regulatory mechanisms of nitrogen-water interactions, although the precise molecular mechanisms by which *OsDUF1223* perceives nitrogen signals and translates them into root developmental instructions remain to be further investigated. Based on these transcriptomic clues, we propose the following explicit, testable hypotheses. First, *OsDUF1223* may modulate ABA signaling sensitivity through direct or indirect protein–protein interactions with clade A PP2C family members (e.g., *OsPP2C49*) or SnRK2 kinases (OsSAPKs). This can be tested by yeast two-hybrid (Y2H), bimolecular fluorescence complementation (BiFC), co-immunoprecipitation (Co-IP), and in vitro phosphorylation assays. Second, *OsDUF1223* may influence nitrogen metabolism and root architecture through transcriptional regulation of key nitrogen-signaling components, such as OsNLP family transcription factors or nitrate transporter genes. This can be tested by DAP-seq/ChIP-seq, electrophoretic mobility shift assays (EMSA), and genetic interaction analyses with nitrogen-signaling mutants. Third, *OsDUF1223* may exert its effects at the ABA–nitrogen crosstalk node through shared downstream physiological outputs (e.g., osmotic adjustment and reactive oxygen species homeostasis), rather than through direct physical interactions with pathway components. This hypothesis is testable through epistasis analysis in double mutants.

This study provides evidence that *OsDUF1223* may be associated with drought tolerance in rice seedlings through its functional links to ABA signaling and nitrogen metabolism. Through this functional association with both pathways, we have preliminarily proposed a potential molecular framework underlying *OsDUF1223*-mediated drought tolerance in rice seedlings, although several limitations remain. First, transgenic materials were generated in different genetic backgrounds (Nipponbare for overexpression and ZH11 for knockout), which may interfere with direct phenotypic comparisons. Additionally, the current transcriptomic data only reflect transcriptional regulatory effects of *OsDUF1223* under the overexpression background and cannot fully exclude background-related confounding factors. Second, the direct interaction between *OsDUF1223* and core components of the ABA signaling pathway remains unclear; the crosstalk network between nitrogen and ABA signals, as well as the hormonal mechanisms regulating root plasticity, have yet to be resolved. Third, the current consistency among phenotypic, physiological, and gene expression data constitutes an indirect line of evidence supporting the negative regulation of drought tolerance by *OsDUF1223*, but has not yet reached the level of rigorous genetic causal validation—the possibility of CRISPR off-target effects in the CRISPR-generated KO lines, as well as whether *OsDUF1223* mediates its drought phenotype through known ABA signaling components such as *OsPP2C49* or *OsbZIP23*, both require further confirmation. Fourth, this study focused solely on the seedling stage; the functions of *OsDUF1223* at the adult stage and under field drought conditions, as well as its potential impacts on agronomic traits, remain to be verified.

4. Explain the logical contradiction of ABA-related experimental data

The conclusion that OsDUF1223 positively regulates ABA signaling conflicts with the reduced endogenous ABA level in OE lines (Table S2). In addition, the plant height response to ABA inhibitor treatment is stronger than that of exogenous ABA application, showing inconsistent ABA-related phenotypes. The authors should provide reasonable mechanistic explanations for these discrepancies based on current data and published evidence (e.g., differential regulation of ABA biosynthesis versus signaling) to reconcile the contradictory results.

**Response**: Thank you for your suggestions. We have revised the Discussion section accordingly and provide a unified mechanistic explanation for the apparent contradictions based on our existing data and published evidence. First, regarding the coexistence of reduced endogenous ABA content and "positive regulation of ABA signaling," we consider these two datasets not contradictory, but rather reflecting the differential regulation by *OsDUF1223* of two independent, decoupled nodes within the ABA regulatory network—what the reviewer referred to as "differential regulation of biosynthesis and signaling." On the signaling transduction side, *OsDUF1223* downregulates the negative regulator *OsPP2C49*, thereby relieving the dephosphorylation inhibition of SnRK2 kinases and enhancing ABA perception and transduction efficiency (signal sensitization). On the metabolic side, the concurrent downregulation of *OsbZIP23* within the same cascade attenuates NCED-dependent ABA biosynthesis, maintaining endogenous ABA at a low steady-state level. This combination of "low content, high sensitivity" is precisely the synergistic outcome of signal sensitization and reduced biosynthesis, allowing plants to maintain stress alertness without accumulating high concentrations of inhibitory hormones, thereby achieving a balance between growth and defense. Such regulatory modes have been documented in plant hormone networks. Second, regarding the observation that diniconazole caused more severe growth inhibition than exogenous ABA, we have verified the biochemical properties of this inhibitor and revised the relevant text accordingly: diniconazole is a triazole-type P450 inhibitor that acts by inhibiting ABA 8′-hydroxylase (CYP707A)-mediated ABA catabolism, and has been demonstrated to significantly elevate endogenous ABA levels. Accordingly, exogenously applied ABA induces CYP707A expression and is rapidly buffered by degradation, resulting in relatively moderate phenotypic changes; in contrast, diniconazole blocks this degradation pathway, allowing endogenous ABA to accumulate continuously, thereby producing stronger and more sustained signaling output in the hypersensitive OE lines, leading to more pronounced growth inhibition. We further emphasize that this "low-content, high-sensitivity" model should be interpreted in a temporal context. Under well-watered conditions, the sensitized state maintains only stress alertness without incurring a growth penalty. Upon drought onset, the limited endogenous ABA pool in the OE lines is insufficient to rapidly mount a full and robust ABA response, which accounts for their impaired drought tolerance. Exogenous ABA treatment can rescue this defect: combined treatment with 20% PEG and 50 μmol/L ABA eliminated differences in water loss rate among all lines, and scanning electron microscopy showed that the combined treatment induced stomatal closure in the OE lines, further confirming that ABA is an essential downstream signaling molecule for *OsDUF1223* in regulating drought tolerance. Based on the above analysis, we have repositioned the diniconazole experiment as a supporting validation experiment, and we believe that this mechanistic model—centered on differential regulation of biosynthesis and signal transduction—is consistent with all available data and addresses the concerns regarding data consistency.

*(See the paragraph of Section “Discussion”)*

Transcriptome analysis provided direct molecular evidence for the involvement of *OsDUF1223* in ABA signal transduction. Under drought treatment at 0 h and 1 h, differentially expressed genes in the overexpression lines were significantly enriched in the "stress response" and "hormone signal transduction" pathways, encompassing multiple core ABA signaling components. The heatmap results revealed that the abiotic stress and ABA response factor *OsPP108*, the negative regulator of ABA signaling *OsPP2C49*, and the core positive regulator of ABA signaling *OsbZIP23* were all downregulated in the overexpression lines. PP2C family proteins are core negative regulators of the ABA signaling pathway; their reduced expression relieves the inhibition of downstream SnRK2 kinases, rendering the ABA signaling pathway more prone to activation ^[52]^. Notably, *OsbZIP23* functions not only as a transcriptional activator of *OsPP2C49* but also as a positive regulator of ABA-responsive gene expression and NCED-dependent ABA biosynthesis ^[52, 53]^; its synchronous downregulation with *OsPP2C49* therefore reflects a cause-and-effect relationship within the same regulatory cascade rather than two independent signals. This coordinated reduction exerts opposing effects on ABA signaling: the loss of *OsPP2C49* sensitizes signal transduction, whereas the reduced *OsbZIP23* attenuates ABA biosynthesis and ABA-responsive gene activation. The net ABA phenotype thus depends on the balance between these two arms. Furthermore, altered expression of JAZ family proteins in the JA pathway implied a complex crosstalk between ABA and JA signals, which together shaped the *OsDUF1223*-mediated stress response network ^[54, 55]^.

This study found that endogenous ABA content in *OsDUF1223* overexpression lines was significantly lower than that in the wild type. Despite this, the OE lines exhibited significantly higher sensitivity to exogenous ABA and stronger growth inhibition in response to the triazole-type P450 inhibitor diniconazole. Diniconazole has been shown to inhibit ABA 8'-hydroxylase (CYP707A), the key enzyme in ABA catabolism, thereby elevating endogenous ABA levels ^[56]^. This differential response to the inhibitor versus exogenous ABA can be explained by their opposing effects on ABA catabolism: exogenously applied ABA is rapidly cleared by ABA-induced, CYP707A-mediated degradation, resulting in only moderate growth inhibition ^[57]^. In contrast, diniconazole blocks this degradation pathway, allowing endogenous ABA to accumulate and exert sustained signaling in the hypersensitive OE lines. This suggests that *OsDUF1223* exerts a dual regulatory effect on the ABA system. First, within the bZIP23–*OsPP2C49* cascade, although the downregulation of *OsbZIP23* attenuates its positive regulatory function in activating ABA-responsive genes, the net phenotype is dominated by the reduction of the terminal negative regulator, *OsPP2C49*. Because PP2Cs function as gatekeepers that directly dephosphorylate and inactivate SnRK2s, their removal can sensitize the signaling pathway even when ABA ligand levels are moderately reduced. Furthermore, given that *OsbZIP23* activity is primarily controlled by SnRK2-mediated phosphorylation rather than by transcript abundance, its reduced mRNA level does not necessarily translate into reduced protein activity. The elevated SnRK2 activity resulting from *OsPP2C49* reduction may even enhance the phosphorylation status of the remaining *OsbZIP23* protein, further reinforcing the hypersensitive phenotype. Second, the downregulation of *OsbZIP23* leads to attenuated NCED-dependent ABA biosynthesis, maintaining endogenous ABA at a low steady-state level. This "low-content, high-sensitivity" regulatory model allows plants to maintain a stress-alert state without accumulating high concentrations of inhibitory hormones, thereby achieving a balance between growth and defense ^[58]^. However, this model should be interpreted within a temporal context. Under well-watered conditions, the sensitized pathway primes stress alertness without a growth penalty. Conversely, upon drought onset, the limited endogenous ABA pool cannot mount a sufficiently rapid and strong ABA response, which accounts for the impaired drought tolerance of the OE lines. This interpretation is supported by exogenous ABA complementation experiments, which confirmed that ABA is an essential downstream signaling molecule for *OsDUF1223* in regulating drought tolerance. Under combined treatment with 20% PEG and 50 μmol/L ABA, the differences in water loss rate among all lines were eliminated; scanning electron microscopy observations also indicated that the combined treatment induced stomatal closure in the overexpression lines, demonstrating that exogenous ABA could rescue the drought tolerance defect caused by *OsDUF1223* overexpression. Furthermore, differentially expressed genes enriched in the "stress response" pathway included multiple ABA-dependent stress response factors: *OsNAC52* can transcriptionally activate ABA-inducible gene expression ^[59]^, and *OsANN4* can mediate ABA-induced calcium influx and regulate antioxidant enzyme activity ^[60]^. These findings further support the role of *OsDUF1223* in regulating drought tolerance through the ABA pathway.

*Added reference*:

56.	Saito S, Okamoto M, Shinoda S, Kushiro T, Koshiba T, Kamiya Y, et al. A plant growth retardant, uniconazole, is a potent inhibitor of ABA catabolism in Arabidopsis. Biosci Biotechnol Biochem. 2006;70(7):1731–9.

57.	Kushiro T, Okamoto M, Nakabayashi K, Yamagishi K, Kitamura S, Asami T, et al. The Arabidopsis cytochrome P450 CYP707A encodes ABA 8'-hydroxylases: key enzymes in ABA catabolism. Embo j. 2004;23(7):1647–56.

5. Optimize the causal logic between root architecture and drought tolerance phenotype

The proposed model that OsDUF1223 modulates drought tolerance via nitrogen-dependent root architecture suffers from logical inconsistency. KO lines with shorter roots exhibit enhanced drought tolerance, and root length differences between OE/KO lines disappear under PEG treatment while drought tolerance differences remain. The authors should revise and refine the discussion to clarify that root architecture is one of multiple regulatory pathways rather than the sole determinant of drought response, and reasonably interpret the decoupling of root phenotype and drought tolerance to strengthen logical coherence.

**Response**: Thank you for your valuable suggestions. We have revised the Discussion section accordingly to optimize the causal logic between root system architecture and drought tolerance phenotype: *OsDUF1223* primarily influences drought tolerance through the regulation of nitrogen metabolism, and changes in root architecture are one of the downstream phenotypic outputs of its nitrogen-responsive regulation, rather than the sole determinant of drought tolerance. Accordingly, we have clearly positioned root architecture as one of multiple regulatory pathways in the Discussion and revised the relevant descriptions to more accurately reflect its auxiliary role. Regarding the decoupling between root phenotype and drought tolerance, we propose that under normal conditions, the longer roots of the OE lines are a pleiotropic phenotype resulting from *OsDUF1223*-mediated regulation of nitrogen metabolism-related genes (e.g., *OsRNR10*). Under PEG-simulated drought stress, plants initiate root system plasticity responses. In the Nipponbare background, root growth inhibition was more pronounced in the OE lines, while in the ZH11 background, inhibition was milder in the KO lines, leading to the disappearance of root length differences. However, the difference in drought tolerance was retained, precisely indicating that absolute root length is not a key determinant of drought tolerance. The persistent difference in drought tolerance is primarily mediated by nitrogen metabolism regulation that remains active during drought stress (transcriptomic analysis showed continuous enrichment of nitrogen metabolism pathways at 8 h, 16 h, and 24 h). This logic has been integrated into the revised Discussion section to enhance the logical coherence of the causal relationship between root architecture and drought tolerance.

*(See the paragraph of Section “Discussion”)*

Our study demonstrates that *OsDUF1223* positively regulates root elongation in rice seedlings. Under normal conditions, overexpression (OE) lines in the Nipponbare background exhibited significantly longer roots, while knockout (KO) lines in the ZH11 background displayed markedly shorter roots. However, under mild drought stress, these differences diminished: OE lines showed greater root growth inhibition, whereas KO lines exhibited milder suppression, indicating that root plasticity is dynamically modulated by water status. The disappearance of root length differences under drought, while drought tolerance differences persisted, suggests that root architecture is not a direct causal pathway through which *OsDUF1223* regulates drought tolerance. Transcriptomic analyses provided key insights into the core mechanism. KEGG enrichment analysis revealed that, even at the 0 h time point, upregulated differentially expressed genes in the overexpression lines were significantly enriched in the "nitrogen metabolism" pathway, suggesting that *OsDUF1223* exerts a constitutive regulatory effect on nitrogen metabolism. Although the 0 h enrichment may partially reflect genotypic differences in homeostatic state—including potential effects of transgene insertion—the consistent and significant enrichment of this pathway at 8 h, 16 h, and 24 h under stress indicates that this regulation is both maintained and specifically engaged during drought stress. Therefore, we propose that the drought-responsive specificity of the nitrogen metabolism pathway is primarily supported by its dynamic transcriptional changes during the later stages of stress, rather than being invalidated by the 0 h enrichment alone. Taken together, *OsDUF1223* participates in the drought response by regulating nitrogen metabolism, and its function is not limited to root development.

Among the overlapping DEGs consistently upregulated across all time points, *OsRNR10* is known to inhibit NO₃⁻ uptake and root development by modulating auxin accumulation ^[59]^. Theoretically, *OsRNR10* upregulation should suppress root growth; however, OE lines exhibited elongated roots. This contradiction has not been mechanistically resolved in the present study; it may reflect functional plasticity of *OsRNR10* across different genetic backgrounds or stress conditions, or may suggest that *OsDUF1223* functionally antagonizes *OsRNR10* through yet-to-be-identified downstream factors. Resolving this contradiction will require further auxin phenotypic analyses and genetic interaction experiments between *OsRNR10* and *OsDUF1223*. Nitrate concentration gradient experiments demonstrated that the root phenotype effects of *OsDUF1223* are dependent on nitrogen availability: under nitrogen-free or low-nitrogen conditions, OE lines exhibited significantly shorter roots than wild-type plants, whereas no difference was observed under high-nitrogen conditions. Conversely, KO lines in the ZH11 background showed the opposite trend, suggesting that *OsDUF1223*-mediated root developmental regulation is closely linked to nitrogen signaling pathways. These findings reveal an association between nitrogen signaling and root development, and provide a reasonable explanation for the apparent contradiction between *OsRNR10* upregulation and root elongation in overexpression lines: the final root phenotype is not determined by a single gene, but rather reflects the integrated output of the nitrogen signaling network. Under nitrogen-sufficient conditions, the net balance between the auxin-mediated inhibitory effect of *OsRNR10* and the promoting effect of *OsDUF1223* on root growth may favor elongation. When nitrogen availability decreases, this balance shifts toward inhibition, consistent with the shorter roots observed in overexpression lines under nitrogen-free or low-nitrogen conditions. Definitive resolution of this balance will require auxin phenotypic analyses and genetic interaction experiments between *OsRNR10* and *OsDUF1223*.

Reviewer #3:

The authors have addressed the comments and suggestions made by the reviewers and modified the manuscript accordingly.

However, I strongly oppose removing experimental design information from the Figure legends as indicated by the authors:

"In addition, we will delete the sample size information (e.g., "n=3") from the figurelegends and uniformly incorporate this information into the Materials and Methods section to ensureconsistent and standardized presentation throughout the manuscript."

**Response**: We sincerely thank the reviewer for the correction and accept this suggestion. We acknowledge that our previous decision to remove sample size information (e.g., "n=3") from the figure legends was indeed inappropriate. We have now revised the manuscript to include the sample size and replication information in the corresponding figure legends, while retaining the complete description in the Materials and Methods section, to ensure consistency and readability of the information presented. We once again thank the reviewer for the valuable comments.

*(See the paragraph of Section “Results | Fig 1. Expression pattern analysis of the OsDUF1223 gene and subcellular localization of its protein”)*

Note: A–B. Relative transcript levels of the *OsDUF1223* gene after 24 h of treatment with 20% PEG and 50 μmol/L ABA, respectively (n = 3, technical replicates); C. GUS staining results of the *OsDUF1223* gene after 0.5 h of treatment with 20% PEG and 50 μmol/L ABA, respectively. Scale bar = 2 cm; D. Relative transcript levels of the *OsDUF1223* gene in different spatiotemporal tissue regions (n = 3, technical replicates); E. GUS staining results of the *OsDUF1223* gene in different spatiotemporal tissue regions. Scale bar = 2 cm; F. Subcellular localization of the *OsDUF1223*-GFP fusion protein. Scale bar = 100 μm. Data are presented as mean ± SEM and were analyzed by one-way ANOVA with Tukey's multiple comparison test (A–B). * indicates a significant difference (0.01 < *P* < 0.05), ** indicates an extremely significant difference (*P* < 0.01). GUS staining (C, E) and subcellular localization (F) were performed with three independent biological replicates, all yielding consistent results; representative images are shown.

*(See the paragraph of Section “Results | Fig 2. Drought stress tolerance of OsDUF1223 transgenic seedlings”)*

Note: A. Growth phenotypes of Nipponbare WT and OE lines under normal conditions and 10% PEG treatment (representative images shown). Scale bar = 4 cm; B. Growth phenotypes of Zhonghua 11 WT and KO lines under normal conditions and 10% PEG treatment (representative images shown). Scale bar = 4 cm; C–D. Statistical analysis of plant height and root length of Nipponbare WT and OE lines under normal conditions and 10% PEG treatment (n = 16 plants); E–F. Statistical analysis of plant height and root length of Zhonghua 11 WT and KO lines under normal conditions and 10% PEG treatment (n = 16 plants); G–H. Phenotypic analysis and survival rate of Nipponbare WT and OE seedlings at the three-leaf stage before and after drought stress treatment (n = 9 biological replicates, 16 plants per replicate); I–J. Phenotypic analysis and survival rate of Zhonghua 11 and KO seedlings at the three-leaf stage before and after drought stress treatment (n = 9 biological replicates, 16 plants per replicate). Data are presented as mean ± SEM and were analyzed by one-way ANOVA with Tukey's multiple comparison test (H, J) and two-way ANOVA with Tukey's multiple comparison test (C–F), ** denotes an extremely significant difference (*P* < 0.01).

*(See the paragraph of Section “Results | Fig 3. Physiological and Biochemical Index Analysis of OsDUF1223 Transgenic Seedlings”)*

Note: A. NBT staining of transgenic lines (n = 3 biological replicates; representative images shown). Scale bar = 1 cm; B. DAB staining of transgenic lines (n = 3 biological replicates; representative images shown). Scale bar = 1 cm; C–D. SOD activity in Nipponbare WT/OE and Zhonghua 11 WT/KO lines (n = 3 technical replicates); E–F. Proline content in Nipponbare WT/OE and Zhonghua 11 WT/KO lines (n = 3 technical replicates); G–H. Soluble sugar content in Nipponbare WT/OE and Zhonghua 11 WT/KO lines (n = 3 technical replicates); I–J. MDA content in Nipponbare WT/OE and Zhonghua 11 WT/KO lines (n = 3 technical replicates). Data are presented as mean ± SEM and were analyzed by two-way ANOVA with Tukey’s multiple comparison test (C–J).. ** indicates a highly significant difference (*P* < 0.01).

*(See the paragraph of Section “Results | Fig 4. Water Loss Rate and Stomatal Morphology of OsDUF1223 Transgenic Seedlings Under Drought Stress”)*

Note: Water loss rate of transgenic lines under drought stress: A. Nipponbare WT and OE lines (n = 9 plants); B. Zhonghua 11 WT and KO lines (n = 9 plants); Water loss rate of detached leaves of transgenic lines: C. Nipponbare WT and OE lines (n = 9 plants); D. Zhonghua 11 WT and KO lines (n = 9 plants); E. Leaf stomatal morphological characteristics of WT and transgenic seedlings after 8 h of drought stress (5 fields of view were selected from the same plant, representative images shown), red * indicates stomatal position. Scale bar = 5 μm; F, I: Stomatal aperture size statistics of OE plants in the Nipponbare background and KO plants in the Zhonghua 11 background (n = 20 stomata); G, J: Stomatal density statistics of OE plants in the Nipponbare background and KO plants in the Zhonghua 11 background (n = 5 fields of view); H, K: Stomatal pore index (SPI) statistics of OE plants in the Nipponbare background and KO plants in the Zhonghua 11 background (n = 20 stomata). Data are presented as mean ± SEM and were analyzed by unpaired, two-tailed Student’s t-test (F-K), one-way ANOVA with Tukey’s multiple comparison test (A-B) and two-way ANOVA with Tukey’s multiple comparison test (C-D), ** indicates an extremely significant difference (*P* < 0.01).

*(See the paragraph of Section “Results | Fig 5. Transcriptome Analysis and Validation of OsDUF1223 OE Plants”)*

Note: A. Number of DEGs under different stress treatment durations; B. Overlapping downregulated DEGs commonly expressed across time points; C. Overlapping upregulated DEGs commonly expressed across time points; D–G. GO enrichment analysis bar charts under different stress treatment durations, with red boxes marking "abscission", "signal transduction", "response to abiotic stimulus", and "response to stress"; Relative transcript levels of genes *OsLOX4*, *OsNR2*, *OsbZIP23*, *OsPAL9*, and *LOC_Os1g40860* before and after drought stress for 1 h in each line: H. Nipponbare WT and OE lines (n = 3, technical replicates); I. Zhonghua 11 WT and KO lines (n = 3, technical replicates). Data are presented as mean ± SEM and were analyzed by two-way ANOVA with Tukey’s multiple comparison test (H-I). * denotes a significant difference (0.01 < *P* < 0.05), ** denotes an extremely significant difference (*P* < 0.01).

*(See the paragraph of Section “Results | Fig 6. Expression and Validation of Hormone Signaling Pathway-Related Genes in OsDUF1223 Transgenic Lines”)*

Note: A. Heatmap of DEGs significantly enriched in signaling pathways (GO) and hormone signaling pathways (KEGG); B. Relative transcript levels of the *OsDUF1223* gene after 24 h of treatment with 100 μmol/L MeJA (n = 3, technical replicates). Relative transcript levels of genes *OsNCED3*, *OsANN4*, *OsNAC52*, *OsPP2C49*, *OsPP108* and *OsJAZ5* in each line before and after drought stress treatment for 1 h: C. Nipponbare WT and OE lines (n = 3, technical replicates); D. Zhonghua 11 WT and KO lines (n = 3, technical replicates). Data are presented as mean ± SEM and were analyzed by one-way ANOVA with Tukey’s multiple comparison test (B) and two-way ANOVA with Tukey’s multiple comparison test (C-D). * denotes a significant difference (0.01 < *P* < 0.05), ** denotes an extremely significant difference (*P* < 0.01).

*(See the paragraph of Section “Results | Fig 7. Growth Performance of OsDUF1223 Transgenic Seedlings Under ABA Treatment Conditions”)*

Note: Plant height phenotypes and statistical analysis of transgenic lines under normal conditions and 15 μmol/L ABA treatment: A–B. Nipponbare WT and OE lines (B: n = 16 plants); C–D. Zhonghua 11 WT and KO lines (D: n = 16 plants). Plant height phenotypes and statistical analysis of transgenic lines under normal conditions, 10 μmol/L diniconazole, and 20 μmol/L diniconazole treatment: E–F. Nipponbare WT and OE lines (F: n = 16 plants); G–H. Zhonghua 11 WT and KO lines (H: n = 16 plants). Data are presented as mean ± SEM and were analyzed by two-way ANOVA with Tukey’s multiple comparison test (B, D, F, H). * denotes a significant difference (0.01 < P < 0.05), ** denotes an extremely significant difference (P < 0.01).

*(See the paragraph of Section “Results | Fig 8. Nitrogen Response of OsDUF1223 Transgenic Plants”)*

Note: Relative transcript levels of genes *LOC_Os05g48200* and *OsRNR10* in transgenic lines before and after drought stress treatment for 1 h: A. Nipponbare WT and OE lines (n = 3, technical replicates); B. Zhonghua 11 WT and KO lines (n = 3, technical replicates); C–D. Root system architecture phenotypes and root length statistics of Nipponbare WT and OE lines (D: n = 16 plants). Scale bar = 2 cm; E–F. Root system architecture phenotypes and root length statistics of Zhonghua 11 WT and KO lines (F: n = 16 plants). Scale bar = 2 cm; G–H. Root growth phenotypes of transgenic lines under different NO₃⁻ concentrations. Scale bar = 2 cm; I–J. Statistical analysis of root length of transgenic lines under different NO₃⁻ concentrations (n = 24 plants). Data are presented as mean ± SEM and were analyzed by one-way ANOVA with Tukey’s multiple comparison test (D, F) and two-way ANOVA with Tukey’s multiple comparison test (A-B, I-J). * denotes a significant difference (0.01 < *P* < 0.05), ** denotes an extremely significant difference (*P* < 0.01).
