# Supplementary material for: OsDUF1223 negatively regulates seedling drought tolerance and modulates nitrate-dependent root growth in rice
Source: PLoS Genet. 2026 Sep 3;22(9):e1012295. doi: 10.1371/journal.pgen.1012295 (PMC13541127; doi:10.1371/journal.pgen.1012295)
Supplement: Peer review history

# Peer review: Decision Letter 0

**DOI:** 10.1371/journal.pgen.1012295.r001

**Authors:** Tomokazu Kawashima, Quan Wang

3 Jul 2026

PGENETICS-D-26-00390

OsDUF1223 Integrates ABA and Nitrogen Signals to Negatively Regulate Drought Tolerance in Rice Seedlings

PLOS Genetics

Dear Dr. li,

Thank you for submitting your manuscript to PLOS Genetics. After careful consideration, we feel that it has merit but does not fully meet PLOS Genetics's publication criteria as it currently stands. Therefore, we invite you to submit a revised version of the manuscript that addresses the points raised during the review process.

Please submit your revised manuscript by Sep 01 2026 11:59PM. If you will need more time than this to complete your revisions, please reply to this message or contact the journal office at plosgenetics@plos.org. When you're ready to submit your revision, log on to https://www.editorialmanager.com/pgenetics/ and select the 'Submissions Needing Revision' folder to locate your manuscript file.

Please include the following items when submitting your revised manuscript:

* A letter that responds to each point raised by the editor and reviewer(s). You should upload this letter as a separate file labeled 'Response to Reviewers'. This file does not need to include responses to any formatting updates and technical items listed in the 'Journal Requirements' section below.

* A marked-up copy of your manuscript that highlights changes made to the original version. You should upload this as a separate file labeled 'Revised Manuscript with Track Changes'.

* An unmarked version of your revised paper without tracked changes. You should upload this as a separate file labeled 'Manuscript'.

If you would like to make changes to your financial disclosure, competing interests statement, or data availability statement, please make these updates within the submission form at the time of resubmission. Guidelines for resubmitting your figure files are available below the reviewer comments at the end of this letter.

As the corresponding author, your ORCID iD is verified in the submission system and will appear in the published article. PLOS supports the use of ORCID, and we encourage all coauthors to register for an ORCID iD and use it as well. Please encourage your coauthors to verify their ORCID iD within the submission system before final acceptance, as unverified ORCID iDs will not appear in the published article. Only the individual author can complete the verification step; PLOS staff cannot verify ORCID iDs on behalf of authors.

We look forward to receiving your revised manuscript.

Kind regards,

Tomo Kawashima

Academic Editor

PLOS Genetics

Quan Wang

Section Editor

PLOS Genetics

Aimée Dudley

Editor-in-Chief

PLOS Genetics

Anne Goriely

Editor-in-Chief

PLOS Genetics

**Additional Editor Comments (if provided):**

**Journal Requirements:**

If the reviewer comments include a recommendation to cite specific previously published works, please review and evaluate these publications to determine whether they are relevant and should be cited. There is no requirement to cite these works unless the editor has indicated otherwise.

1) Please ensure that the CRediT author contributions listed for every co-author are completed accurately and in full.

At this stage, the following Authors/Authors require contributions: Xiulin Zhao, Ziyi Wang, Mingfei Chen, Lingxiang Lu, Hui Lin, Xiaofei Zan, Xiaomei Jia, Xiaoying Ye, Rongjun Chen, Jianqing Zhu, Jun Zhu, and lihua li. Please ensure that the full contributions of each author are acknowledged in the "Add/Edit/Remove Authors" section of our submission form.

The list of CRediT author contributions may be found here: https://journals.plos.org/plosgenetics/s/authorship#loc-author-contributions

2) Please provide an Author Summary. This should appear in your manuscript between the Abstract (if applicable) and the Introduction, and should be 150-200 words long. The aim should be to make your findings accessible to a wide audience that includes both scientists and non-scientists. Sample summaries can be found on our website under Submission Guidelines:

https://journals.plos.org/plosgenetics/s/submission-guidelines#loc-parts-of-a-submission

3) Please upload all main figures as separate Figure files in .tif or .eps format. For more information about how to convert and format your figure files please see our guidelines:

https://journals.plos.org/plosgenetics/s/figures

4) Please upload a copy of Figures FIG 1 A-F, 2A-J, 3A-J, 4A-I, 5A-I.6A-D, 7A-H, and 8A-J which you refer to in your text on pages 7, 10, 12, 15, 17, 20, 23, and 25. Or, if the figure is no longer to be included as part of the submission please remove all reference to it within the text.

5) We notice that your supplementary Figures, and Tables are included in the manuscript file. Please remove them and upload them with the file type 'Supporting Information'. Please ensure that each Supporting Information file has a legend listed in the manuscript after the references list.

6) Please amend your detailed Financial Disclosure statement. This is published with the article. It must therefore be completed in full sentences and contain the exact wording you wish to be published.

i) State the initials, alongside each funding source, of each author to receive each grant. For example: "This work was supported by the National Institutes of Health (####### to AM; ###### to CJ) and the National Science Foundation (###### to AM)."

ii) State what role the funders took in the study. If the funders had no role in your study, please state: "The funders had no role in study design, data collection and analysis, decision to publish, or preparation of the manuscript."

iii) If any authors received a salary from any of your funders, please state which authors and which funders..

If you did not receive any funding for this study, please simply state: u201cThe authors received no specific funding for this work.u201d.

7)We have amended your Competing Interest statement to comply with journal style. We kindly ask that you double check the statement and let us know if anything is incorrect.

**Reviewers' comments:**

Reviewer's Responses to Questions

**Comments to the Authors:**

**Please note here if the review is uploaded as an attachment.**

Reviewer #1: In this manuscript, Zhao et al. identify OsDUF1223 as a negative regulator of drought tolerance in rice. Based on transcriptomic and physiological analyses, the authors propose a model in which OsDUF1223 modulates drought responses through ABA signaling and nitrogen response pathways. The identification of a previously uncharacterized factor involved in drought tolerance is interesting and may provide new insights into stress adaptation mechanisms in rice.

However, several aspects of the study would benefit from further experimental support. In particular, the relatively small sample sizes used throughout the study make it difficult to fully assess the robustness and reproducibility of the presented data. Additional experimental validation and expanded datasets would strengthen the mechanistic conclusions of the study.

Major Comments

1. The number of biological replicates used throughout the study is insufficient to support robust statistical conclusions. Most experiments were performed with only n = 3 biological replicates, which substantially limits the reliability and reproducibility of the presented data. The authors should increase the number of biological replicates (n=10-15) and provide additional experimental validation. In its current form, the statistical power of the analyses appears inadequate.

2. Regarding lines 123–124, the authors state that OsDUF1223 was identified from publicly available microarray datasets; however, the supporting data are not shown in the manuscript. The authors should include the corresponding transcriptomic analysis demonstrating how OsDUF1223 was selected. In particular, expression profiling such as a heatmap should be provided to demonstrate that OsDUF1223 is specifically induced under drought stress conditions.

3. The biological and evolutionary context of OsDUF1223 remains unclear. The authors should clarify whether OsDUF1223 belongs to a gene family in rice and whether homologs are conserved in other plant species. Such information would help readers evaluate the broader significance and potential universality of the findings.

4. In Fig. 2D, the OsDUF1223 overexpression lines exhibit increased root length. However, this phenotype is not observed in Fig. 8I under the nitrate 2 mM and 10 mM conditions. The authors should clearly indicate the nitrogen concentrations used in each experiment and discuss the possible reasons underlying these apparently inconsistent phenotypic results.

Minor Comments

1. The discussion in lines 94–98 regarding nitrate ion uptake appears unnecessary, as this study does not experimentally address nitrate uptake mechanisms. This section of the Introduction should therefore be shortened or removed.

2. The plasma membrane marker used in Fig. 1F should be clearly described in the Materials and Methods section.

3. The abbreviation “SOD activity” in line 226 should be defined at its first appearance in the main text.

Reviewer #2: This work reports the identification of a novel rice gene OsDUF1223 with potential functions in regulating drought tolerance, and the research strategy adopted shows distinct innovative value. While the study delivers promising preliminary insights, there remain several key aspects related to experimental design, data explanation and logical coherence that could be further optimized to strengthen the robustness of the conclusions. The manuscript will be suitable for publication provided all the concerns outlined below are fully addressed. Specific points for improvement are listed as follows:

1. The title of Figure 1, “Expression pattern analysis and subcellular localization of the OsDUF1223 gene,” is imprecise and should refer to the subcellular localization of the OsDUF1223 protein. In addition, the RFP marker used in Figure 1F is described as a plasma membrane marker (lines 162–164), but neither the main text nor the Materials and Methods section identifies this marker. Furthermore, the authors state in line 165 that the RFP and GFP signals “perfectly overlapped,” yet the merge in Figure 1F does not show complete colocalization. Quantitative colocalization analysis or clearer magnified images are recommended.

2. In lines 168–192, overexpression (OE) lines were generated in the Nipponbare background, while knockout (KO) lines were generated in the Zhonghua 11 background. Although both are japonica varieties, the genetic backgrounds are not identical. Consequently, all transgenic lines can only be compared with their respective wild type controls, and direct phenotypic comparisons between OE and KO lines are not valid. Moreover, in Figures 2G–J, the survival rates of ZH11 and Nipponbare under the same treatment differ substantially, making it difficult to determine whether the observed phenotypes are directly linked to OsDUF1223 expression levels. Please also explain why the drought treatment duration for OE lines (3 days) and KO lines (4 days) was not consistent in the experiments shown in Figures 2G–J.

3. In the abstract (lines 36–38), in the ABA signaling experiments (lines 379–397), and in the nitrogen response and root architecture experiments (lines 408–435), the authors repeatedly propose that OsDUF1223 “integrates ABA and nitrogen signals” to regulate drought tolerance. However, no direct molecular evidence (e.g., protein protein interactions, phosphorylation regulation) is provided to demonstrate that OsDUF1223 physically interacts with key components of ABA signaling or nitrogen metabolism pathways. The current conclusions are based primarily on transcriptomic and phenotypic data. The authors should clearly distinguish between correlation and causation and propose testable hypotheses for the molecular mechanism.

4. In lines 389–390, the authors state that “OsDUF1223 positively regulates the ABA signaling pathway.” Yet, Table S2 shows that endogenous ABA content is reduced in OsDUF1223 overexpression lines. If OsDUF1223 indeed positively regulates ABA signaling, why is endogenous ABA decreased? Moreover, if the overexpression lines are merely hypersensitive to ABA, why does treatment with an ABA synthesis inhibitor cause a more dramatic change in plant height (from ~17 cm to ~7 cm) than exogenous ABA application (from ~20 cm to ~11 cm)? The authors should provide a plausible explanation for this discrepancy.

5. In lines 408–409 and elsewhere in the manuscript, the authors propose that “OsDUF1223 affects drought tolerance by regulating nitrogen mediated root architecture.” However, OE lines have longer roots than the wild type, while KO lines have shorter roots. Why would a shorter rooted line be more drought tolerant? Furthermore, under 10% PEG simulated drought conditions, the difference in root length between OE and KO lines disappears, whereas their drought tolerance phenotypes remain distinct. This indicates that the observed differences in root architecture lack a logical causal relationship with drought tolerance.

Reviewer #3: In this manuscript, Xiulin Zhao, Ziyi Wang and colleagues investigate the function of OsDUF1223 (LOC_Os07g37310), a chromosome-7 gene encoding a 274-aa protein of unknown function, in the drought response of rice seedlings. Using overexpression (OE) lines in the Nipponbare background and CRISPR knockout (KO) lines in the Zhonghua 11 background, the authors report that OsDUF1223 acts as a negative regulator of seedling drought tolerance, and they propose that it does so by simultaneously tuning ABA signaling/homeostasis and nitrate-dependent root architecture.

The main findings of the paper are:

OsDUF1223 is induced by PEG (drought) and exogenous ABA, is expressed mainly in seedling shoots, and localizes to the plasma membrane in transient N. benthamiana assays (Fig 1).

Under drought, OE lines show lower survival, higher water loss, stronger ROS accumulation, lower SOD activity, lower proline/soluble sugar and higher MDA, while KO lines show the opposite within their own background (Figs 2–3).

OE leaves have larger stomatal apertures and lower stomatal density (Fig 4).

A five–time-point drought RNA-seq of OE plants nominates differentially expressed genes in hormone signaling and nitrogen metabolism, including OsPP2C49, OsbZIP23, OsNCED3 and OsRNR10 (Figs 5–6, Figs S2–S3).

OE plants are hypersensitive to exogenous ABA and to the synthesis inhibitor diniconazole, have lower endogenous ABA, and exogenous ABA abolishes the water-loss difference between OE and WT (Fig 7, Fig S4, Tables S1–S2).

Root elongation is positively regulated by OsDUF1223 in a nitrate-concentration-dependent manner (Fig 8).

This is a broad and clearly effortful study, and I appreciate the range of phenotyping the authors have assembled around a gene that genuinely deserves attention. DUF proteins are understudied, and a node that couples nitrogen status to ABA-mediated drought responses would be of interest to many readers. To my mind the nitrate-concentration dependence of the root phenotype (Fig 8G–J) is the most novel single observation in the paper, and within each genetic background the survival and physiology data are internally coherent.

Several of my comments below can be addressed without new experiments. That said, I have three substantive concerns that bear directly on the central message and that I think need to be resolved before the conclusions are defensible: (i) the OE and KO lines are in different genetic backgrounds and the paper's mirror-image "OE up / KO down" logic is never tested against a shared control; (ii) the endogenous-hormone data that anchor the ABA model are unreplicated and internally contradictory; and (iii) the "integration" of ABA and nitrogen signaling is asserted rather than demonstrated. I have numbered my comments to make a point-by-point response easier, and I hope they are useful to the authors and the editor.

The OE and KO lines are in different genetic backgrounds, and the wild-type values differ enough between them that I am not sure the mirror-image comparison is sound. OE lines are Nipponbare and KO lines are Zhonghua 11 (lines 158–160, 598–602). Almost every conclusion is phrased as "OE does X, KO does the opposite," but the two halves are never measured against the same control — each transgenic set can only be compared to its own WT. The data make me worried this matters in practice. At the same 2 mmol/L NO₃⁻, the Nipponbare WT root length is 5.27 cm while the ZH11 WT is 7.16 cm (lines 420–422). Baseline ABA differs about two-fold between the two WTs (8.05 vs 16.45 ng/g; Tables S1–S2), and baseline JA differs similarly. As in any cross-background comparison, the variation between the two wild types can be as large as the differences the authors attribute to the gene, which makes the "opposite phenotype" framing fragile. I appreciate that the authors flag this in the limitations (lines 572–577), but as written it is a footnote to conclusions that depend on it. The clean solution is same-background loss- and gain-of-function lines, which the authors say are forthcoming (lines 602–604). If those are not feasible on a reasonable timescale, I would ask the authors to recast the Results and Discussion as two independent, within-background experiments, remove the mirror-image phrasing, and restrict each mechanistic claim to the background in which it was observed.

The endogenous-hormone data are internally contradictory and are reported without replication or statistics. This is the comment I would most like to see addressed, because the "low-content, high-sensitivity" ABA model rests on it.

Direction. In the Nipponbare WT, ABA decreases under drought (Table S2: 8.05 → 5.63 ng/g), whereas in the ZH11 WT it increases (Table S1: 16.45 → 23.2 ng/g). Drought-induced ABA accumulation is one of the most reproducible observations in plant biology, so a wild type that loses ABA under drought is a red flag, and the two wild types cannot both be correct. The JA values behave the same way (WT JA falls under drought in Nipponbare, 3.23 → 1.36, but rises in ZH11, 8.33 → 9.86). Could the authors explain this, and confirm the drought treatment imposed comparable water stress in both experiments?

Replication and statistics. The Methods state only "two technical replicates" with no biological replication for the hormone measurements (lines 700–701), and Tables S1–S2 report single point values with no error and no test. Yet the text repeatedly calls the differences "significant" (e.g., lines 360–364). Please provide at least three biological replicates, report the variance, and test the differences formally. I note this also conflicts with the statement at lines 726–727 that all data have at least three biological replicates.

Inconsistent panels. Table S1 reports ABA/JA/SA/GA3/IAA but Table S2 reports only ABA/JA/SA. Please measure the same panel in both backgrounds.

The transcriptome that anchors the mechanism was generated only in the OE/Nipponbare background. The RNA-seq is OE vs Nipponbare WT only (lines 288–289, 713–714), and the "opposite" molecular response in KO is then asserted from a handful of RT-qPCR genes (Figs 5I, 6D, 8B) rather than shown transcriptome-wide. Because overexpression of a membrane protein is prone to gain-of-function and insertion-site effects, the direction of the key claims (PP2C49, bZIP23, NCED3, nitrogen-metabolism enrichment) would be much more secure with a matched KO/ZH11 RNA-seq at the same time points. Relatedly, the 0 h OE-vs-WT contrast (132 up / 196 down; lines 291–293) is a steady-state genotype difference that is partly attributable to the construct itself, so I would be cautious about interpreting the 0 h "nitrogen metabolism" and "fatty acid elongation" enrichment (Fig S2) as drought mechanism without the KO comparison.

The proposed ABA-signaling logic is internally inconsistent at OsbZIP23. The model holds that OE down-regulates the negative regulator OsPP2C49 to derepress ABA signaling and so become ABA-hypersensitive (lines 471–477, 482–490). But the authors also report OsbZIP23 as down-regulated in OE (validated in Fig 5H–I), and OsbZIP23 is a positive regulator of ABA sensitivity (Xiang et al. 2008, ref 47) that, through feedback, promotes both ABA-responsive gene expression and NCED-dependent biosynthesis (Zong et al. 2016, ref 48). Lower OsbZIP23 would therefore predict reduced ABA sensitivity and lower ABA content. The lower content is consistent with the data, but the reduced sensitivity is the opposite of the hypersensitive phenotype the authors observe. As written, the model selects OsPP2C49 while ignoring that its own upstream regulator moves the wrong way. I would ask the authors to reconcile this, or to acknowledge that the transcriptional changes do not coherently predict the hypersensitivity (which may instead be post-transcriptional).

I am not sure the diniconazole experiment supports "positive regulation of ABA signaling." OE plants are shorter than WT under exogenous ABA (Fig 7A–B) and also shorter than WT under the ABA-synthesis inhibitor diniconazole "at all concentrations" (lines 384–388, Fig 7E–F). If "at all concentrations" includes the untreated control, then OE is simply shorter at baseline and the diniconazole effect is not ABA-specific. Adding ABA and removing ABA should not move OE height in the same direction relative to WT if the interpretation is a straightforward shift in ABA sensitivity. Could the authors show the control side by side, test the genotype × treatment interaction, and clarify whether the data distinguish "ABA-hypersensitive" from "constitutively shorter"?

The stomatal explanation is internally muddled, and there is no direct conductance measurement. OE is reported to have both larger aperture and lower density (Fig 4E–I; abstract lines 31–33). These act in opposite directions on transpiration — lower density should reduce water loss — so attributing the higher OE water loss to "stomatal morphology" is not straightforward. A net measure would resolve this: a stomatal pore-area index, or, better, direct gas exchange (porometer/IRGA) giving stomatal conductance, rather than SEM morphology alone. Two smaller points: the SEM is a single critical-point-dried snapshot at 8 h and does not capture closure dynamics, and the legend reports n = 3 while the Methods describe "5 visual fields" per line (lines 686–687), which reads as pseudoreplication. Please clarify the experimental unit and the replication.

The "integration" of ABA and nitrogen signaling is asserted but not demonstrated; I would temper the title and the model accordingly. The data show that OE perturbs ABA-associated readouts and, separately, nitrate-dependent root growth. Nothing in the manuscript shows that the two inputs converge at OsDUF1223 — there are no protein interactions, no biochemistry, and no shared downstream target, and the protein is of unknown function. "Integrates ABA and nitrogen signals" (title; abstract lines 35–38; model lines 551–569) therefore reads as stronger than the result. I would suggest a title closer to "OsDUF1223 negatively regulates seedling drought tolerance and modulates nitrate-dependent root growth in rice," and presenting the two-arm figure explicitly as a hypothesis. If the authors wish to keep "integration," it needs an experiment showing convergence.

The nitrogen → root → drought causal chain is weak, and the two nitrate experiments appear to disagree at 2 mmol/L NO₃⁻. The genotype differences in root length disappear under the mild drought (10% PEG) actually used (lines 178, 508–509), which makes it hard to argue that "pre-formed root differences" drive drought outcomes when those differences vanish under the relevant stress. Separately, the two nitrate experiments seem inconsistent: in nitrogen-free Hoagland's with 2 mmol/L NO₃⁻ for 12 d, OE roots are much longer than WT (lines 417–423), whereas in the gradient with a 1 mmol/L glutamine background for 8 d there is no difference at 2 mmol/L NO₃⁻ (lines 430–431). The authors attribute this to the organic-nitrogen background, which is plausible, but the two experiments also differ in duration (12 vs 8 d), so the glutamine effect is confounded with time. A single experiment that varies only the glutamine background at fixed duration and NO₃⁻ would isolate the effect being claimed.

The OsRNR10 result contradicts the cited literature and is resolved only by assertion. The authors note that OsRNR10 (Huang et al. 2023, Nature Plants; ref 56) inhibits NO₃⁻ uptake and root development through auxin, yet here OsRNR10 is up-regulated in OE while roots are longer (lines 531–535). Describing this as a "complex regulatory network or compensatory mechanism" (lines 534–536) restates the contradiction rather than resolving it. As written this is a weakness in the nitrogen arm rather than support for it. Could the authors provide evidence for the proposed compensation (for example auxin measurements, or an OsRNR10-dependence test), or present it explicitly as an unresolved discrepancy?

No complementation, allelism, or genetic-interaction test. To place OsDUF1223 in the ABA and nitrate pathways it is associated with, it would help to go beyond OE vs KO. A complementation/rescue of the KO (re-introducing OsDUF1223 into the ZH11 background and recovering the OE-like phenotype) would also control for CRISPR off-target effects. More informative still would be a genetic-interaction test — for example, whether the OE drought phenotype requires ABA signaling (in an ospp2c49 or osbzip23 background) and whether the root phenotype requires OsRNR10. I recognize these are non-trivial to generate; I raise them because the causal architecture in the model (lines 551–569) is currently inferred entirely from expression correlations and pharmacology.

CRISPR knockout controls. Only the 1-bp frameshift deletion is described (lines 160–162). Please report whether the KO lines are Cas9-free (transgene-segregated), an off-target assessment, and confirmation that the frameshift produces a true null (loss of transcript or protein), since the entire loss-of-function argument rests on it.

Does the OE dosage track the phenotype? OE-4 appears to be the strongest expresser (Fig S1A), but the three-leaf survival rates (OE-1 29.9%, OE-4 33.4%, OE-5 41.7%; lines 184–185) do not obviously follow expression level. A dosage–phenotype correlation would strengthen the OE conclusions; if it is absent, a comment on why would be useful.

Survival-assay design. Within the three-leaf experiment, OE lines were rewatered after 3 d of stress but KO lines after 4 d (lines 650–651). Applying a different stress duration to each genotype before scoring survival builds the expected direction into the protocol and makes the OE-vs-KO survival comparison hard to interpret as a fair one. Could the authors justify this, and where possible report survival under matched stress? The four-leaf assay uses a very different regime (30 d of withheld water, 17 d recovery; lines 654–657) — please confirm "30 d" is correct, and define what constitutes a "biological replicate" for survival (a pot? how many plants?), since the precise survival percentages imply specific denominators.

Statistical treatment. As in the hormone tables, multi-group comparisons (WT vs OE-1/-4/-5; WT vs KO-1/-2) are analyzed by ANOVA in some figures and by what appear to be pairwise tests in others, with no stated post-hoc correction (lines 722–727). Across three or more genotypes, uncorrected pairwise t-tests inflate the type-I error rate; please standardize to ANOVA with an appropriate correction (Tukey/Dunnett) and state it. For the survival data analyzed by one-way ANOVA on percentages (Fig 2H, J), please justify the test or use a model appropriate to proportions. For the RNA-seq, the DEG threshold is given inconsistently — "FC ≥ 2 / ≤ 0.5" (lines 290–291) vs "|FC| ≥ 2, P < 0.05" (lines 718–719) — and it is not clear whether the P-value is the DESeq2 adjusted P (FDR); please report the FDR used throughout, as a raw P < 0.05 is not an acceptable transcriptome-wide cutoff.

Subcellular localization and baseline physiology. The plasma-membrane assignment rests on transient N. benthamiana co-expression with a PM-RFP marker (lines 162–167). For a 274-aa protein, it would help to report the predicted topology (transmembrane domains? signal/anchor?), whether the protein is integral or peripheral, and ideally to confirm localization in a rice system (protoplasts or stable lines). Separately, soluble-sugar content is already higher in some OE lines than WT under normal conditions (lines 232–233), which complicates the drought interpretation and is worth a comment. The introduction emphasizes both SOD and POD (lines 86–88) but only SOD is measured; adding POD/CAT, and quantifying the DAB/NBT staining (Fig 3A–B) rather than describing it qualitatively, would round out the antioxidant claim.

Please make sure the references are accurately cited. At least two appear mismatched to their in-text claims and look like reference-manager errors: ref 7 (Maréchal & Brisson 2010, New Phytologist, on organelle-genome recombination) is cited for "ABA accumulation in mesophyll cells effectively improves drought resistance" (lines 57–58), and ref 57 (Buckley 2019, "How do stomata respond to water status?", New Phytologist) is cited for the "modified Yoshida nutrient solution" recipe (line 607), where the standard citation is Yoshida et al. 1976. I would recommend checking the whole bibliography against in-text usage. Also, the qPCR reference gene is named "Ubiquitin (Os01g0328400)" in the text (line 619) but "UBQ5" in Table S3 — please reconcile.

Data availability. I could not find an accession for the authors' own RNA-seq (processed by OE Biotech / OECloud); the public data retrieved from the GSA (lines 590–591) are separate. Please deposit the newly generated reads (GSA/SRA/GEO) and provide the accession, and confirm deposition of the supplementary tables.

A suggestion, not a requirement. The study is restricted to the seedling stage. It would strengthen the work, and the breeding framing in particular, to know whether the drought phenotype carries through to the adult plant and the field, and whether it has agronomic-trait costs. I appreciate this would take time and may be beyond the scope of the present manuscript; I raise it because the abstract and discussion lean on the breeding application (lines 37–38, 583–586).

Overall, this is an extensive and carefully executed phenotypic study of an interesting gene, and the nitrate-dependent root phenotype is a real contribution. My main concerns are that the comparative structure of the paper depends on two genetic backgrounds the data themselves show are not interchangeable, the hormone measurements at the core of the ABA model are unreplicated and point in opposite directions in the two wild types, the mechanism is built on an OE-only transcriptome, and the ABA-signaling logic is internally inconsistent at OsbZIP23. With same-background lines (or a clear re-scoping to within-background claims), properly replicated and reconciled hormone data, a matched KO transcriptome, ideally at least one genetic-interaction or complementation experiment, and a title and model tempered to what the data support, this would be a considerably stronger and more convincing paper.

**********

**Have all data underlying the figures and results presented in the manuscript been provided?**

Large-scale datasets should be made available via a public repository as described in the *PLOS Genetics*  data availability policy, and numerical data that underlies graphs or summary statistics should be provided in spreadsheet form as supporting information.

Reviewer #1: **No:** OsDUF1223 was identified from publicly available microarray datasets; however, the supporting data are not shown in the manuscript.

Reviewer #2: Yes

Reviewer #3: Yes

**********

PLOS authors have the option to publish the peer review history of their article (what does this mean?). If published, this will include your full peer review and any attached files.

If you choose “no”, your identity will remain anonymous but your review may still be made public.

**Do you want your identity to be public for this peer review?**  For information about this choice, including consent withdrawal, please see our Privacy Policy.

Reviewer #1: No

Reviewer #2: No

Reviewer #3: No

[NOTE: If reviewer comments were submitted as an attachment file, they will be attached to this email and accessible via the submission site. Please log into your account, locate the manuscript record, and check for the action link "View Attachments". If this link does not appear, there are no attachment files.]

**Figure resubmission:**

**Reproducibility:**

To enhance the reproducibility of your results, we recommend that authors of applicable studies deposit laboratory protocols in protocols.io, where a protocol can be assigned its own identifier (DOI) such that it can be cited independently in the future. Additionally, PLOS ONE offers an option to publish peer-reviewed clinical study protocols. Read more information on sharing protocols at https://plos.org/protocols?utm_medium=editorial-email&utm_source=authorletters&utm_campaign=protocols

# Author comment: Author response to Decision Letter 1

**DOI:** 10.1371/journal.pgen.1012295.r002

27 Jul 2026

Attachment Submitted filename: Response to Reviewers.docx

# Peer review: Decision Letter 1

**DOI:** 10.1371/journal.pgen.1012295.r003

**Authors:** Tomokazu Kawashima, Quan Wang

12 Aug 2026

PGENETICS-D-26-00390R1

OsDUF1223 negatively regulates seedling drought tolerance and modulates nitrate-dependent root growth in rice

PLOS Genetics

Dear Dr. li,

Thank you for submitting your manuscript to PLOS Genetics. After careful consideration, we feel that it has merit but does not fully meet PLOS Genetics's publication criteria as it currently stands. Therefore, we invite you to submit a revised version of the manuscript that addresses the points raised during the review process.

Please submit your revised manuscript within by Sep 11 2026 11:59PM. If you will need more time than this to complete your revisions, please reply to this message or contact the journal office at plosgenetics@plos.org. When you're ready to submit your revision, log on to https://www.editorialmanager.com/pgenetics/ and select the 'Submissions Needing Revision' folder to locate your manuscript file.

Please include the following items when submitting your revised manuscript:

* A letter that responds to each point raised by the editor and reviewer(s). You should upload this letter as a separate file labeled 'Response to Reviewers'. This file does not need to include responses to formatting updates and technical items listed in the 'Journal Requirements' section below.

* A marked-up copy of your manuscript that highlights changes made to the original version. You should upload this as a separate file labeled 'Revised Manuscript with Track Changes'.

* An unmarked version of your revised paper without tracked changes. You should upload this as a separate file labeled 'Manuscript'.

If you would like to make changes to your financial disclosure, competing interests statement, or data availability statement, please make these updates within the submission form at the time of resubmission. Guidelines for resubmitting your figure files are available below the reviewer comments at the end of this letter.

As the corresponding author, your ORCID iD is verified in the submission system and will appear in the published article. PLOS supports the use of ORCID, and we encourage all coauthors to register for an ORCID iD and use it as well. Please encourage your coauthors to verify their ORCID iD within the submission system before final acceptance, as unverified ORCID iDs will not appear in the published article. Only the individual author can complete the verification step; PLOS staff cannot verify ORCID iDs on behalf of authors.

We look forward to receiving your revised manuscript.

Kind regards,

Tomo Kawashima

Academic Editor

PLOS Genetics

Quan Wang

Section Editor

PLOS Genetics

Aimée Dudley

Editor-in-Chief

PLOS Genetics

Anne Goriely

Editor-in-Chief

PLOS Genetics

**Additional Editor Comments (if provided):**

Reviewers agree that the manuscript has been substantially improved and that the key scientific concerns raised previously have been addressed. The remaining comments focus primarily on clarification of conclusions and methodological reporting rather than new experimentation. The authors should revise the text to avoid overstating mechanistic conclusions, address the remaining logical inconsistencies highlighted by the reviewers, provide the requested clarifications regarding Figure 1 and transgenic line comparisons, and ensure that experimental design information (including sample sizes and replication) remains clearly presented and easily accessible to readers.

**Reviewers' comments:**

Reviewer's Responses to Questions

**Comments to the Authors:**

**Please note here if the review is uploaded as an attachment.**

Reviewer #1: The authors have adequately addressed my previous comments. Regarding the number of biological replicates, I recognize that my previous comment did not fully take into account the experimental design described by the authors, and I have no further concerns regarding this point.

Regarding the microarray data, although it is unfortunate that the original dataset is no longer available, I understand and accept the authors’ explanation.

The authors have also adequately addressed my concerns regarding the phylogenetic analysis of OsDUF1223 and the differences in root phenotypes under the various nitrogen conditions.

Overall, the manuscript has been appropriately revised, and I have no further major concerns.

Reviewer #2: This study characterizes the rice novel gene OsDUF1223 and its potential roles in ABA signaling, nitrogen response and drought tolerance, presenting valid preliminary findings with moderate innovation suitable for PLOS Genetics. The overall study framework is complete, but several issues regarding experimental documentation, data interpretation and logical consistency need to be addressed. Detailed comments are listed below.

1. Supplementary optimization of Figure 1-related details and phenotypic evidence

The title of Figure 1 is inaccurate and should specify the OsDUF1223 protein subcellular localization rather than the gene. The plasma membrane RFP marker used in Figure 1F lacks source and methodological description, which should be supplemented for reproducibility. In addition, the claimed “perfect overlap” between GFP and RFP signals is not fully supported by the merged image. The authors should provide magnified views or simple colocalization quantification (e.g., Pearson correlation coefficient) to solidify the subcellular localization results.

2. Standardization of transgenic material background and unified interpretation of phenotypic data

OE and KO lines were generated in Nipponbare and Zhonghua 11 backgrounds, respectively. Although both are japonica rice, intrinsic genetic background differences preclude direct phenotypic comparison between OE and KO lines; each transgenic line should only be compared with its corresponding wild-type control. The background-dependent drought variation between the two cultivars should be explicitly discussed. Furthermore, the inconsistent drought treatment duration (3 d for OE, 4 d for KO) needs a clear methodological explanation to justify the experimental rationality and result reliability.

3. Distinguish correlation and causality, and optimize the elaboration of molecular regulatory mechanisms

The manuscript proposes that OsDUF1223 integrates ABA and nitrogen signaling to regulate drought tolerance, based mainly on phenotypic and transcriptomic data. However, direct molecular evidence for physical or regulatory interactions between OsDUF1223 and key ABA/nitrogen pathway components is lacking. The authors should clearly distinguish correlation versus causation in the text and discussion, and provide explicit, testable mechanistic hypotheses, which will improve the rigor of the current model without requiring large-scale molecular validation.

4. Explain the logical contradiction of ABA-related experimental data

The conclusion that OsDUF1223 positively regulates ABA signaling conflicts with the reduced endogenous ABA level in OE lines (Table S2). In addition, the plant height response to ABA inhibitor treatment is stronger than that of exogenous ABA application, showing inconsistent ABA-related phenotypes. The authors should provide reasonable mechanistic explanations for these discrepancies based on current data and published evidence (e.g., differential regulation of ABA biosynthesis versus signaling) to reconcile the contradictory results.

5. Optimize the causal logic between root architecture and drought tolerance phenotype

The proposed model that OsDUF1223 modulates drought tolerance via nitrogen-dependent root architecture suffers from logical inconsistency. KO lines with shorter roots exhibit enhanced drought tolerance, and root length differences between OE/KO lines disappear under PEG treatment while drought tolerance differences remain. The authors should revise and refine the discussion to clarify that root architecture is one of multiple regulatory pathways rather than the sole determinant of drought response, and reasonably interpret the decoupling of root phenotype and drought tolerance to strengthen logical coherence.

Reviewer #3: The authors have addressed the comments and suggestions made by the reviewers and modified the manuscript accordingly.

However I strongly oppose removing experimental design information from the Figure legends as indicated by the authors:

"In addition, we will delete the sample size information (e.g., "n=3") from the figurelegends and uniformly incorporate this information into the Materials and Methods section to ensureconsistent and standardized presentation throughout the manuscript."

**********

**Have all data underlying the figures and results presented in the manuscript been provided?**

Large-scale datasets should be made available via a public repository as described in the *PLOS Genetics*  data availability policy, and numerical data that underlies graphs or summary statistics should be provided in spreadsheet form as supporting information.

Reviewer #1: Yes

Reviewer #2: Yes

Reviewer #3: Yes

**********

PLOS authors have the option to publish the peer review history of their article (what does this mean?). If published, this will include your full peer review and any attached files.

If you choose “no”, your identity will remain anonymous but your review may still be made public.

**Do you want your identity to be public for this peer review?**  For information about this choice, including consent withdrawal, please see our Privacy Policy.

Reviewer #1: No

Reviewer #2: No

Reviewer #3: No

[NOTE: If reviewer comments were submitted as an attachment file, they will be attached to this email and accessible via the submission site. Please log into your account, locate the manuscript record, and check for the action link "View Attachments". If this link does not appear, there are no attachment files.]

**Figure resubmission:**

**Reproducibility:**

To enhance the reproducibility of your results, we recommend that authors deposit laboratory protocols in protocols.io, where a protocol can be assigned its own identifier (DOI) such that it can be cited independently in the future. Additionally, PLOS ONE offers an option to publish peer-reviewed clinical study protocols. Read more information on sharing protocols at https://plos.org/protocols?utm_medium=editorial-email&utm_source=authorletters&utm_campaign=protocols

# Author comment: Author response to Decision Letter 2

**DOI:** 10.1371/journal.pgen.1012295.r004

16 Aug 2026

Attachment Submitted filename: Response_to_Reviewers_auresp_2.docx

# Editor report: Decision Letter 2

**DOI:** 10.1371/journal.pgen.1012295.r005

**Authors:** Tomokazu Kawashima, Quan Wang

19 Aug 2026

Dear Dr li,

We are pleased to inform you that your manuscript entitled "OsDUF1223 negatively regulates seedling drought tolerance and modulates nitrate-dependent root growth in rice" has been editorially accepted for publication in PLOS Genetics. Congratulations!

Before your submission can be formally accepted and sent to production you will need to complete our formatting changes, which you will receive in a follow up email. Please be aware that it may take several days for you to receive this email; during this time no action is required by you. Please note: the accept date on your published article will reflect the date of this provisional acceptance, but your manuscript will not be scheduled for publication until the required changes have been made.

Once your paper is formally accepted, an uncorrected proof of your manuscript will be published online ahead of the final version, unless you’ve already opted out via the online submission form. If, for any reason, you do not want an earlier version of your manuscript published online or are unsure if you have already indicated as such, please let the journal staff know immediately at plosgenetics@plos.org.

In the meantime, please log into Editorial Manager at https://www.editorialmanager.com/pgenetics/, click the "Update My Information" link at the top of the page, and update your user information to ensure an efficient production and billing process. Note that PLOS requires an ORCID iD for all corresponding authors. Therefore, please ensure that you have an ORCID iD and that it is validated in Editorial Manager. To do this, go to ‘Update my Information’ (in the upper left-hand corner of the main menu), and click on the Fetch/Validate link next to the ORCID field.  This will take you to the ORCID site and allow you to create a new iD or authenticate a pre-existing iD in Editorial Manager.

If you have a press-related query, or would like to know about making your underlying data available (as you will be aware, this is required for publication), please see the end of this email. If your institution or institutions have a press office, please notify them about your upcoming article at this point, to enable them to help maximise its impact. Inform journal staff as soon as possible if you are preparing a press release for your article and need a publication date.

Thank you again for supporting open-access publishing; we are looking forward to publishing your work in PLOS Genetics!

Yours sincerely,

Tomokazu Kawashima

Academic Editor

PLOS Genetics

Quan Wang

Section Editor

PLOS Genetics

Aimée Dudley

Editor-in-Chief

PLOS Genetics

Anne Goriely

Editor-in-Chief

PLOS Genetics

www.plosgenetics.org

BlueSky: @plos.bsky.social

----------------------------------------------------

Comments from the reviewers (if applicable):

----------------------------------------------------

**Data Deposition**

If you have submitted a Research Article or Front Matter that has associated data that are not suitable for deposition in a subject-specific public repository (such as GenBank or ArrayExpress), one way to make that data available is to deposit it in the Dryad Digital Repository. As you may recall, we ask all authors to agree to make data available; this is one way to achieve that. A full list of recommended repositories can be found on our website.

The following link will take you to the Dryad record for your article, so you won't have to re‐enter its bibliographic information, and can upload your files directly:

http://datadryad.org/submit?journalID=pgenetics&manu=PGENETICS-D-26-00390R2

More information about depositing data in Dryad is available at http://www.datadryad.org/depositing. If you experience any difficulties in submitting your data, please contact help@datadryad.org for support.

Additionally, please be aware that our data availability policy requires that all numerical data underlying display items are included with the submission, and you will need to provide this before we can formally accept your manuscript, if not already present.

----------------------------------------------------

**Press Queries**

If you or your institution will be preparing press materials for this manuscript, or if you need to know your paper's publication date for media purposes, please inform the journal staff as soon as possible so that your submission can be scheduled accordingly. Your manuscript will remain under a strict press embargo until the publication date and time. This means an early version of your manuscript will not be published ahead of your final version. PLOS Genetics may also choose to issue a press release for your article. If there's anything the journal should know or you'd like more information, please get in touch via plosgenetics@plos.org.

# Editor report: Acceptance letter

**DOI:** 10.1371/journal.pgen.1012295.r006

**Authors:** Tomokazu Kawashima, Quan Wang

PGENETICS-D-26-00390R2

OsDUF1223 negatively regulates seedling drought tolerance and modulates nitrate-dependent root growth in rice

Dear Dr li,

We are pleased to inform you that your manuscript entitled "OsDUF1223 negatively regulates seedling drought tolerance and modulates nitrate-dependent root growth in rice" has been formally accepted for publication in PLOS Genetics! Your manuscript is now with our production department and you will be notified of the publication date in due course.

The corresponding author will soon be receiving a typeset proof for review, to ensure errors have not been introduced during production. Please review the PDF proof of your manuscript carefully, as this is the last chance to correct any errors. Please note that major changes, or those which affect the scientific understanding of the work, will likely cause delays to the publication date of your manuscript.

Soon after your final files are uploaded, unless you have opted out or your manuscript is a front-matter piece, the early version of your manuscript will be published online. The date of the early version will be your article's publication date. The final article will be published to the same URL, and all versions of the paper will be accessible to readers.

For Research Articles, you will receive an invoice from PLOS for your publication fee after your manuscript has reached the completed accept phase. If you receive an email requesting payment before acceptance or for any other service, this may be a phishing scheme. Learn how to identify phishing emails and protect your accounts at https://explore.plos.org/phishing.

Thank you again for supporting PLOS Genetics and open-access publishing. We are looking forward to publishing your work!

With kind regards,

Sharmila Kamatchi

PLOS Genetics

On behalf of:

The PLOS Genetics Team

Carlyle House, Carlyle Road, Cambridge CB4 3DN | United Kingdom

plosgenetics@plos.org | +44 (0) 1223-442823

plosgenetics.org | Twitter: @PLOSGenetics
